# Human Milk Macronutrients and Child Growth and Body Composition in the First Two Years: A Systematic Review

**DOI:** 10.1016/j.advnut.2023.100149

**Published:** 2023-11-18

**Authors:** Meredith (Merilee) Brockway, Allison I. Daniel, Sarah M. Reyes, Matthew Granger, Joann M. McDermid, Deborah Chan, Rebecca Refvik, Karanbir K. Sidhu, Suad Musse, Pooja P. Patel, Caroline Monnin, Larisa Lotoski, Donna Geddes, Fyezah Jehan, Patrick Kolsteren, Lindsay H. Allen, Daniela Hampel, Kamilla G. Eriksen, Natalie Rodriguez, Meghan B. Azad

**Affiliations:** 1 Manitoba Interdisciplinary Lactation Centre (MILC), Children’s Hospital Research Institute of Manitoba, University of Manitoba, Canada; 2 Department of Pediatrics and Child Health, University of Manitoba, Canada; 3 Faculty of Nursing, University of Calgary, Canada; 4 Centre for Global Child Health, Hospital for Sick Children, Canada; 5 Department of Nutritional Sciences, Temerty Faculty of Medicine, University of Toronto, Canada; 6 Department of Food and Human Nutritional Sciences, University of Manitoba, Canada; 7 Consultant, Charlottesville, USA; 8 Department of Epidemiology, Biostatistics, and Occupational Health, McGill University, Canada; 9 Department of Public Health and Community Medicine, Tufts University School of Medicine, USA; 10 Neil John Maclean Health Sciences Library, University of Manitoba, Canada; 11 School of Molecular Sciences, The University of Western Australia, Australia; 12 Department of Pediatrics, Aga Khan University, Pakistan; 13 Department of Food Safety and Food Quality, Ghent University, Belgium; 14 Western Human Nutrition Research Center, Agriculture Research Service, United States Department of Agriculture, USA; 15 Department of Nutrition, University of California, Davis, USA; 16 Department of Nutrition, Exercise and Sports, University of Copenhagen, Denmark

**Keywords:** human milk, breastmilk, breastfeeding, infant, anthropometry, macronutrients, carbohydrates, lactose, glucose, protein, amino acids, fat, fatty acids, body composition, growth, lactation

## Abstract

Among exclusively breastfed infants, human milk (HM) provides complete nutrition in the first mo of life and remains an important energy source as long as breastfeeding continues. Consisting of digestible carbohydrates, proteins, and amino acids, as well as fats and fatty acids, macronutrients in human milk have been well studied; however, many aspects related to their relationship to growth in early life are still not well understood. We systematically searched Medline, EMBASE, the Cochrane Library, Scopus, and Web of Science to synthesize evidence published between 1980 and 2022 on HM components and anthropometry through 2 y of age among term-born healthy infants. From 9992 abstracts screened, 57 articles reporting observations from 5979 dyads were included and categorized based on their reporting of HM macronutrients and infant growth.

There was substantial heterogeneity in anthropometric outcome measurement, milk collection timelines, and HM sampling strategies; thus, meta-analysis was not possible. In general, digestible carbohydrates were positively associated with infant weight outcomes. Protein was positively associated with infant length, but no associations were reported for infant weight. Finally, HM fat was not consistently associated with any infant growth metrics, though various associations were reported in single studies. Fatty acid intakes were generally positively associated with head circumference, except for docosahexaenoic acid. Our synthesis of the literature was limited by differences in milk collection strategies, heterogeneity in anthropometric outcomes and analytical methodologies, and by insufficient reporting of results. Moving forward, HM researchers should accurately record and account for breastfeeding exclusivity, use consistent sampling protocols that account for the temporal variation in HM macronutrients, and use reliable, sensitive, and accurate techniques for HM macronutrient analysis.

**Statement of Significance**

Our work comprehensively synthesizes evidence regarding associations between individual HM macronutrients and child anthropometrics among healthy, term-born infants. This manuscript is part of a larger 3-part systematic review (PROSPERO: CRD42020187350).

## Introduction

Human milk (HM) is the ideal nutritional source for infants. The WHO recommends that infants are fed an exclusive HM diet for 6 mo and that HM feeding is sustained to 2 y and beyond [1]. HM contains a multitude of components, including macronutrients that provide energy for infant growth and development. Consisting of approximately 87% water; the remaining 13% of HM consists primarily of macronutrients (carbohydrates, proteins, and fats) [2,3]—the major sources of energy for infant growth. Exclusively breastfed infants derive almost all their energy from carbohydrates (45%; including lactose, glucose, fructose) and fats (44%), whereas proteins contribute about 8% [4]. In addition to this growth-promoting energy, HM macronutrients provide amino acids and fatty acids (FAs), both of which are important for metabolic processes, immunity, and infant development.

Carbohydrates are the most abundant nonaqueous component in milk, making up about 7% of total HM volume. Consisting of 3 chemical groups (monosaccharides, disaccharides, and oligosaccharides), only 4.6 to 6.0% of HM carbohydrates are digestible [2]. The role of nondigestible carbohydrates, known as human milk oligosaccharides (HMOs), in infant growth is reviewed in a companion manuscript [5]. Digestible carbohydrates in HM predominantly consist of lactose (67–78 mg/mL) [6] and glucose (180 – 330 μg/mL) [7] and are an important source of energy, with a caloric density of 4.0 kcal/g [8]. Maternal diet appears to have minimal influence on HM carbohydrate composition [9], with the exception of fructose, which demonstrate increased concentrations in mothers with high-sugar diets [7,10]. Mothers who produce higher volumes of milk tend to have higher concentrations of lactose compared with mothers who produce lower volumes of milk [11,12]. There is conflicting evidence on the role of HM carbohydrates in somatic infant growth [11], although emerging evidence suggests that increased HM fructose levels may result in accelerated infant growth [10].

Fat accounts for about 3 to 4% of total HM volume and 40 to 50% of caloric intake and has a caloric density of 9.0 kcal/g (4). Total lipid content is positively associated with maternal BMI [13] and can be affected by diet [12]. Lipid content also varies depending on the time of day [14,15] as well as the timing within each breastfeeding session, with foremilk having significantly lower total lipid content than hind milk [16]. As such, it is ideal to sample human milk across a 24-h period, throughout a feeding, and to weigh infants before and after their feed to properly reflect total lipid intake [15]. Human milk fat composition, consisting primarily of triglycerides, free FAs, and cholesterol, is highly variable among females [4] and associated with dietary, genetic, sociodemographic, and environmental factors [17]. Fat content is important for brain growth and development, and certain FAs (FA) are associated with neurodevelopment [18] and cardiovascular health [19]. However, there is a paucity in the literature compiling evidence on HM fat composition and infant growth [20].

Proteins make up 1% of total HM volume and have a caloric density of 4.0 kcal/g [8]. Thousands of proteins are found in HM, and the most abundant can be classified into 3 categories: casein, whey, and mucins [21]. Human milk protein consists of about 60% whey and 40% casein, whereas low abundance mucins are present as milk fat globule membranes [21]. Proteins found in HM are important for nutritive growth, usually in the form of casein proteins and amino acids. Human milk can be analyzed for crude or true protein. Crude protein is calculated based on the total amount of nitrogen in a sample, of which 20 to 25% is nonprotein nitrogen [22], whereas true protein is a corrected value based on the content of actual protein [23]. This distinction between crude and true protein is also important to consider when analyzing amino acids because free amino acids account for 8 to 22% of nonprotein nitrogen [24]. Specific bioactive proteins that are important for non-nutritive development [21] (such as lactoferrin, secretory IgA, and lysozyme) are reviewed in Brockway et al. (2023) [5]. Previous reviews [25] indicate that evidence is inconclusive about the role of protein and amino acids in infant growth. Positive associations have been reported between protein intake from all sources (HM, formula, and complementary foods) and infant growth in the first 2 y of life; however, this research was conducted predominantly on children who received cow milk-based formulas [26]. Cow milk formulas have higher protein concentrations compared to HM (∼2.2 versus ∼1.5 g/100 kcal) [27], and infant consumption of formula increases as the infant grows, which does not happen to the same extent as breastfeeding [28]. To date, minimal research has been conducted on protein intake among exclusively HM-fed full-term infants, who have different growth trajectories compared with formula fed infants [27].

This systematic review aims to assess and synthesize evidence on the associations between HM components and child anthropometry measured in the first 2 y. Due to the large number of articles retrieved, results were organized into 3 manuscripts encompassing the following categories: micronutrients (vitamins and minerals [6]), bioactive components (e.g., cytokines, hormones, and nondigestible carbohydrates) and the current manuscript, macronutrients (lipids, proteins, and digestible carbohydrates [8]).

## Methods

This review is registered with PROSPERO: CRD42020187350 and is reported according to the Preferred Reporting Items for Systematic Reviews and Meta-Analyses (PRISMA) [29] and the Systematic Review without Meta-Analysis (SWiM) guidelines [30]. Eight reviewers (SMR, JMM, DC, MG, KS, SM, RR, and MB) independently participated in abstract and full-text screening, quality assessment, and data extraction. Covidence Systematic Review Software (2020) was used to manage screening and data extraction [31].

### Search Strategy & Screening

The search strategy and screening, selection criteria, quality assessment, data extraction, and analytic techniques are described in Reyes et al. (2023) [32]. Briefly, the original search was created in Medline (Ovid) and translated to the other databases. We searched the following databases in March 2020: Medline (Ovid; Medline All 1946-2020), EMBASE (Ovid; 1974-2020), the Cochrane Library (Wiley; CENTRAL and Cochrane Database of Systematic Reviews), Scopus (1970-2020), and Web of Science Core Collection (Clarivate, 1900-2020). References published in English and after 1980 were included. The Medline (Ovid) strategy is available in Appendix A*.* All other strategies are available upon request. Grey literature was located via Agricola, PEN (Practice-based Evidence in Nutrition), OpenSIGLE, Google Advanced, and Prospero. Finally, a hand search was conducted of review articles identified with our search strategy to identify any studies missed in the search strategy mentioned above. An updated search was conducted in March 2022, revisiting all the original databases and grey literature sources to ensure inclusion of newly published articles. All records were screened in duplicate in Covidence (Veritas Health Innovation, Melbourne, Australia) by 2 independent reviewers.

### Selection Criteria

Randomized controlled trials (RCTs) or observational studies were eligible for inclusion; however, data from RCTs were evaluated as observational studies because, in all cases, associations between HM composition and infant anthropometrics were secondary trial outcomes. Inclusion criteria were studies reporting on healthy, term, breastfed infants (aged 0 to 24 mo). Whereas breastfeeding exclusivity was not an inclusion criterion, it was recorded when reported by authors **(**Table 1**)** [[33], [34], [35], [36], [37], [38], [39], [40], [41], [42], [43], [44], [45], [46], [47], [48], [49], [50], [51], [52], [53], [54], [55], [56], [57], [58], [59], [60], [61], [62], [63], [64], [65], [66], [67], [68], [69], [70], [71], [72], [73], [74], [75], [76], [77], [78], [79], [80], [81], [82], [83], [84], [85], [86], [87], [88], [89]] and considered in the quality assessment (described below). *Healthy* was defined as term birth (37 wk, 0 d of gestation or later) with no congenital or other morbidities and no admission in the neonatal intensive care unit.

**TABLE 1:** Detailed characteristics and results of included studies reporting on human milk macronutrients and infant anthropometrics - organized alphabetically by study first author. Alternative versions organized by component available in the Supplemental Tables 3–5.

Authors, country, publication y (Income setting)	Design and participants	Milk sampling time(s), analytes and units	Anthropometric outcome assessment time(s), measures, standards	Associations∗∗	Major confounders considered
Abdelhamid et al. Egypt, 2020 (LMIC) [51]	Cross-sectional 100 infants	6–4 mo fat, protein, lactose (concentrations) Exclusive BF only	6–14 mo weight, length, BMI	(-) Association for HM Fat and BMI (No) Association for HM fat and length or weight (No) Association for HM protein and length, weight or BMI (No) Association for HM lactose and length, weight or BMI	exclusive BF

Aksit et al. Turkey, 2002 (LMIC) [65]	Cross-sectional 80 infants	2 mo fat - via creamatocrit: % cream (concentrations) Exclusive BF only	2 mo high or low weight gain	*Difference from birth to 2 mo* (-) Association for fat and weight gain	exclusive BF

Babiszewska et al.Poland, 2020(HIC) [82]	Cross-sectional 60 infants	3–6 mo fatty acids: linoleic acid, alpha-linolenic acid (concentrations) Exclusive BF only	3–6 mo HC, head volume cranial indices (breadth/length, height/breadth, height/length)	(+) Association for linoleic acid and head volume (+) Association for alpha-linolenic acid and cranial height/length ratio	exclusive BF, infant sex, age; maternal socioeconomic status, cranial indices

Baldeón et al. Ecuador, 2019 (LMIC) [58]	Longitudinal 65 infants enrolled 61 analyzed at 1 wk 47 analyzed at 2 wk 38 analyzed at 2 mo 37 analyzed at 4 mo	1 wk, 2 wk, 2 mo, 4 mo amino acids (concentrations) Exclusive BF only	1 wk, 2 wk, 2 mo, 4 mo weight gain tertiles, HC gain tertiles	*Difference from 1 wk and 4 mo* (+) Association for glutamic acid and weight gain (+) Association for alanine and weight gain	exclusive BF, infant sex

Brown et al.Bangladesh, 1986(LMIC) [62]	Longitudinal 60 infants enrolled 58 analyzed	Monthly between birth, 9 mo (starting at different times depending on infant age at recruitment) protein: total nitrogen (measured daily intake)	monthly between birth, 9 mo (starting at different times depending on infant age at recruitment) WAZ, WLZ, LAZ (NCHS standards)	*Under 3 mo timepoints* (+) Association for protein (nitrogen) intake and WAZ (+) Association for protein (nitrogen) intake and WLZ (not included in results due to inaccurate protein measurement method)	none reported

Cheema et al. Australia, 2021(HIC) [34]	Cross-Sectional 67 infants (57 analyzed)	2 mo glucose, lactose (concentrations and intakes) Exclusive BF only	3 mo weight, length, BMI, HC, FFM, FFMI, FM, FMI, %FM, FM/FFM, z-scores (WHO standards)	(+) Association for lactose (CDI) and weight and length, adiposity, lean body mass (FFM and FFMI) and WAZ (No) Association for glucose and anthropometrics	exclusive BF, maternal age. ethnicity, parity, mode of delivery, height, weight, gestational age, sex, birth weight, birth length,

Cisse et al.Senegal, 2002(LMIC) [63]	Randomized controlled trial 133 infants	2 wk protein (daily intake)	3 mo weight, WLZ, LAZ (NCHS standards)	(+) Association for protein intake and weight (+) Association for protein intake and WLZ (study group-dependent) (+) Association for protein intake and LAZ	none reported

de Fluiter et al. Netherlands, 2021 (HIC) [35]	Cohort 133 infants	1, 3 mo fat, protein: crude, true; carbohydrates (concentrations) Exclusive BF only	1, 3, 6, 9, 12, 18, 24 mo weight, length, HC, WFL, WA, HFA (SDs), FMI, body composition using air-displacement plethysmography (ADPby PEAPOD**)**, abdominal fat mass (Online growth analyzer to determine growth standards)	*3 mo timepoint* (+) Association for HM fat (g/100 ml) and subcutaneous FM (cm) at 3 mo (+) Association for HM fat (g/100 ml) at 3 mo and change in FM% SDS from 1 to 6 mo (No) Association for HM Protein and weight, length, HC, WFL, WA, HFA (SDs), FMI, body composition, abdominal fat mass (No) Association for HM carbohydrates and weight, length, HC, WFL, WA, HFA (SDs), FMI, body composition, abdominal fat mass *6 mo timepoint* (+) Association for HM fat at 3 mo and FM% at 6 mo (-) Association for HM crude and true protein at 3 mo and visceral FM at 6 mo (No) Association for HM Protein and anthropometrics (No) Association for HM carbohydrates and weight, length, HC, WFL, W-A, HFA (SDs), FMI, body composition, abdominal fat mass	exclusive BF during first 3 mo

De la Garza Puentes et al.Spain, 2019(HIC) [78]	Longitudinal (subset of cohort) 78 infants	2–4 d (colostrum), 28–32 d (mature milk) fatty acids (concentrations) Mixed feeding	6, 18 mo BMIZ, WAZ, LAZ (WHO standards)	*6 mo timepoint (colostrum sample)* (-) Association for ARA and BMIZ (-) Association for EPA and BMIZ (-) Association for DHA and BMIZ (-) Association for n-6 LCPUFA and BMIZ (-) Association for n-3 LCPUFA and BMIZ (-) Association for n-3 PUFA and BMIZ (+) Association for n6:n3 PUFA and BMIZ (+) Association for linoleic acid and WAZ (+) Association for n6:n3 PUFA and WAZ *6 mo timepoint (mature milk sample)* (+) Association for linoleic acid and WAZ (+) Association for n-6:n-3 PUFA and WAZ	exclusive BF, infant sex; maternal BMI, weight gain during pregnancy, smoking education,

De Luca et al. France, 2016 (HIC) [56]	Longitudinal 165 infants enrolled 100 analyzed	Birth, 1 mo fat, protein (concentrations) Exclusive BF only	Birth, 1 mo weight, length	*1 mo timepoint* (+) Association for protein and weight (+) Association for protein and length *Difference from birth to 1 mo* (+) Association for fat and weight gain (+) Association for protein and length gain	exclusive BF, unadjusted estimates provided by authors

Dewey et al.United States, 1993(HIC) [71]	Longitudinal 92 infants enrolled 46 analyzed	3, 6, 9, 12 mo fat (concentrations) Mixed feeding	monthly from 1 to 18 mo, then 21, 24 mo WLZ, skinfold thickness body composition (fat mass % using prediction equations) (NCHS standards)	None	none reported

Ding et al.China, 2021(LMIC) [84]	Cross-sectional 121 Infants	30–50 d fatty acids (PCAs only) (concentrations) Exclusive BF only	30–50 d weight, length, BMI, HC, LAZ, WAZ, HCAZ, WLZ, WAZ (WHO standards)	Only PCA patterns reported, not individual FAs. (+) Association for pattern 1 (C18:0, C14:0, C16:0, C18:1, C18:2, C16:1, C10:0, C20:4, C14:1, C16:2 and C12:0) and LAZ, WAZ and HCAZ (+) Association for pattern 4 (C20:3, C22:4, C22:5, and C4:0) and LAZ, WAZ and HCAZ	exclusive BF, age, height, prepregnancy weight, prenatal weight, gestational age, parity, delivery mode, diet

Dorea et al. Brazil, 1993 (LMIC) [57]	Longitudinal 8 infants	Bi-wkly or monthly between birth, 6 mo fat, protein: total nitrogen (concentrations)	Bi-wkly or monthly between birth, 6 mo weight, height	*Difference from birth to 6 mo* (+) Association for protein and weight gain	Zinc, total nitrogen, and fat in multiple regression

Ellsworth et al. USA, 2020 (HIC) [46]	Longitudinal 55 infants enrolled 32 analyzed	2 wk fat, fatty acids (n-6:n-3 LCPUFA), protein, carbohydrates (concentrations) Mixed feeding	2 wk, 2 mo WLZ, BMIZ, WAZ, LAZ, HC (WHO standards)	*Difference from 2 wk to 2 mo* (+) Association for n-6:n-3 LCPUFA and WLZ increase (+) Association for n-6:n-3 LCPUFA and BMIZ increase (+) Association for fat and WAZ increase (exclusively BF infants only) (+) Association for n-6:n-3 LCPUFA and WAZ increase	infant sex exclusive BF

Enstad et al. USA, 2020 (HIC) [36]	Longitudinal 40 infants	1, 4 mo fatty acids: n-6:n-3 PUFA (concentrations) Exclusive BF only	monthly between 1, 7 mo WAZ, BMIZ, LAZ, body composition (fat mass %, lean mass % using X-ray absorptiometry [DXA] scans) (WHO standards)	*4 mo timepoint* (+) Association for n-6:n-3 PUFA and length z-scores *7 mo timepoint* (+) Association for n-6:n-3 PUFA and BMIZ *Difference for 1 and 7 mo* (+) Association for n-6:n-3 PUFA and weight z-score increase (+) Association for n-6:n-3 PUFA and BMIZ increase (+) Association for n-6:n-3 PUFA and length z-score increase	infant sex, age; maternal BMI, ethnicity

Fields et al. United States, 2012 (HIC) [37]	Longitudinal 37 infants enrolled 30 analyzed	1 mo glucose (concentrations) Exclusive BF only	1, 6 mo weight, length, body composition (fat mass, fat-free mass, trunk fat mass, fat mass % using Lunar iDXA v11-30.062 (Infant whole body analysis scanner)	None	infant sex, age, body composition at 1 mo; maternal prepregnancy BMI category

Fornes et al.Brazil, 1995(LMIC) [66]	Longitudinal 39 infants	Bi-wkly intervals between 2 wk, 3 mo fat (concentrations) Exclusive BF only	Bi-wkly intervals between 2 wk, 3 mo weight, length	None	exclusive BF

George et al. 2021, Australia (HIC) [67]	Longitudinal 11 infants	1, 3 mo HM fat globules (concentrations and intakes) Exclusive BF only	Birth, 1, 2, 3, 4, 5, 6 mo weight, length, WLZ, HC, HCAZ (WHO standards)	(+) Association for HM Cer d19: 1/22:0 and HC (+) Association for HM PI 38:5 and WLZ	exclusive BF

George et al. 2021, Australia (HIC) [85]	Longitudinal 30 infants (18 analyzed)	Birth, 1, 2, 3, 4, 5, 6 mo Fatty Acids (concentrations and intakes) Exclusive BF only	Birth, 1, 2, 3, 4, 5, 6 mo weight, length, HC, WLZ, HCZ, BMI (WHO standards)	*Moly intake and growth (adjusted for multiple comparisons)* (+) Association for total lipids and HCZ, WLZ, weight, and BMI (+) Association for hexanoic acid and HCZ, HC, weight, length (+) Association for decanoic acid and BMI (+) Association for undecanoic acid and HCZ, WLZ, weight, and BMI (+) Association for dodecanoic acid and WLZ and BMI (+) Association for tridecanoic acid and HCZ,WLZ, weight, and BMI (+) Association for tetradecanoic acid and HCZ, WLZ, and BMI (+) Association for pentadecanoic acid and HCZ, weight, length and BMI (+) Association for myristoleic acid and HCZ, HC, weight, length and BMI (+) Association for palmitic acid and HCZ, WLZ, weight, and BMI (+) Association for Cis-10-pentadecanoic acid and HCZ, WLZ, weight, BMI (+) Association for 7-hexadecanoic acid and HCZ, weight, and BMI (+) Association for heptadecanoic acid acid and HCZ, WLZ, weight, BMI (No) Association for octadecanoic acid and growth (+) Association for elaidic acid and HCZ, HC, weight, length and BMI (+) Association for cis-9-octadecanoic acid and HCZ, WLZ weight, BMI (+) Association for 11-octadecanoic acid and, WLZ weight, and BMI (+) Association for trans-9, trans-12 octadecadienic acid and HCZ, HC, weight, length and BMI (+) Association for cis-9, trans-12 octadecaduenuc acid and HCZ, HC, weight, length and BMI (+) Association for cis-9, cis-12 octadecadienic acid and WLZ weight, BMI (+) Association for heneicosanoic acid and HCZ and HC (+) Association for linolenic acid and HCZ (+) Association for cis-11, cis-14 eisosadienoic acid and HCZ (+) Association for Cis-11,14,17-eicasatrienoic acid and HCZ, HC, weight (+) Association for cis-13,16-docosadienoic acid and weight and length (No) Association for ARA, cis-15-tetracosanoic acid,9-octadecenoic acid, arachidic acid, trans-9, cis-12 octadecadienic acid, cis-11,eicosenoic acid, Y-linoleic acid, docosanoic acid, erucic acid, tricosanoic acid, cis-8,11,14-eicasatrienoic acid, tetracosanoic acid, 6-octadecanoic acid, cis-5,8,11,14,17-eicosapentanoic acid, trans-13-octadecenoic acid, cis-7,10,13,16-docosatetraenoic acid, or 4,7,10,13,16,19-DHA and HCZ, HC, WLZ weight, length and BMI Intake and growth at 6 mo (adjusted for multiple comparisons) (+) Association for total lipids and weight, (+) Association for hexanoic acid and weight (+) Association for undecanoic acid and weight (+) Association for tridecanoic acid and weight (+) Association for tetradecanoic acid and HCZ (+) Association for pentadecanoic acid and HCZ, HC (+) Association for myristoleic acid and HCZ, HC (+) Association for cis-10-pentadecanoic acid and weight, (+) Association for heptadecanoic acid acid and HCZ, weight, (+) Association for octadecanoic acid and Weight (+) Association for elaidic acid and HCZ, HC, (+) Association for cis-9-octadecanoic acid and weight, (+) Association for trans-9, trans-12 octadecadienic acid and HCZ, HC, (+) Association for cis-9, trans-12 octadecaduenuc acid and HC (+) Association for cis-11,eicosenoic acid and weight (+) Association for heneicosanoic acid and HC (+) Association for cis-11, cis-14 eisosadienoic acid and HCZ, HC, weight (No) Association for erucic acid, tricosanoic acid, cis-8,11,14-eicasatrienoic acid, Cis-11,14,17-eicasatrienoic acid, ARA, tetracosanoic acid, 6-octadecanoic acid, cis-13,16-docosadienoic acid, cis-15-tetracosanoic acid, 9-octadecenoic acid, cis-5,8,11,14,17-eicosapentanoic acid, trans-13-octadecenoic acid, cis-7,10,13,16-docosatetraenoic acid, 4,7,10,13,16,19-DHA, octanoic acid, decanoic acid, dodecanoic acid, palmitic acid, 7-hexadecanoic acid, 11-octadecanoic acid, arachidic acid, trans-9, cis-12 octadecadienic acid, cis-9, cis-12 octadecadienic acid, Y-linoleic acid, docosanoic acid, or linonleic acid and HCZ, HC, WLZ weight, length and BMI	exclusive BF

Goran et al. USA, 2017 (HIC) [7]	Longitudinal 37 infants enrolled 25 analyzed	1, 6 mo lactose, glucose, galactose (concentrations) Exclusive BF only	1, 6 mo weight, length, WLZ body composition (lean mass, fat mass, fat mass % using Lunar iDXA [General Electric, Fairfield, CT, USA]) (WHO standards)	*6 mo timepoint (hierarchical regression model)* (+) Association for fructose and weight (+) Association for fructose and WLZ (+) Association for fructose and lean mass (+) Association for fructose and fat mass	infant sex, weight; maternal prepregnancy BMI

Gridneva et al. Australia, 2018 (HIC) [86] Gridneva et al. Australia, 2019 (HIC) [42] Gridneva et al. Australia, 2021 [44] (HIC) Gridneva et al. Australia, 2022 (HIC) [45]	Longitudinal 22 infants enrolled 20 analyzed	2 and/or 5, 9, 12 mo protein, whey, casein, carbohydrates, lactose (concentrations and intakes) Exclusive BF only	2 and/or 5, 9, 12 mo weight, BMI, length, HC, body composition (fat mass, fat-free mass, fat mass index, fat-free mass index, fat mass % using ImpediMed SFB7 bioelectrical impedance analyzer [ImpediMed, Brisbane, QLD, Australia]) subcutaneous-abdominal depth, visceral depth, visceral/subcutaneous-abdominal depths ratio, Preperitoneal fat area, Subcutaneous-abdominal depth, Subcutaneous-abdominal fat area, Preperitoneal/subcutaneous-abdominal fat areas ratio	*2 mo timepoint* (+) Association for carbohydrates and fat mass (+) Association for carbohydrates and fat mass index (+) Association for carbohydrates and fat mass % *5 mo timepoints* (-) Association for carbohydrates and fat mass (-) Association for carbohydrates and fat mass index (-) Association for carbohydrates and fat mass % *9 mo timepoints* (-) Association for carbohydrates and fat mass (-) Association for carbohydrates and fat mass index (-) Association for carbohydrates and fat mass % *12 mo timepoints* (-) Association for carbohydrates and fat mass (-) Association for carbohydrates and fat mass index (-) Association for carbohydrates and fat mass % *All timepoints up to 12 mo (linear mixed effects model accounting for mo)* (+) Association for carbohydrates and weight (+) Association for carbohydrates and length (+) Association for carbohydrates and fat-free mass (+) Association for carbohydrates and fat-free mass index (+) Association for carbohydrate intake and BMI (+) Association for casein intake and fat mass (+) Association for carbohydrate intake and fat mass (+) Association for lactose intake and fat mass (-) Association for casein intake and fat-free mass (+) Association for casein intake and fat mass index (+) Association for carbohydrate intake and fat mass index (+) Association for lactose intake and fat mass index (-) Association for carbohydrate intake and fat-free mass index (-) Association for lactose intake and fat-free mass index (+) Association for carbohydrate intake and fat mass % (+) Association for lactose intake and fat mass % (+) Association for total carbohydrate and subcutaneous fat area	exclusive BF; infant sex, age

Isganaitis et al. USA, 2019 (HIC) [38]	Longitudinal 37 infants enrolled 31 analyzed at 1 mo 26 analyzed at 6 mo	1, 6 mo lipids, amino acids, carbohydrates (concentrations) Intention to Exclusively BF	1, 6 mo weight, body composition (fat mass %, fat accrual from 1 to 6 mo using DXA [Lunar scanner, GE Healthcare])	*1 mo timepoint* (-) Association for arginine and weight (-) Association for lysine and weight (-) Association for methionine and weight (-) Association for proline and weight (-) Association for DHA and weight (+) Association for glutamine and fat mass % (+) Association for threonine and fat mass % (-) Association for arginine and fat mass % *6 mo timepoint* (-) Association for glycine and fat mass % (-) Association for lysine and fat mass % (-) Association for EPA and fat mass % (-) Association for linoleic acid or alpha-linolenic acid and fat mass % (-) Association for cholesterol and fat mass % (+) Association for mannose and fat mass % *Difference for 1 and 6 mo* (+) Association for ornithine and fat accrual (-) Association for β-alanine and fat accrual (-) Association for lysine and fat accrual	infant sex, gestational age, birth weight; maternal parity

Jacobson et al. Canada, 2008 (HIC) [74]	Longitudinal 109 infants enrolled 74 analyzed at 6 mo 67 analyzed at 12 mo	6 mo, 1 y fatty acids (concentrations) Mixed Feeding	6 mo, 1 y weight, length, HC	*6 mo timepoint* (+) Association for eicosenoic acid and weight (+) Association for docosapentaenoic-n3 acid and weight (+) Association for EPA and weight (+) Association for docosapentaenoic-n3 acid and length (+) Association for EPA and length (+) Association for gamma-linolenic acid and HC (+) Association for docosapentaenoic-n3 acid and HC (+) Association for EPA and HC (-) Association for DHA intake and weight (No) Association for DHA intake and length (No) Association for DHA intake and HC *1 y timepoint* (-) Association for capric acid and weight (-) Association for capric acid and HC (-) Association for lauric acid and HC (-) Association for myristic acid and HC (+) Association for gamma-linolenic acid and HC (-) Association for DHA intake and weight (No) Association for DHA intake and length (No) Association for DHA intake and HC	Exclusive BF unadjusted estimates provided by authors

Janas et al. United States, 1986 (HIC) [64]	Longitudinal 10 infants	1, 2 mo amino acid (daily intake) Exclusive BF only	1, 2 mo weight	None	none reported

Kon et al. Russia, 2014 (LMIC) [52]	Longitudinal 103 infants enrolled 99 analyzed	1, 2, 3 mo fat, fat intake, protein protein intake (concentrations)	1, 2, 3 mo weight (categorized: low, normal, high weight gain)	*3 mo timepoint* (+) Association for fat intake and weight gain	none reported

Larnkjaer et al. Denmark, 2016 (HIC) [61]	Cross-Sectional 78 infants enrolled 50 analyzed	4 mo amino acids: glutamic acid, glutamine (concentrations) Mixed Feeding	4 mo weight, length, BMI	(+) Association for glutamine and length (not adjusted for birth length)	infant sex infant age infant birth anthropometry

Larson-Meyer et al. United States, 2021 (HIC) [72]	Longitudinal 24 infants enrolled 22 analyzed at 1 mo 15 analyzed at 6 mo	1, 6 mo Fat (fore, hind milk) (concentrations) Exclusive BF only	1, 6, 12 mo weight, WLZ, WAZ (WHO standards)	*1 mo timepoint* (+) Association for fore milk fat and WAZ	infant sex

Larsson et al. Denmark, 2018 (HIC) [47]	Cross-Sectional 59 infants enrolled 30 analyzed	5 mo fat, protein, lactose (calculated daily intake, concentrations) Exclusive BF only	5 mo BMIZ, WAZ, LAZ (WHO standards)	None	exclusive BF

Makela et al. Finland, 2013 (HIC) [68]	Longitudinal 100 infants enrolled 88 analyzed	3 mo fat, fatty acids (concentrations) Mixed Feeding	13 mo weight, length, BMI	*13 mo timepoint* (-) Association for unsaturated:saturated fatty acids and BMI (-) Association for monounsaturated:saturated fatty acids and BMI *Difference for birth and 13 mo* (+) Association for saturated fatty acids and weight gain (-) Association for unsaturated:saturated fatty acids and weight gain (-) Association for monounsaturated:saturated fatty acids ratio and weight gain (+) Association for saturated fatty acids and BMI gain (-) Association for unsaturated:saturated fatty acids and BMI gain (-) Association for monounsaturated:saturated fatty acids ratio and BMI gain	none reported

Martini et al. Indonesia, 2020 (LMIC) [48]	Longitudinal 40 infants enrolled 30 analyzed	1, 2, 3 mo fat, protein, lactose (concentrations) Exclusive BF only	1, 2, 3 mo weight, length, HC	*1 mo timepoint* (+) Association for protein and length (+) Association for protein and HC	none reported

Miliku Canada, 2019 (HIC) [17]	Longitudinal (subset of cohort) 1094 infants	3-4 mo fatty acids (concentrations) Mixed Feeding	3 mo, 1 y weight, length	*3 mo timepoint* (-) Association for palmitoleic acid and weight (-) Association for eicosadienoic acid and weight (-) Association for dihomo-gamma-linolenic acid and weight (-) Association for ARA and weight (-) Association for eicosatetraenoic acid and weight (-) Association for conjugated linoleic acid and weight (-) Association for EPA and weight (-) Association for docosapentaenoic-n3 acid and weight (-) Association for DHA and weight (-) Association for palmitoleic acid and length (-) Association for vacennic acid and length (-) Association for eicosadienoic acid and length (-) Association for eicosatetraenoic acid and length (-) Association for conjugated linoleic acid and length (-) Association for DHA and length *1 y timepoint* (-) Association for vacennic acid and length (-) Association for docosapentaenoic-n6 acid and length	unadjusted estimates provided by authors

Miller et al. United States, 2017 (HIC) [73]	Cross-sectional 63 infants	1–9 mo fat (fore, hind milk %) (concentrations) Mixed Feeding	1–9 mo WAZ, LAZ (WHO standards)	(-) Association for hind milk % fat and LAZ	infant sex, age maternal age, BMI, exclusive BF; nursing session duration, time since last session, time of day

Minato et al. Japan, 2019 (HIC) [43]	Longitudinal 129 infants enrolled 88 analyzed at 1 mo 56 analyzed at 3 mo	1, 3 mo fat, protein, carbohydrates (concentrations) Mixed Feeding	1, 3 mo weight (infants categorized by lower or normal weight gain)	*1 mo timepoint* (-) Association for protein and weight (+) Association for carbohydrates and weight	exclusive BF

Mitoulas et al. Australia, 2002 (HIC) [50]	Longitudinal 17 infants	1, 2, 4, 6, 9, 12 mo fat, protein, lactose (estimated intake, concentrations) Exclusive BF only	6 mo weight	None	exclusive BF

Much et al. Germany, 2013 (HIC) [75] Meyer et al Germany, 2019 (HIC) [76]	Randomized controlled trial 208 infants enrolled 152 analyzed at 6 wk 120 analyzed at 4 mo	6 wk, 4 mo fatty acids (concentrations)	6 wk, 4 mo, 1 y, 2 ys weight, BMI, length body composition (skinfold thickness, fat mass, fat mass %, subcutaneous/preperitoneal fat, ponderal index)	*6 wk timepoint (6 wk sample)* (-) Association for ARA:DHA and BMI (-) Association for n-6:n-3 LCPUFA and BMI (-) Association for n-6 LCPUFA and skinfold thickness (-) Association for ARA and fat mass (-) Association for n-6 LCPUFA and fat mass and fat mass % (+) Association for DHA and subcutaneous/preperitoneal fat (+) Association for n-3 LCPUFA and subcutaneous/preperitoneal fat (-) Association for n-6:n-3 LCPUFA and ponderal index *4 mo timepoint (6 wk sample)* (+) Association for DHA and skinfold thickness (+) Association for DHA and fat mass % *4 mo timepoint (4 mo sample)* (-) Association for EPA and length (-) Association for n-3 LCPUFA and length (-) Association for ARA:DHA and preperitoneal fat (-) Association for n-6:n-3 LCPUFA and preperitoneal fat *1 y timepoint (6 wk sample)* (+) Association for EPA and skinfold thickness (+) Association for n-3 LCPUFA and skinfold thickness *1 y timepoint (4 mo sample)* (-) Association for DHA and BMI (-) Association for DHA and length (-) Association for EPA and length (-) Association for n-3 LCPUFA and length (+) Association for DHA and ponderal index (+) Association for EPA and ponderal index (+) Association for n-3 LCPUFA and ponderal index *2 y timepoint (6 wk sample)* (+) Association for n-3 LCPUFA and weight (-) Association for n-6:n-3 LCPUFA and weight (+) Association for DHA and BMI (+) Association for n-3 LCPUFA and BMI (-) Association for n-6:n-3 LCPUFA and BMI (+) Association for n-3 LCPUFA and skinfold thickness (+) Association for DHA and fat mass % (+) Association for n-3 LCPUFA and fat mass %	infant sex, gestational age, ponderal index at birth; pregnancy duration, maternal parity, study group, exclusive BF

Mychaleckyj et al. Bangladesh, 2020 (LMIC) [83]	Longitudinal 700 infants enrolled 563 analyzed	3–43 d fatty acids (concentrations) Mixed Feeding	6 wk, 1 y, 2 y WAZ, LAZ (WHO standards)	*Difference for 6 wk and 1 y* (+) Association for gamma-linolenic acid and LAZ increase *Difference for 6 wk and 2 y* (+) Association for gamma-linolenic acid and LAZ increase	infant serum zinc, sex, age, gestational age, HM AA and DHA, log(%AA) and log(%DHA)

Nikniaz Jr. et al. Iran, 2009 (LMIC) [87]	Cross-sectional 182 infants	3–4 mo fat (concentrations) Mixed Feeding	3–4 mo WAZ (NCHS standards)	(+) Association for fat and WAZ	infant birth weight maternal BMI, age energy intake

Nuss et al. United States, 2019 (HIC) [40]	Cross-sectional 33 infants	4-8 wk fatty acids: n-6, n-3 PUFA (concentrations)	4-8 wk weight, length, HC body composition (fat mass % using air-displacement plethysmography [PEAPOD, COSMED, Concord, CA])	(-) Association for n-6 PUFA and weight (-) Association for n-3 PUFA and weight (-) Association for n-6:n-3 PUFA and weight (-) Association for n-6 PUFA and HC (+) Association for n-3 PUFA and HC (-) Association for n-6:n-3 PUFA and HC (-) Association for n-6 PUFA and fat mass % (+) Association for n-3 PUFA and % fat mass (-) Association for n-6:n-3 PUFA and fat mass %	infant age

Palmer et al. Zambia, 2016 (LMIC) [69]	Randomized controlled trial 149 infants enrolled 145 analyzed	4–12 mo fat (concentrations)	4–12 mo weight, length	None	unadjusted estimates provided by authors

Peng et al. 2021, China (UMIC) [77]	Longitudinal 101 infants	1, 2, 3 mo fatty acids (concentrations) Exclusive BF only	1, 2, 3 mo weight, length, BMI, HC	(-) Association for (SFA) C18:0 (2 mo) and HC at 2 mo (-) Association for (SFA) C18:0 (3 mo) and HC at 3 mo (-) Association for (PUFA, n-3 profile) C20:3n3 (3 mo) and HC at 3 mo (-) Association for (PUFA, n-3 profile) C20:5n3 (3 mo) and HC at 3 mo No other associations reported.	none reported

Prentice et al. United Kingdom, 2016 (HIC) [49] Prentice et al. United Kingdom, 2019 (HIC) [53]	Longitudinal (subset of cohort) 619 infants	4–8 wk fat, fatty acids (butyrate, formate, acetate), protein, lactose (concentrations) Mixed Feeding	3, 12, 24 mo weight, length, BMI body composition (skinfold thickness)	*3 mo timepoint* (-) Association for formate and BMI (-) Association for acetate and skinfold thickness *1 y timepoint* (-) Association for fat and BMI (-) Association for butyrate and BMI (-) Association for formate and BMI (+) Association for lactose and BMI (-) Association for fat and skinfold thickness (-) Association for butyrate and skinfold thickness (+) Association for lactose and skinfold thickness *Difference for 3 mo and 1 y* (-) Association for fat and weight increase (-) Association for butyrate and weight increase (+) Association for lactose and weight increase (-) Association for fat and BMI increase (-) Association for butyrate and BMI increase (+) Association for lactose and BMI increase (-) Association for fat and skinfold thickness increase (+) Association for lactose and skinfold thickness increase *2 y timepoint* (-) Association for formate and weight (-) Association for formate and BMI (-) Association for formate and skinfold thickness (-) Association for acetate and skinfold thickness *Difference for 1 and 2 y* (+) Association for butyrate and weight increase (+) Association for butyrate and BMI increase (+) Association for butyrate and skinfold thickness increase	infant sex, birthweight, gestational age; exclusive BF, duration of sample storage

Riederer et al. 2020 Austria (HIC) [41]	Cross-Sectional 54 infants (47 analyzed)	6–8 wk amino acids, onylipins (concentrations) Exclusive BF only	14–16 wk length. weight, body composition (FM, FFM), FMI< FFMI, BMI using air-displacement plethysmography {PEAPOD VR, COSMED, Rome, Italy]) (WHO standards)	(No) Associations reported for HM AA and anthropometry (+) Association for HM oxylipins 11-HETE and 13-HDHA together and FMI (-) Association for HM oxylipin 17-HDHA and FFMI	BMIZ, gestational weight gain, exclusive BF

Rudolph et al. United States, 2017 (HIC) [88]	Longitudinal 48 infants	2 wk, 4 mo fatty acids (AA:(DHA+EPA) ratio) (Ratios in HM) Exclusive or predominant BF	2 wk, 4 mo weight, body composition (fat mass, fat free mass, fat mass %)	*4 mo timepoint* (+) Association for ARA:(DHA+EPA) ratio and fat mass *Difference for 2 wk and 4 mo (4 mo sample)* (+) Association for ARA:(DHA+EPA) ratio and change in fat mass (+) Association for ARA:(DHA+EPA) ratio and change in fat mass %	infant sex, birth weight, gestational weight gain category; maternal BMI, fish oil supplements; exclusive BF

Saben et al. 2022, USA (HIC) [59]	Longitudinal (2 cohorts) 194 infants (normal weight, *n =* 68; OW, *n =* 51; OB, *n =* 75)	0.5, 2, 6 mo amino acids (concentrations) Mixed Feeding	0.5, 2, 6 mo length, weight, weight for GA (WHO standards)	*0.5 - 6 mo* (No) Association for free amino acid intake and WAZ (+) Association for Asp, C-C, Glu and His and WLZ (+) Association for Asp, C-C, Gln, Glu, His and Ser and FMI (+) Association for Asp, C-C, Gln, Glu, His, and Ser and FFMI	infant sex, body composition, birth weight

Scholtens et al. The Netherlands, 2009 (HIC) [79]	Longitudinal (subset of cohort) 244 infants enrolled 177 analyzed	3-4 mo fatty acids (infants categorized by fatty acid tertiles) (concentrations)	1 y weight, BMI, length	*Difference for birth and 1 y* (-) Association for linoleic acid and weight gain (-) Association for n-6 PUFA and weight gain (-) Association for ARA and BMI gain (-) Association for linoleic acid and BMI gain (high tertial only) (-) Association for n-6 PUFA and BMI gain (high tertial only)	infant age BF duration low and high fatty acid tertials Sample collection time

Sims et al. United States, 2020 (HIC) [33]	Longitudinal 284 infants enrolled 174 analyzed	2 wk, monthly between 1, 6 mo, 9 mo fat, protein, carbohydrates (estimated intake) Mixed Feeding	2 wk, monthly between 1, 6 mo, 9 mo WLZ, WAZ, LAZ body composition (fat mass index, fat free mass index using quantitative NMR [Echo MRI-AH; Echo Medical Systems]) (WHO standards)	*All timepoints up to 9 mo (linear mixed effects model accounting for time)* (+) Association for carbohydrate intake and WLZ (-) Association for fat intake and WAZ (+) Association for protein intake and WAZ (-) Association for carbohydrate intake and WAZ (+) Association for protein intake and LAZ (-) Association for carbohydrate intake and LAZ (-) Association for protein intake and fat mass index (+) Association for carbohydrate intake and fat mass index (-) Association for protein intake and fat free mass index (-) Association for carbohydrate intake and fat free mass index	infant sex, exclusive BF

Tyson et al. United States, 1992 (HIC) [70]	Longitudinal 40 infants	2, 6 wk fat yield (mothers categorized by low or high fat yield index) (concentrations)	2, 6 wk weight, length, HC body composition (skinfold thickness)	*Difference for birth and 6 wk* (+) Association for fat yield and weight gain *Difference for 2 and 6 wk* (+) Association for fat yield and skinfold thickness increase	none reported

Ulloa et al. 2020, Argentina, (HIC) [54]	Longitudinal 36 infants (*n =* 13, EWG; *n =* 23, AWG)	Protocol entry 4.34 (2.07–5.93) mo. fat, protein, carbohydrate (concentrations) Exclusive BF only	Protocol entry, monthly thereafter until 1 y weight, length, WAZ, LAZ, WLZ (WHO standards)	(No) Association for protein and excessive weight gain (No) Association for fat and excessive weight gain (No) Association for carbohydrates and excessive weight gain.	exclusive BF

Urteaga et al. Bolivia, 2018 (LMIC) [39]	Cross-sectional 18 infants	2–6 mo fat (concentrations) Exclusive BF only	2–6 mo WLZ, BMIZ, WAZ, LAZ body composition (fat mass, fat mass % using dual Energy X-ray Absorptiometry: DEXA) (WHO standards)	None	exclusive BF

van Sadelhoff et al.The Netherlands, 2018 (HIC) [89]	RCT 25 infants enrolled 16 analyzed	monthly between 1, 6 mo amino acids (concentrations)	Every 2 mo between birth, 6 mo weight, length	None	infant sex

van Sadelhoff et al. 2021, Germany (HIC) [60]	Longitudinal 741 infants (441 analyzed)	6 wk, 6 mo amino acids (concentrations) Mixed Feeding	6 wk, 6 mo weight, length, weight gain, length gain.	(-) Association for Threonine, Glutamate, glutamine and serine and weight gain at 6 wk (-) Association for Glutamine and length gain at 6 wk (-) Association for all FAAs (free AAs) and weight gain at 6 wk	none reported

Xiang et al.China, 1999(LMIC) [81]	Cross-sectional 41 infants (18 infants 1 mo old, 23 infants 3 mo old)	1 or 3 mo fatty acids: ARA, DHA (concentrations) Exclusive BF only	1 or 3 mo weight, length	*Difference for birth and 1 m* (+) Association for DHA and length gain*Difference for birth and 3 mo* (+) Association for ARA and weight gain (+) Association for DHA and weight gain (+) Association for DHA and length gain	none reported

Xiang et al.Sweden, 2000(HIC) [80]	Longitudinal 19 infants	1, 3 mo fatty acids (concentrations) Exclusive BF only	1, 3 mo Occipito-frontal HC Brain weight	*Difference for birth and 1 mo* (+) Association for ARA:DHA and occipito-frontal HC increase (+) Association for ARA:DHA and brain weight increase *Difference for birth and 3 mo* *(*+) Association for ARA:DHA and occipito-frontal HC increase (+) Association for ARA:DHA and brain weight increase	none reported

Zhang et al. 2021, China (LMIC) [55]	Longitudinal 105 infants	8–14 d, 1 mo, 6 mo protein, alpha-lactalbumin (concentrations)	8–14 d, 1 mo, 6 mo weight, length, LFA, WA, WFL (WHO standards)	(-) Associations for Alpha-casein and WFA z-scores (No) Association for other proteins and anthropometry	infant age, sex; maternal age, education, household income, pre-gestational BMI, mode of delivery, parity

∗Indicates data were provided by the study author and do not appear in referenced publication.

Abbreviations: ARA, arachidonic acid; BF, breastfeeding; HAZ, height for age z-score; HC, head circumference; HCAZ, head circumference z-score; HIC, high income countries; HM, human milk; LAZ, length for age Z-score; LCPUFA, long chain polyunsaturated fatty acids; LFA, length for age; LMIC, low- and middle-income countries; NCHS, National Center for Health Statistics; RCT, randomized controlled trial; SCM, subclinical mastitis; WAZ, weight for age z-score; WFA, weight for age; WLZ, weight-for-length z-score.

∗∗ "No (assumed) associations = unreported associations assumed to be no association.

The primary outcomes of interest were indicators of growth and body composition in infants, including weight-for-age or weight-for-age z-score (WAZ), length-for-age or length-for-age z-score (LAZ), weight-for-length or weight-for-length z-score (WLZ), BMI or BMI-for-age z-score, and growth velocity. Different studies used different reference populations to calculate these z-scores (e.g., WHO or National Centre for Health Statistics), and some studies presented results in percentiles instead of z-scores. Furthermore, we considered other infant anthropometric measurements from the articles, such as weight, length, rapid weight gain (as reported by the authors), total adiposity (% fat measured by DXA or skinfold thickness), body composition (fat mass [FM], fat free mass [FFM], %Fat mass measured by bioelectrical impedance spectroscopy or skinfold thickness), stunting, wasting, under- or overweight, and head circumference.

### Quality Assessment

Articles were assessed for quality using a modified Newcastle-Ottawa scale (Supplemental Table 1). Using a 17-point evaluation scale, we designated 8 points for HM exposure assessment, 5 points for maternal and infant confounders considered, and 4 points for infant anthropometry outcome assessment. Quality assessments for each article were conducted in duplicate, with conflicts addressed through consensus. Overall quality scores between >13 and 17 were considered high; 7 to 13 moderate; and < 7—low. Quality scores were also evaluated individually for exposure assessment (high: >6–8, moderate: 3–6, low: <3), confounders considered (high: >4–5, moderate: 3–4; low: <3), and outcome assessment (high: >3–4, moderate: 2–3, low: <2) (Supplemental Table 2).

### Data Extraction

Data extraction was conducted using a standardized form that was developed and piloted in collaboration with subject matter experts. Study authors were contacted to request data in instances where data were missing or presented in non-extractable formats. Each article was extracted in duplicate, and conflicts were addressed through consensus.

### Analytical Strategies

Associations between HM macronutrients and infant growth outcomes were reported using effect direction heatmaps when associations were reported in ≥ 30 articles [30]. Directional associations reported for HM concentrations were visualized in heat maps. Studies that reported only estimated daily intakes (rather than concentrations) were described narratively. Color gradients were determined by assigning a score to each outcome (+1 for positive associations, -1 for inverse associations, and 0 for no/assumed no association). These scores were summed and then divided by the total number of studies reporting for each outcome. When articles only reported statistically significant outcomes, unreported associations were considered as “assumed no association” and assigned a value of 0 (i.e., “no association”). If the direction of effect was discordant across studies, the associations were presented as a gradient of color based on the number of studies reporting associations and the mean direction of association among studies. If multiple timepoints were reported for a growth outcome, the earliest timepoint reported was extracted for the heatmap.

Results were summarized narratively according to the SWiM reporting guidelines and included general result trends across all applicable studies [30]. Macronutrients were grouped into 3 categories based on their chemical structures: fats, digestible carbohydrates, and proteins. Fats included total fat and FAs, carbohydrates consisted of total carbohydrates, lactose, fructose, and glucose, and proteins included total protein and amino acids.

## Results

### Description of Included Studies

**FIGURE 1:**
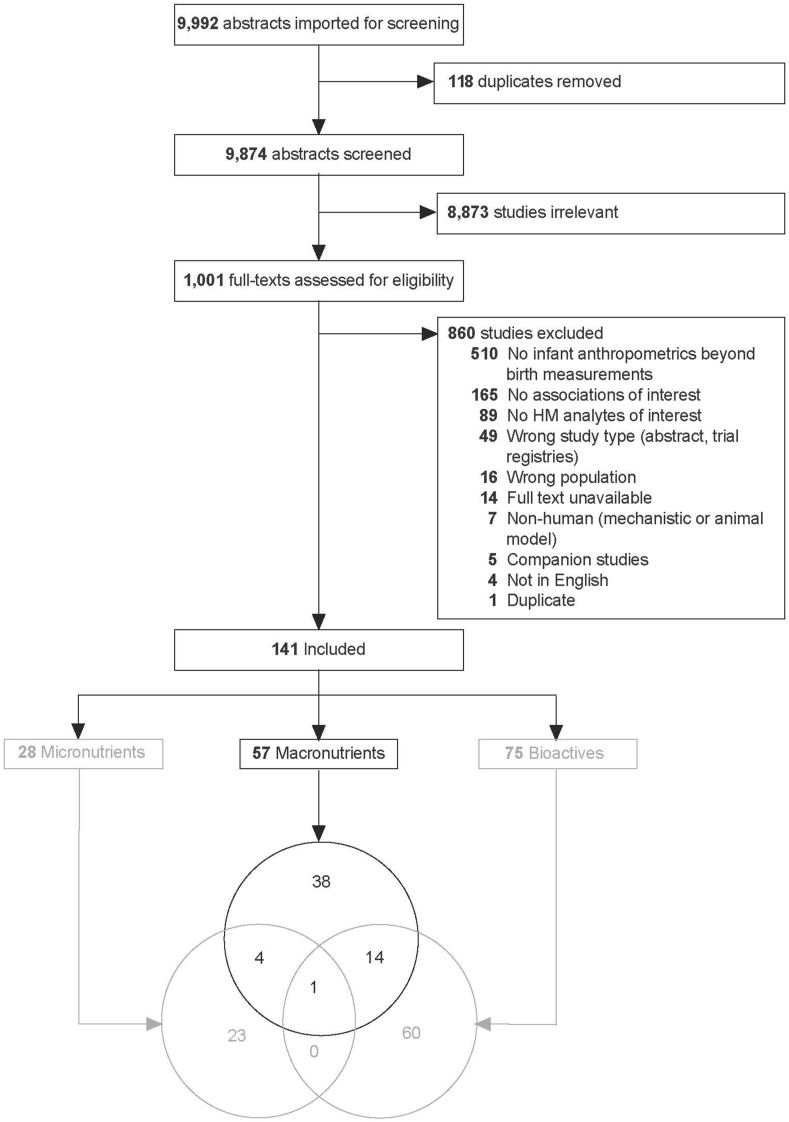
Systematic review of associations between human milk macronutrients and infant growth in the first 2 y: PRISMA flow diagram. Irrelevant articles did not meet inclusion criteria, such as ill or preterm infants or articles that only examine formula intake. Reasons for study exclusion were recorded in the order listed in the figure. Though some studies had more than one reason for exclusion, each study was only counted once (e.g., if a study reported no human milk analytes of interest and was not in English, it was recorded as the former). Macronutrient studies are reported in the current paper; Micronutrient and Bioactive studies are reported separately [5,108].

In total, 9,992 abstracts were identified, and 937 full texts were screened (Figure 1). The main reasons for excluding articles were: no HM analytes of interest reported (*n* = 89); no infant anthropometrics or only birth anthropometrics were reported (*n* = 510); or no associations between HM analytes and infant anthropometrics were reported (*n* = 165). Together, these 3 reasons accounted for 90% (731/815) of the studies excluded at the full-text screening stage. Notably, the latter 153 studies could have potentially contributed to the literature as they reported values of milk analytes and infant anthropometrics but did not report their associations.

Data were extracted from 141 articles reporting associations between HM components and infant anthropometrics, of which 57 articles (representing 53 studies and 5979 dyads) reported on HM macronutrients and are included here. Associations between HM micronutrients and bioactives are reported in separate manuscripts [5,32]. Table 1 summarizes the findings from included studies. In total, 38 articles (36 studies) examined fat and/or FA, 23 articles (20 studies) examined proteins and/or amino acids, and 16 articles (13 studies) examined carbohydrates.

Among the 57 included macronutrient articles, 42 (72%) were published in 2010 or later, and 7 (12%) were published prior to 2000. Fifteen studies were conducted in lower middle-income countries (LMIC, according to World Bank criteria [90]), in upper-middle income settings, and 35 studies were conducted in high income settings. Most studies were longitudinal (42/53; 72%), reporting outcomes at 2 or more time points.

Milk collection strategies and time points varied considerably across studies. Twenty-three articles reported analyte concentrations in milk from a single collection timepoint. Only 10 articles reported calculated daily milk intakes, incorporating milk volume data, and many of these were from the same research group [33,34,91,92]. Milk sampling times varied from birth (colostrum) to 14 mo, with the most common timepoints for milk sampling being 1 mo (26 articles) and 3 mo (18 articles).

### Study quality

**FIGURE 2:**
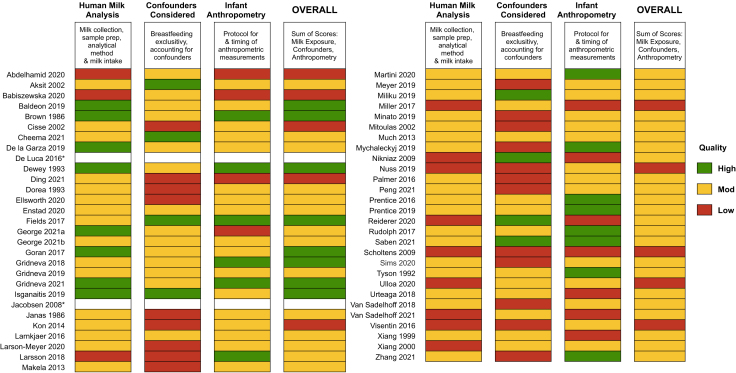
Summary of quality assessments of included articles. Association of human milk bioactives and infant growth in the first 2 y Quality scores awarded based on the number of points assigned according to criteria in Supplemental Table S1. Detailed numeric scores are presented in Supplemental Table S2. HM, human milk. ∗Indicates that data were collected directly from authors and no quality assessment of an article was conducted.

Most articles [33] were rated as moderate overall quality (8.5 - 12.75 score on the modified Newcastle-Ottawa scale; maximum 17 points), with 11 studies being rated as low quality (<8) and 11 studies rated as high quality (>13) (Figure 2). A detailed breakdown of individual study quality scores is presented in Supplemental Table 2. The most common quality issue across studies was failing to adjust for confounders, such as breastfeeding practices (e.g., exclusivity, direct breastfeeding), maternal body mass index (BMI), or maternal age. Additionally, inconsistent timing of milk sampling (milk collection over a period of time [e.g., 6–14 mo] rather than at a single time point [e.g., 6 mo]) and sample strategies were reported as quality concerns (Supplemental Table 2). Macronutrient consumption is dependent on both the concentration in HM and the total volume of HM consumed throughout the day. While many studies report HM macronutrient concentrations, studies that additionally reported daily intake of milk were consistently scored as higher quality.

### Infant Anthropometrics

There was considerable variety in the anthropometry outcomes reported in each study (Table 1). Studies that reported standardized z-scores used either US National Centre for Health Statistics (NCHS) standards or WHO standards, except for de Fluiter et al. (2021) [35], who used an online growth analyzer. Of studies reporting body composition, 5 studies used X-ray technology such as DEXA [9,[36], [37], [38], [39]], and 3 studies used air-displacement technology such as PEAPOD [35,40,41]. Other technologies to determine body composition included bioimpedance (37) and magnetic resonance imaging [33].

### Carbohydrates

**FIGURE 3:**
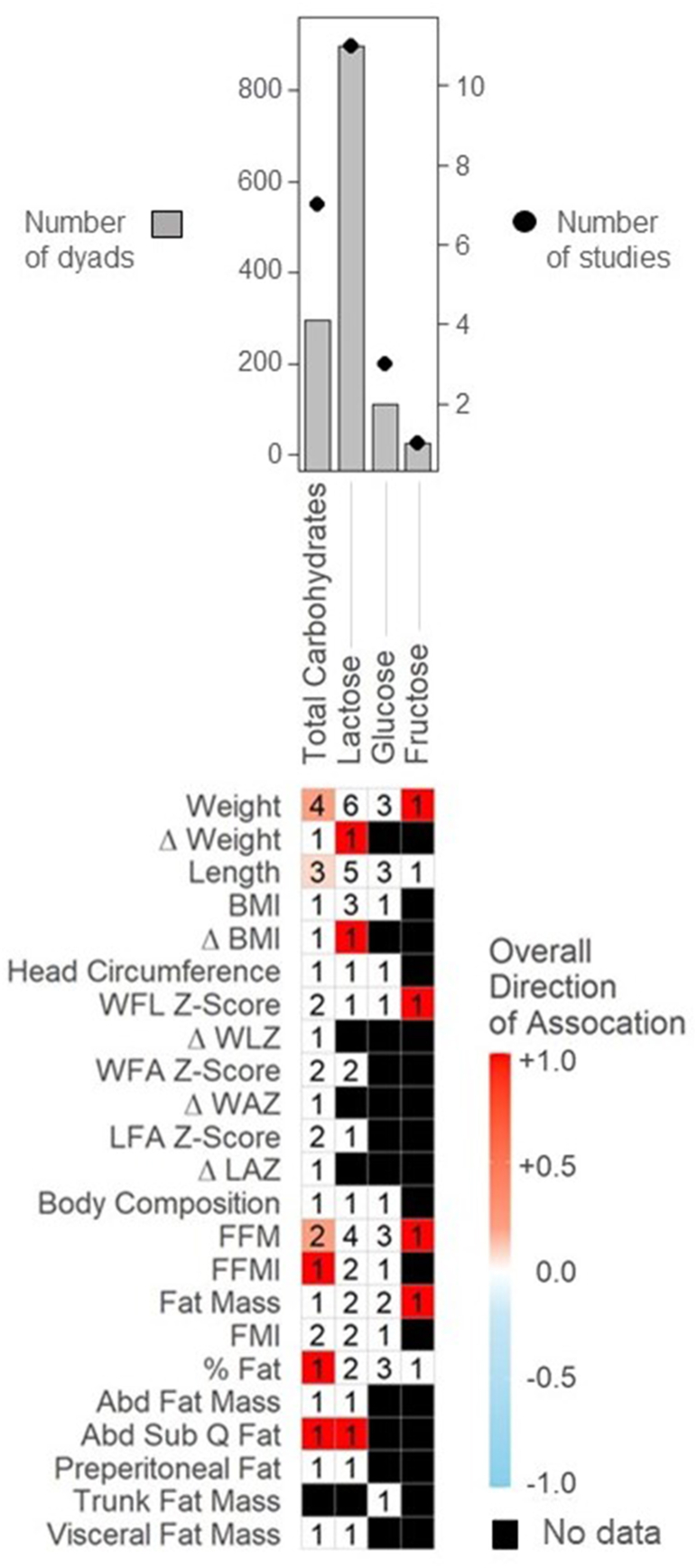
Mean directions of associations between Concentrations of Human Milk Carbohydrates and infant growth in the first 2 y. Significant associations between carbohydrates and infant anthropometrics reflect results as reported by individual study authors (e.g., using human milk concentrations as the predictor variable, see Table 1). Value in cells indicate the number of studies examining each comparison. Red squares indicate mean positive associations, blue squares indicate mean inverse associations, white squares indicate a mean association of 0, and black squares indicate that association was not assessed. Abbreviations: Δ Weight – weight gain; Δ BMI – BMI gain; WFL - weight for length; Δ WLZ – gain in weight-for-length Z-Score; WFA – weight for age; Δ WAZ – gain in weight-for-age Z-Score; LFA - length for age; Δ LAZ – gain in length-for-age Z-Score; FFM - fat free mass; FFMI – fat free mass index; FMI - fat mass index; ABD – abdominal; Sub Q - subcutaneous.

Five studies (7 articles) involving 295 dyads examined the relationship between total carbohydrates and infant growth outcomes (Figure 3, Supplemental Table 3). Two of 4 studies observed positive associations for infant weight [42,43]. Gridneva et al. (2019) [42] observed this association at 2, 5, 9, and 12 mo of age, whereas Minato et al. (2019) [43] observed this association at 1 mo of age, Gridneva et al. (2019) [42] also observed positive associations between total carbohydrates concentration and infant length, FFM index, FFM, percent fat, and abdominal subcutaneous fat [44] at 2, 5, 9 and 12 mo of age. Notably, Gridneva et al. (2019, 2018, 2022) [42,45,93] was the only study to report both total carbohydrates and lactose concentrations (reported below). This is important because total carbohydrate measures are not indicative of digestible carbohydrates and include other nondigestible carbohydrates, such as HMOs [94].

Five of the 13 studies exploring carbohydrate components in HM used the midinfrared MIRIS human milk analyzer, commercially available since 2006 (MIRIS AB, Uppsala, Sweden) [35,43,[46], [47], [48]]. Two studies used an enzymatic assay and UV spectrometry [34,42], and the other 6 each used a different assay to analyze HM carbohydrates, such as nuclear magnetic resonance spectrometry [49] (Supplemental Table 1). Whereas some studies demonstrated that HM lactose analysis using current MIRIS technology is comparable with the gold standard method of HPLC [95], others show that there is large variation in the reproducibility of readings [96]. As such, results from these studies should be considered carefully.

Lactose was the most extensively explored carbohydrate, reported in 8 studies (11 articles) involving 898 dyads. Whereas no associations were observed between lactose and weight (6 studies) or length (5 studies), a positive association was observed between HM lactose concentration and changes in BMI and infant weight from 3 mo to 1 y of age (Figure 3). However, this association was only examined in 1 study [49]. Abdominal subcutaneous fat was also positively associated with concentrations of HM lactose throughout the first year of life; however, again, this association was only examined by 1 study [44].

None of the 3 studies (112 dyads) examining glucose and infant growth observed any associations [9,34,37]. Fructose was only examined in 1 study (25 dyads) [9], which demonstrated positive associations between HM fructose levels and infant weight and mass at 6 mo of age: in models adjusting for infant weight, sex and maternal prepregnancy BMI, Goran et al. (2017) [7] estimated that for every 1μg/ml increase in fructose, there was a 257g increase in weight (*P* = 0.02), 170g increase in lean mass (*P* = 0.01), and 131g increase in fat mass (*P* = 0.05).

#### Calculated daily intakes (CDI)

Five studies examined the relationship between CDI of HM carbohydrates and infant growth [33,34,42,47,50]. Neither Gridneva et al. (2019) [42], Larsson et al. (2018) [47], nor Mitoulas et al. (2020) [50] found any associations between CDI of lactose or total carbohydrates and infant growth in their longitudinal studies. However, in a cross-sectional sectional study, Cheema et al. (2021) [34] found positive associations between CDI of lactose and infant weight, length, WAZ, fat free mass index, and fat free mass at 3 mo of age. In contrast, Sims et al. (2020) [33] reported observations for CDI of total carbohydrates and found inverse associations between total carbohydrates and weight-for-age (WA) and LAZ as well as fat free mass from 2 wk to 9 mo of age. Sims et al. (2020 [33]) also observed positive associations between CDI for total carbohydrates and WLZ and fat mass index assessed monthly from 1 to 6 mo. However, these associations were not observed for HM concentrations of lactose in other studies [34,42,47,50]. Finally, Cheema et al. (2021) [34] observed a positive association between CDI of HM glucose and infant head circumference at 3 mo of age, an association that was not examined in any other studies.

### Proteins and Amino Acids

#### Proteins

**FIGURE 4:**
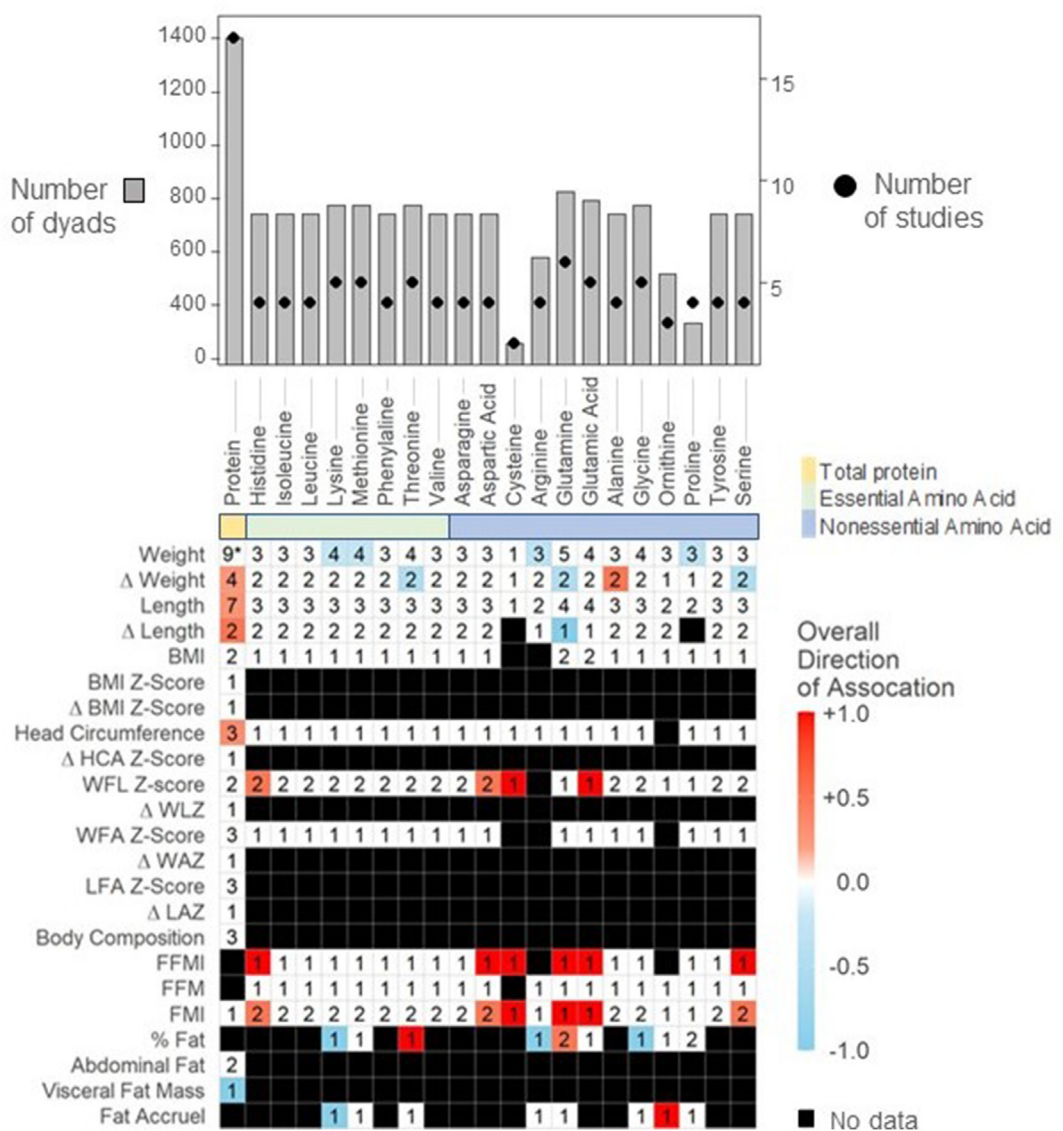
Mean directions of associations between Concentrations of Human Milk Protein and Amino Acids and infant growth in the first 2 y. Significant associations between proteins and infant anthropometrics reflect results as reported by individual study authors (e.g., using human milk concentrations as the predictor variable, see Table 1). Value in cells indicate the number of studies examining each comparison. Red squares indicate mean positive associations, blue squares indicate mean inverse associations, white squares indicate a mean association of 0, and black squares indicate that association was not assessed. ∗Indicates that equal numbers of positive and negative associations were observed, resulting in a gradient of zero (0). Abbreviations: Δ Weight – weight gain; Δ Length – length gain; Δ BMI Z-Score – gain in BMI Z-Score; Δ HCA Z-Score – gain in head circumference-for-age Z-Score; WFL - weight for length; Δ WLZ – gain in weight-for-length Z-Score; WFA - Weight for age; Δ WAZ – gain in weight-for-age Z-Score; LFA - length for age; Δ LAZ – gain in length-for-age Z-Score; FFMI – fat free mass index; FFM - fat free mass; FMI - fat mass index.

Seventeen articles, including 14 studies involving 1403 dyads, examined the associations between total HM protein and infant growth outcomes (Figure 4, Supplemental Table 4). Crude protein values were analyzed in 7 studies [45,[50], [51], [52], [53], [54]], with only de Fluiter et al. (2021) [35] analyzing crude and true protein levels separately. Whereas 8 of 14 studies used various assays such as the Bradford [91,55] and Kjeldahl methods [[50], [51], [52]], 6 studies assessed protein content using the commercialized MIRIS human milk analyzer [35,43,[46], [47], [48],^56^], which provides both crude and true protein values [23,95]. However, only de Fluiter et al. (2021) [35] specified if they were using crude or true protein in their analysis. It is assumed that the other 5 studies used true protein values as these are more representative of digestible proteins for the infant [94], but this was not explicitly stated. Notably, it has been reported that human milk analyzers overestimate total protein by approximately 15% compared with the Kjeldahl (gold standard) method [95].

Although few associations were assessed by more than 1 study, protein demonstrated positive associations with multiple growth measures, including length at 1 mo [56], length gain from birth to 1 mo [63], weight gain from birth to 6 mo [57] and head circumference at 1 mo [48] whereas an inverse association was observed for visceral fat mass [35]. Contradictory findings were reported for HM protein and infant weight at 1 mo of age, with De Luca [56] observing a positive association and Minato [43] observing an inverse association. However, the data from De Luca et al. (2016) [56] study were unadjusted estimates that were provided to us directly by the authors as opposed to being extracted from the published study. The lack of adjustment may account for the difference in findings.

#### Amino Acids

Five studies examined the association between essential amino acids and infant growth, with 3 studies assessing free amino acids [41,58,59], 1 examining both free and total amino acids [60], and 1 conducting untargeted metabolomics [38]. Two studies used ion-exchange chromatography [41,58], whereas Saben et al. (2022) [59] and van Sadelhoff (2021) [60] used liquid chromatography to analyze amino acids. Isganaitis et al. (2019) [38] used untargeted metabolomics to detect amino acids in HM. Of the essential amino acids, only histidine, lysine, methionine, and threonine had any associations with infant growth, but these associations were only observed in one study each. One study demonstrated positive associations between histidine and WLZ, FFM index, and fat mass index [59]. Lysine and methionine were inversely associated with infant weight at 1 mo of age, these associations were only observed in one study [38]. Threonine demonstrated a positive association with percent fat mass at 1 mo [38] and an inverse association with infant weight gain [60].

Non-essential amino acids were examined by the same 5 studies as essential amino acids with the addition of Larnkjaer et al. (2016) [61]. No consistent associations were observed between non-essential amino acids and infant anthropometry. Only one study demonstrated inverse associations between arginine and proline and infant weight at 1 mo of age [38]. Whereas glutamine had mixed results with infant growth, with inverse associations observed for weight gain and length gain from birth to 6 wk [89] and positive associations observed for FFM index, fat mass index [59] and percent fat mass at 1 mo [38]. Inverse associations were also observed for serine and infant weight gain from birth to 6 wk [60]. Aspartic acid, cysteine [59], glutamic acid [58,59], and alanine [58] all had positive associations with infant growth in the first 4 to 6 mo of life.

#### Calculated daily intakes (CDI)

Six studies examined CDI of protein and infant growth outcomes [33,45,47,50,62,63]. Neither Larrson et al. (2018) [47], Mitoulas et al. (2002) [50], nor Gridenva et al. (2022) [45] found associations between CDI of HM protein and infant growth. Brown et al. (1986) [62] observed inverse associations between CDI of HM protein for both weight-for-age and WLZ in infants less than 3 mo, both of which conflicted with results reported by Sims et al. (2020) [33] and Cisse et al. (2002) [63] respectively. Cisse et al. (2002) [63] and Sims et al. (2020) [33] both observed positive associations between CDI of HM protein and LAZ at 3 mo and up to 9 mo of age, respectively. Cisse et al. (2002) [63] also observed positive associations for weight at 3 mo, whereas Sims et al. (2020) [33] observed inverse associations for fat mass index and FFM in infants up to 9 mo of age. Janas et al. (1987) [64] reported outcomes for CDI and amino acids but did not observe any statistically meaningful associations with infant growth.

### Fat and fatty acid content

#### Fat

**FIGURE 5:**
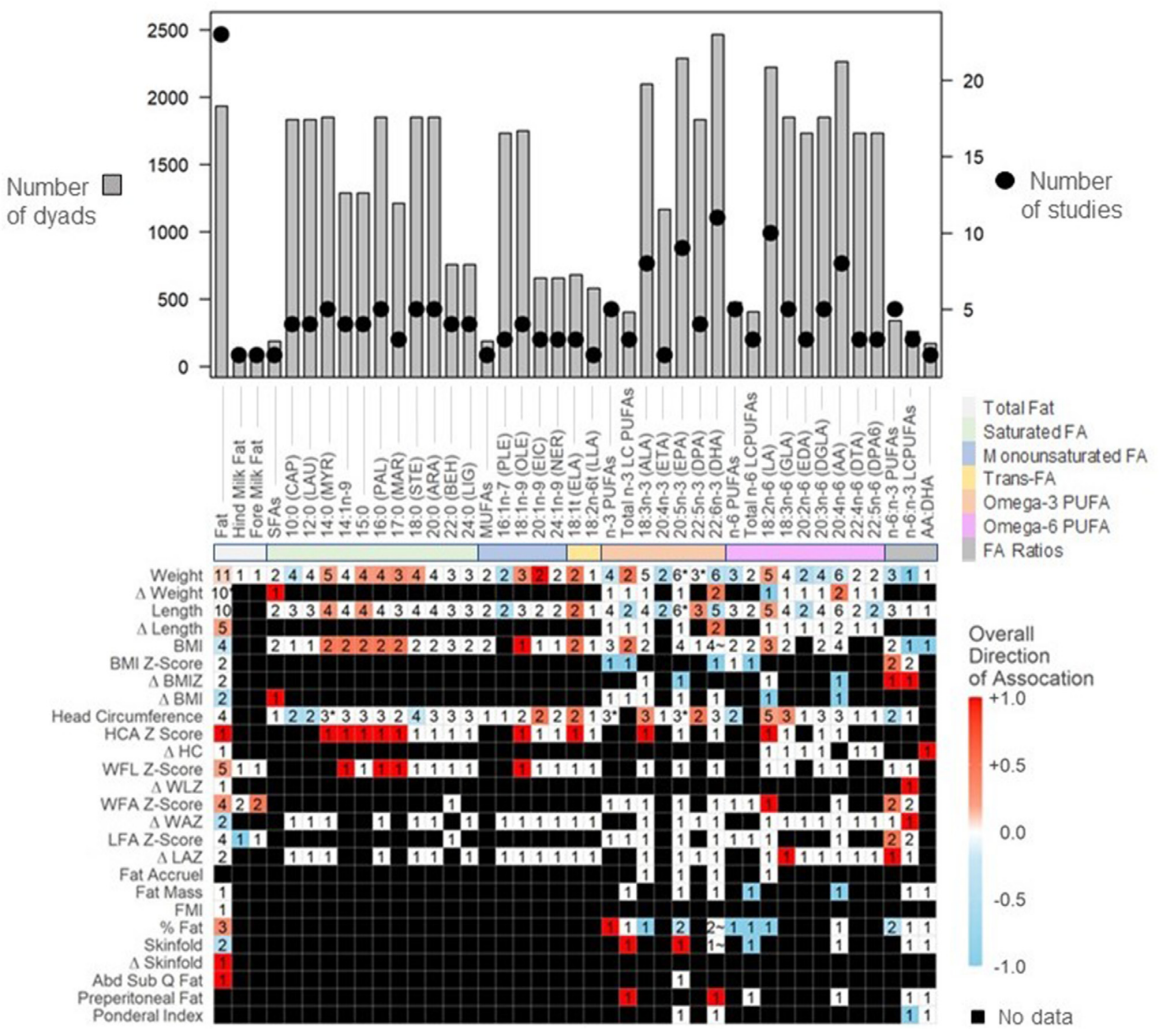
Mean directions of associations between Concentrations of Human Milk Fat and Fatty Acids and infant growth in the first 2 y. Significant associations between immunomodulators and infant anthropometrics reflect results as reported by individual study authors (e.g., using human milk concentrations as the predictor variable, see Table 1). Value in cells indicate the number of studies examining each comparison. Red squares indicate mean positive associations, blue squares indicate mean inverse associations, white squares indicate a mean association of 0, and black squares indicate that association was not assessed. ∗Indicates that equal numbers of positive and negative associations were observed, resulting in a gradient of zero (0). ∼ Indicates contradictory results within the same study at different time points. Abbreviations: Δ Weight – weight gain; Δ Length – length gain; Δ BMI – gain in BMI; HCA Z-Score – gain in head circumference-for-age Z-Score; Δ HC – gain in head circumference; WFL - weight for length; Δ WLZ – gain in weight-for-length Z-Score; WFA - Weight for age; Δ WAZ – gain in weight-for-age Z-Score; LFA - length for age; Δ LAZ – gain in length-for-age Z-Score; FMI - fat mass index; Δ Skinfold – gain in skinfold; Abd Sub Q – abdominal subcutaneous.

Twenty-three articles (21 studies) examined the association between HM fat and infant growth outcomes (Figure 5, Supplemental Table 5). Solvent extraction and creamatocrit (percentage of cream using gravimetry) are the most widely used methods to assess total fat content in HM [97]. In this review, creamatocrit was the most common method (7 studies; [39,[65], [66], [67], [68], [69], [70]], although 2 studies [67,68] additionally quantified fatty acid methyl esters, which can provide a good estimate of total fat content in HM [97]). Only Dewey et al. (1993) [71] used the modified Folch method to assess total fat. Six studies used the MIRIS human milk analyzer, all published after 2016 and primarily in high income settings with the exception of Martini et al. (2020) [48]. Notably, HM fat analysis using human milk analyzer demonstrates significantly different findings compared to the Roese-Gottlieb (gold standard) method [95], which should be considered when interpreting results from these instruments.

The majority of studies did not find any significant associations between HM fat and infant growth. Infant weight was examined in 11 studies, and only 1 study [67] found a positive association with total HM lipids for infants up to 6 mo of age. Four studies assessed infant BMI and HM fat, with only one [51] finding an inverse association, but this was from milk sampled across 6 to14 mo of lactation. Conflicting results were found for associations between HM fat and weight gain across 10 studies, with just 2 associations found in opposite directions at 1 mo [53] compared to 2 mo [65] of age. Ten studies looked at associations between HM fat and length, and none reported significant associations. Five studies examined WLZ scores in relation to HM; only one [67] found a positive association when examining daily fat intake and infant growth in infants up to 6 mo of age, and this was no longer observed when calculating a monthly average of fat intake.

Two studies [72,73] analyzed hindmilk fat and foremilk fat concentration separately. In their exploratory cross-sectional study, Miller et al. (2017) [73] noted an inverse association between hindmilk fat concentration and infant length-for-age in milk sampled across 1 to 9 mo of lactation (but no associations with infant weight), whereas Larson-Meyer et al. (2021) [72] found no associations between hind milk fat concentrations and infant growth at 1 mo of age. However, Larson-Meyer et al. (2021) [72] did find a positive association between foremilk fat concentration and infant weight-for-length scores, but only until 1 mo of age. Using a different strategy to address the variation in HM fat within a feed, George et al. (2021) [67] sampled milk using pre and postfeed samples and used CDIs to examine relationships between FAs and infant growth. It appeared that using George et al.'s (2021) [67] method yielded more significant associations between HM FAs in infants up to 6 mo of age compared with the single sample methods.

#### Fatty acids

Eighteen articles (17 studies) examined associations between 33 individual FAs and 13 FA groups with infant growth outcomes (Figure 5, Supplemental Table 3). All studies used chromatography to detect FAs in human milk, except for Prentice et al. (2019) [53] who used nuclear magnetic resonance and Isganaitis et al., (2019) [38] who used untargeted metabolomics. For the current study, FAs were divided into 6 groups: saturated fatty acids (SFAs), monounsaturated fatty acids (MUFAs), trans-fatty acids (TFAs), omega-3 PUFA (Omega-3 PUFAs), omega-6 PUFA (omega-6 PUFAs), and fatty acid ratios. All studies reported FAs as relative abundances (percentages) with the exception of George et al. (2021) [67], who reported daily intakes, and 3 studies which did not report any data on FAs except for effect sizes of relationships [36,38,74]. Following weight and height, head circumference was the third most consistently examined growth outcome across FAs, with 5 studies examining associations between linoleic acid (LA) and head circumference. This is likely because head circumference metrics are considered a proxy for brain growth, and certain FAs such as LA, docosahexaenoic acid (DHA), and arachidonic acid (ARA) are thought to contribute to neurodevelopment [98]. Body composition metrics such as fat mass, fat mass index, percent fat, and skinfolds were minimally examined within the fatty acid context, with only 2 studies (3 articles; [38,75,76]) examining these outcomes.

Most studies examining the relationship between relative abundance of individual FAs and infant growth reported no associations or demonstrated inconsistent results between studies. When significant associations were reported, these were only observed in one study and not repeated in other studies. Further, contradictory findings were observed for eicosapentaenoic acid (EPA) and weight between Miliku et al. (2019) at 3 mo of age [17] and Jacobsen et al. (2008) [74] at 6 mo of age.

Generally, any observed associations between SFAs and infant weight were positive, except for capric acid (10:0), which demonstrated an inverse association at 6 mo of age [74]. Further, capric acid, lauric acid (12:0) [74] and stearic acid (18:0) [77] were inversely associated with infant head circumference at 3 mo and 6 mo of age, but these associations have yet to be replicated independently in other studies. George et al. (2021) [67] and Jacobsen et al. (2008; unpublished data) [74] demonstrated contradicting associations between myristic acid (14:0) and infant head circumference in infants up to 9 mo of age.

Palmitoleic acid (16:1 n-7) was the only MUFA to demonstrate an inverse association with infant growth (length and weight), but only in one study at 3 mo of age [17]. All other observed associations between MUFAs and infant growth were positive for infants up to 6 mo of age as reported by George et al. (2021) [67].

Only 2 trans-FAs were investigated among the included studies. George et al. (2021) [85] observed positive associations between elaidic acid (ELA; 18:1t) concentrations and infant weight, length, BMI, and head circumference in infants up to 6 mo. However, these associations were not observed by Peng et al. (2021) [77] and Mychaleckyj et al. (2018) [99]. No studies observed associations between LA (18:2n-6t) and infant growth.

The omega-3 and omega-6 PUFAs were the most extensively explored categories of FAs (*n =* 14 studies), although only 5 studies examined total n-3 or total n-6 PUFAs as a composite group [40,68,78,79,100]. Among these studies, omega-6 PUFAs demonstrated inverse associations with infant weight at 1 y [80], and head circumference and percent fat at 4-8 wk [79] [37,79]. No directionally consistent associations were identified for composite groups of omega-3 PUFAs. Inverse associations were observed between omega-3 PUFAs and weight at 4-8 wk of age [43] and BMI z-scores [78] at 6 mo and one y, but a positive association was observed for fat percentage [40] and inconsistent directional associations were observed between Peng et al. (2021) [77] and Nuss et al. (2019) [40] for head circumference.

Docosahexanoic acid (DHA), LA, and EPA were examined by 11 studies [17,38,67,75,[78], [79], [80],[99], [100], [101]], 10 studies [17,38,67,74,[77], [78], [79],^80^,^82^,^83^] and 9 studies [17,38,67,75,[77], [78], [79], 83,101] respectively. Inverse associations were observed between DHA and infant weight at 1 and 3 mo [17,38], length at 3 mo and 1 y [17,75] and BMI Z-Score [78]. However, the association that De la Garza et al. (2019) [78] observed between DHA and infant BMI Z-Score at 1 y of age was from colostrum. Interestingly, given the established importance of DHA for brain development [102], no overall associations between DHA and head circumference were observed [15,100,101] across 3 studies of moderate quality examining this association using linear regression modeling. Generally, inverse associations were observed for DHA and infant weight [17,38] at 3 and 6 mo, length [17,75,76] at 3 mo and 1 y, and BMI z-scores [75,76,78]. Observations from the longitudinal INFAT study [75,76] demonstrated a positive association between DHA and BMI at the 1-y assessment point and an inverse association at the 2-y assessment point. The only other study that explored infant outcomes beyond 1 y of age was de la Garza Puentes et al. (2019) [78], who did not find any associations between DHA and BMI at 6 or 18 mo. Positive associations were observed between LA and many infant growth outcomes in infants up to 6 mo of age [67,78,82], whereas inverse associations were observed for Δ BMI (BMI gain), Δ weight (weight gain) over the first y of life [79] and percent fat mass at 6 mo of age [38]. Eicosatetraenoic acid demonstrated mixed associations with infant growth across 6 studies, where researchers observed positive associations at 6 mo [74] and negative associations at 3–4 mo of age [17,75] with infant weight and length.

Ding et al. (2021) [84] performed a principal component analysis of fatty acid composition and found that human milk FA patterns mainly composed of Long Chain-PUFAs, similar to those found in animal products (pork, beef, eggs, and fish) were associated with higher infant WAZ, LAZ and head circumference-for-age z-scores (HCAZ) in infants aged 30 to 50 d. Conversely, HM FA patterns more similar to those found in plants (e.g., rapeseed oil) were associated with lower infant HCAZ and LAZ scores.

Prentice et al. (2019) [53] examined the association between short-chain Fas (SCFAs) and infant growth. Using 1H-NMR spectra and GC-MS. They were able to detect butyrate, acetate, and formic acid, but not propionate in HM. They found inverse associations between butyrate and formate and infant BMI, as well as inverse associations between butyrate, formate, and acetate and skinfold thickness at 3 mo of age. Further research is warranted to replicate these novel exploratory results and investigate the potential role of SCFAs in HM.

#### Calculated daily intakes (CDI)

Four studies examined associations between CDI of HM fat and infant growth [[33], [45], [47], [50]]. Only Sims et al. (2020) [33] found any meaningful relationships, observing an inverse association between CDI of HM fat and infant WAZ in infants up to 9 mo of age. None of the other studies detected any significant associations between CDIs of HM fat and infant growth. George et al. (2021) [67] conducted an extensive analysis of the CDI of 46 FAs and infant growth in infants up to 6 mo of age. Mainly CDIs of pentadecanoic acid (C15:0), LA (C18:2) and α-Linolenic acid (C20:3), were positively related to infant growth outcomes whereas no relationships were detected between docosapentaenoic acid (C22:5) and EPA (C20:5) and infant head circumference.

#### Other HM Fat Components

Two studies examined associations between other HM fat components and infant growth outcomes. George et al. (2021) [85] examined the role of HM fat globules in infant growth and found positive associations between daily intakes of Ceramide d19: 1/22:0 and head circumference and phosphatidylinositol 38:5 and WLZ in infants up to 6 mo of age. Riederer et al. (2020) [41] examined the relationship between HM oxylipins in milk at 6 to 8 wk and infant growth at 14 to16 wk. They found a positive association between 11-Hydroxyeicosatetraenoic Acid and 13-HDHA (an autoxidation product of docosahexaenoic acid) together and fat mass index (adjusted) and an inverse association between 17-HDHA and FFM index. These were the only studies that reported these components, likely due to limited access to these technologies and the emerging nature of the assays used to measure them in HM [85].

## Discussion

### Key Findings

This systematic review of 57 studies identified consistent evidence that HM protein concentration is positively associated with infant length, whereas total and digestible carbohydrate concentrations tended to be positively associated with infant weight. There is limited evidence on the associations between individual amino acids and infant growth. Total fat concentrations had mixed associations with infant growth but generally demonstrated inverse associations with BMI, Δ BMI (BMI gain), and WAZ scores, and positive associations with weight and body fat metrics. However, many of the studies included were limited by suboptimal sampling strategies that do not account for HM fat variation throughout the day and between the fore and hind milk, which could have obscured important relationships. Finally, FAs demonstrated inconsistent associations with several infant growth metrics, although notably, this did not include any overall directional associations between DHA and head circumference.

Our finding of no consistent relationship between fat and infant growth outcomes could be related to a variety of factors. Fat content in HM is highly variable within a feed and across feeds during the day [15]. Most studies included in the current review used single milk sampling times, usually capturing early morning milk, and many did not account for transitions in fat content between the beginning and end of a feed. It is likely that the sampling protocols in these studies do not accurately reflect the true concentration of fat in HM, and results should be interpreted with caution. Additionally, it has been shown that both breastfed and formula feed infants have the ability to regulate their milk intake in response to its macronutrient content [13,103,104], which underscores the importance of collecting CDI to more accurately evaluate how fat and other macronutrient content in HM can impact infant growth.

DHA, ARA, and other long chain omega-3 FAs are common additives to formula, as they have long been thought to enhance infant brain development and growth [102]. Head circumference is a proxy measure for infant brain growth [105]. Interestingly, our findings did not demonstrate any directional associations between DHA or ARA and infant head circumference. This could be because these associations were analyzed using linear modeling, and the relationship between head circumference and DHA and ARA may not be linear [106]. Recent evidence indicates that the relationship between DHA and head circumference may follow a Z curve, requiring just the right amount of DHA to optimize head growth, with too little or too much potentially restricting head growth [106].

An interesting finding from this review was the potential link between fructose in HM and infant growth. While only one study examined this association, it was of high quality and did observe positive relationships between HM fructose levels and infant weight, WLZ, fat mass, and FFM [9]. This is an important area for further study, as previous interventional research has demonstrated that high-sugar maternal diets increase fructose levels in HM [10].

### Study design and inclusion criteria

Most of the studies (*n =* 38, 66%) in this review were longitudinal. However, 15 of these studies only sampled at 2 time points. There are increasing calls for researchers to assess infant anthropometrics longitudinally to better capture growth trajectories [107] as they demonstrate better predictive validity (compared to one-time measurements) for long term child health outcomes such as cognitive ability [107] and cardiovascular health [108]. Extending this approach to HM research and incorporating longitudinal measurements of both HM composition and infant anthropometrics would enable a deeper understanding of how HM influences growth trajectories and facilitate the identification of especially critical developmental periods during infancy.

In order to capture the full body of evidence on HM and infant growth, we did not limit our review to studies of exclusively breastfed infants, though we did capture this information in our quality assessment because exclusively breastfeeding dyads are the ideal study population for investigating the impact of HM composition on infant growth, particularly in the first 6 mo of life before the introduction of complementary foods. Relatively few studies (*n =* 26, 46%) were limited to exclusively breastfed infants, with the remainder involving “real world” populations that included breastfed infants supplemented with formula. Future research should address this limitation by focusing on exclusively breastfed infants or stratifying according to breastfeeding exclusivity so that associations in this sub-population can be clearly identified.

### HM sampling

Human milk composition is impacted by a multitude of factors, including lactation stage, infant gestational age, maternal health, parity, age, and diet [97], all of which are important to consider when developing milk sampling protocols. Fat is one of the most variable components in HM, with lower levels being observed in the morning and evening and higher levels observed during afternoon feeds [15]. In their work comparing 11 different sampling protocols, George et al. (2020) [15] determined that 6 prefeed and postfeed samples provided the most accurate estimate of lipid intake. Samples that were collected at the beginning of a feed first thing in the morning and prefeed samples from the most drained breast at any time through the day provided the greatest variation from true lipid volume intakes (±18 g/; ±300 kj. The majority of studies included in this review did not reflect this sampling strategy, and many relied on single time points to assess HM macronutrient concentrations. Collectively, the studies in this review highlight important considerations for planning or assessing HM fat research, as relationships between foremilk and/or hindmilk and infant development could be masked by sampling strategies that do not consider the change in milk fat content during a feed. Future research examining HM fat should consider sampling protocols that include expressing pre- and postfeed samples from each feed over a 24-h period [15]. Additionally, for all HM components, it is recommended that researchers employ measures to calculate daily intakes, such as weighing infants before and after each feed over a 24-h period to better reflect the amount of each component an infant consumes over the day rather than simply the concentration in one feed.

### Analytic Methods

Validated technologies and assays to assess HM composition are still emerging and have evolved considerably over the 35 y covered in this review. Accordingly, there was considerable heterogeneity in methods to assess HM components among the included studies. Many recent studies (since 2016), primarily conducted in high income settings, used HM analyzers to assess HM components, with the MIRIS system being the most common. HM analyzers are becoming more readily available for both clinical and research use and provide relatively consistent analytic strategies that can be compared across studies. However, these instruments have limitations, particularly for research purposes where sample volumes are often limited, and concerns have been raised regarding accuracy and precision in multicenter quality initiatives [95]. Established analytic methods such as the Kjeldahl method for HM proteins and Roese-Gottlieb or Folch for HM Fats are still the preferred methods for conducting HM research as they are the most accurate and reliable. However, the increasing accessibility and use of HM analyzers in research is evident from our findings. Their use in the literature and the results produced from these studies need to be considered with caution [95]. A recent study comparing 4 different macronutrient-based analytic methods for calculating calories in HM found considerable variation in caloric values 3.1 kcal/ounce (95% CI, 2.5, 3.7 kcal/ounce), a variation of 12–19% from the average of 19.4 ±1.4 kcal/ounce [94] between instruments. Analytic techniques that included digestible macronutrients (true protein, total fat, and lactose) compared to gross macronutrients (crude protein, total fat, and total carbohydrates) produced caloric values that were more conservative and likely more representative of bioavailability to the infant [94]. In the current study, there was considerable variation in the analytic strategies for protein and carbohydrate analysis. Seven of the 14 studies examining protein included crude protein in their analyses, while 5 studies that used the MIRIS did not indicate if they were analyzing crude or true protein values despite having the technology to report both. This lack of information is highly limiting for researchers to draw meaningful conclusions about HM macronutrient levels and infant growth [109].

An example of an area where future opportunities exist is around deeper exploration into SCFAs in HM, specifically those produced as postbiotics by microbiota, such as butyrate, formic acid, and acetate. With increasing emphasis placed on linking the microbiome in early life to infant health and growth [110], the origins and roles of SCFAs in HM are important to include in this body of evidence. Even so, the presence of these SCFAs in HM is poorly understood because assays quantifying these analytes are currently in the development phase and are inaccessible to many researchers [53]. However, preliminary evidence in this area, including one study examined in this review [53], indicates that SCFAs may have some relation to infant growth outcomes. Both the inclusion of HM SCFAs in human growth and development research and the improved assays to detect HM SCFAs are warranted because they can provide insight into the link between the human microbiome, HM composition, and infant growth.

### Anthropometrics

The review was complicated by the considerable variation in anthropometric outcome measurements that were reported across studies and timepoints. While most studies reported standardized infant growth measures (e.g., BMI, WAZ, LAZ), there were over 20 additional different anthropometric measures reported across these studies. This abundance of measurements made it challenging to consolidate the findings. Even among studies reporting standardized measures of infant growth, the standards varied (e.g., WHO and NCHS standards), which limits their comparability [111]. Among studies reporting body composition, extensive variation in measurement technologies persists, with some studies using X-ray (DEXA) technology and others using air-displacement technologies (PEAPOD). Overall, it was challenging to combine results across studies using a multitude of technologies and reporting standards. This highlights the issue of reliability and reproducibility in infant growth research, which has been a longstanding concern in the field [108].

### Systems analysis

Echoing the concerns expressed by Reyes et al. (2023) [112], there is a continued need to examine HM from a systems perspective. Many HM components are interrelated and should be considered in concert with each other. For example, post-biotic SCFAs present in milk may be metabolites from microbial species present in HM, or they may originate from the maternal gut microbiome [53]. Further, Wu et al. (2018) [97] posit that HM inflammatory factors may be related to fatty acid composition. However, these 2 components are often viewed separately. Additionally, viewing and studying the maternal-infant-milk triad as a system will help to develop a more fulsome understanding of HM composition and its association with infant growth [113]. For example, breastfeeding exclusivity and maternal factors such as diet and body composition can all impact milk production, which in turn can impact macronutrient intake by the infant [114]. This review highlights that many studies still do not account for breastfeeding exclusivity or other maternal factors that may impact secretory activation of several components in human milk. For example, among 18 studies examining HM FAs, all but one [84] assessed individual FAs one-by-one; the single study that applied a statistical strategy to capture the overall fatty acid “patterns” found associations providing unique information that could not be gleaned with traditional statistical approaches. Expanding this approach even further to examine HM composition across different ‘categories’ (e.g., different micronutrients, macronutrients, cells, microbes, bioactive proteins, etc.) and incorporating maternal and infant factors will help highlight the interplay between these components and provide enhanced knowledge about the interactions among various HM components and their collective impact on infant growth.

### Strengths and limitations

Strengths of our systematic review included use of a registered protocol and a comprehensive, peer-reviewed search strategy. Using SWiM [30] as a reporting guideline allowed us to present our findings using an accepted synthesis method. Across 3 reviews [5,112], we have comprehensively synthesized available evidence for HM composition and child anthropometrics in the first 2 ys of life. The main limitation of our review was our inability to overcome the wide variation in HM analysis techniques and infant anthropometrics among studies. Inconsistent growth standards and body composition technology, combined with multiple timepoint assessments, made assessing the primary outcome of infant growth challenging and prevented us from conducting a meta-analysis. As such, we summarized results in a heatmap format, which does not account for study size, strength of associations, or timepoint considerations. Individual studies included in this review also had limitations; only 11 of 57 (19%) studies achieved a high-quality score. Many studies did not fully describe the discrepancies between what macronutrient analyses measured and what was actually digestible to the infant (e.g., crude protein vs. true protein), which limited our ability to understand the role of these analytes on infant growth. Additionally, many studies did not adequately control for confounding (maternal BMI, birth anthropometrics, time postpartum, and HM exclusivity) and/or did not provide results for all examined outcomes. Finally, most studies measured macronutrient concentrations rather than CDI. As described above, infants may modify their HM intake based on macronutrient levels; therefore, assessing macronutrients in milk using concentrations from one feed is a substantial limitation and can lead to measurement bias.

## Conclusions

Macronutrients are likely the most extensively studied category of HM components, especially in relation to infant growth. The increased accessibility of HM analyzers has allowed researchers to use consistent analytic techniques that should increase comparability among studies, although these instruments have important limitations. Careful consideration is needed when developing milk sampling protocols, as the predominant technique of one sample per day is not sufficient for milk fat analysis.

We observed positive associations between HM carbohydrates and lactose with infant growth. Protein demonstrated a positive association with infant length but not weight; however, these results are reported using a mix of crude and true protein values and should be interpreted with caution. Finally, HM fat demonstrated mixed associations with infant growth, likely due to a large variation in sampling strategies and assessments of infant intake. Although many fatty acid concentrations were generally positively associated with head circumference, no studies found associations between DHA, SFAs, and n-6 PUFAs and head circumference.

Synthesis of the literature was limited by methodological issues with milk collection strategies and insufficient reporting of findings. Moving forward, researchers should consider using existing validated HM analytic techniques rather than HM analyzers to assess macronutrient content and develop sampling protocols that are reflective of the temporal variation in HM macronutrients, specifically fat content. Further, increased emphasis should be placed on investigating HM as a biological system that operates within the larger maternal-infant biological context rather than examining individual HM components in isolation.

## Acknowledgments

We thank Nicole Askin, MLIS (WRHA Virtual Library, University of Manitoba), for peer review of the MEDLINE search strategy. We also thank the International Milk Composition (IMiC) Consortium members for fruitful discussions that helped inform development of quality assessment and data extraction forms.

### Author contributions

MB, AID, SMR, and MBA designed the research. MB, AID, SMR, MBA, and NR oversaw the research. SMR, MB, JMM, DC, MG, RR, KKS, SM, PP, CM, and LL conducted the systematic review. MB, AID, SMR, LL, and MBA synthesized the data. MB and MBA wrote the paper and have primary responsibility for the final content. MB, AID, SMR, JMM, MG, DTG, FJ, PK, LHA, DH, KGE provided critical review and contribution to the manuscript. All authors have read and approved the final manuscript.

### Conflicts of interest

MB has contributed to online courses on breast milk and the infant microbiome produced by Microbiome Courses. SMR has contributed to online courses on breast milk and the infant microbiome produced by Microbiome Courses, serves as the scientific adviser for SimpliFed, and has served as a consultant for TraverseScience. She is a former employee of Prolacta Bioscience; her contribution to this review occurred prior to this employment. JMM has received support from the Bill & Melinda Gates Foundation and serves on the Council on Research for the American Academy of Nutrition and Dietetics. DC is supported by a Canadian Nurses Foundation Scholarship. DG is funded by an unrestricted research grant from Medela AG. She is also currently funded by Telethon Child Health Grants and the Australian National Health and Medical Research Council. LHA has research grants from the Bill & Melinda Gates Foundation. MBA is supported by a Canada Research Chair and is a CIFAR Fellow in the Humans and the Microbiome Program; she has consulted for DSM and is a scientific adviser to TinyHealth. MG, RR, KKS, SM, CM, FJ, PK, DH, LL, and KGE have no conflicts of interest.

### Funding

This review was undertaken as part of the International Milk Composition Consortium, funded by the Bill & Melinda Gates Foundation (INV-001734).

### Data availability

Data described in the manuscript, code book, and analytic code will be made available upon request pending application and approval by study authors.
