# Supplementary material for: Human Milk Macronutrients and Child Growth and Body Composition in the First Two Years: A Systematic Review
Source: Adv Nutr. 2023 Nov 18;15(1):100149. doi: 10.1016/j.advnut.2023.100149 (PMC10831902; doi:10.1016/j.advnut.2023.100149)
Supplement: Multimedia component 1 [file mmc1.pdf]

Table S1. Study quauality assessment criteria: human milk macronutrients and child growth.
Total score <7=Low, 7-13=Moderate, >13-17=High.

Item	Description	Points
**Human Milk Exposure Assessment**		<3=Low, 3-6=Moderate, >6-8=High
1	**Sampling strategy/ handling protocol**	
	**Lipids & Energy**	
1.1.0	Milk collection protocol	2 - Complete 24-hr collection OR combined aliquots of foremilk and hindmilk from one breast over 24 hours
		1 - Full breast expression standardized for time of day
		0 - Any other collection method
1.2.0	Stage of lactation	0.5 - Time postpartum standardized across participants
		0 - Time postpartum not standardized
1.3.0	Handling protocol	0.5 - Analyzed fresh or within 2 days of sampling
		0 - No precautions taken
	**Protein**	
1.1.1	Milk collection protocol	1 - Full breast expression or partial breast expression standardized across participants
		0 - Any other collection method
1.2.1	Stage of lactation	1 - Time postpartum standardized across participants
		0 - Time postpartum not standardized
1.3.1	Handling protocol	1 - Analyzed fresh or within 2 days of sampling
		0 - No precautions taken
	**Lactose**	
1.1.2	Milk collection protocol	1.5 - Full breast expression or partial breast expression standardized across participants
		0 - Any other collection method
1.2.2	Stage of lactation	1.5 - Time postpartum standardized across participants
		0 - Time postpartum not standardized
2	**Sample preparation**	
2.1	Reproducibility	1 - Reproducible using information provided in current paper OR direct reference
		0 - Not reproducible using information provided in current paper OR direct reference
3	**Analytical Method**	
3.1	Validation / quality control	2 - Method validated in human milk, including recovery data and inter-/intra-assay variation*** in current paper OR direct reference
		1 - Partial validation (e.g., precision or accuracy data reported, but not both in current paper OR direct reference)
		0.5 - Commercial assays or commonly used methods but no validation data specific to human milk in current paper OR direct reference
4	**Longitudinal sampling**	
4.1	Longitudinal sampling of human milk	1 - Milk samples collected longitudinally (e.g., 1, 3, and 6 mo)
		0 - Milk samples NOT collected longitudinally
5	**Human milk volume intake**	
5.1	Infant intake of human milk	1 - Volume of human milk infants consumed measured or estimated
		0 - Volume not considered
**Confounders Considered**		<3=Low, 3-4=Moderate, >4-5=High
6	**Infant diet**	
6.1	Breastfeeding exclusivity	2 - All infants exclusively breastfed at time of human milk collection
		up to 1.5 - If NOT all infants exclusively breastfed:
		0.5 - Reporting breastfeeding status
		0.5 - Adjusting for it in analyses
		0.5 - Reports number of proportion of human milk vs. other milks
		0 - Breastfeeding exclusivity of infants unclear
7	**Birth anthropometrics**	
7.1	Birth anthropometrics	1 - Any birth anthropometrics accounted for in study design or analyses
		0 - No birth anthropometrics accounted for
8	**Baseline characteristics**	
8.1	Maternal characteristics reported	0.5 - Reports maternal age parity, BMI, ethnicity, time postpartum, others relevant to study
		0 - Does not report maternal characteristics
8.2	Infant characteristics reported	0.5 - Reports infants age [or time postpartum] and sex
		0 - Does not report infant characteristics
8.2	Maternal characteristics accounted for	1 - Accounted for in analyses or study design
		0 - Not accounted for
8.3	Infant characteristics accounted for in analyses	1 - Accounted for in analyses or study design
		0 - Not accounted for
**Infant Anthropometric Outcomes**		<2=Low, 2-3=Moderate, >3-4=High
9	**Infant anthropometrics**	
9.1	Source of anthropometric measurements	1 - Trained staff
		0.5 - Collected from clinical records
		0 - Self-reported
9.2	Technical replicates performed?	1 - Yes
		0 - No
9.3	Longitudinal measurements	1 - Infant anthropometrics measured multiple times over time (e.g., 1, 3, and 6 mo)
		0 - Anthropometrics NOT measured longitudinally
9.4	Timing of measurements across infants	1 - All infants measured at same age (± 1 week)
		2 - All infants measured at similar age (± 1 month)
		0 - Timing of measurements not standardized across infants

***(e.g., data accuracy and precision)

Table S2. Study quality* assessment results: human milk macronutrients and child growth.

Study Details	Human Milk Exposure Assessment					Confounders Considered				Infant Anthropometric Outcomes				Summary & Total Scores**			
	1	2	3	4	5	6	7	8		9							
**Authors** (Alphabetical) **Country, Publication Year (Income Setting)**	**Sampling strategy/ handling protocol**	**Sample preparation**	**Analytical method**	**Longitudinal sampling**	**Human milk volume intake**	**Infant diet**	**Birth anthropometrics**	**Baseline characteristics reported**	**Baseline characteristics accounted for**	**Source of measurements**	**Techinical replicates**	**Longitudinal measurements**	**Standardization of timing of measurements**	**Human Milk Exposure Assessment**	**Confounders Considered**	**Infant Anthropometric Outcomes**	**TOTAL**
Abdelhamid et al. Egypt, 2020 (LMIC)	0.0	0.5	1.0	0.0	0.0	2.0	0.0	0.8	0.5	1.0	0.0	0.0	0.0	1.5	3.3	1.0	**5.8**
Aksit et al. Turkey, 2002 (LMIC)	1.3	1.0	0.8	0.0	1.0	2.0	1.0	1.0	0.5	1.0	0.0	0.0	1.0	4.0	4.5	2.0	**10.5**
Babiszewska Poland, 2020 (HIC)	0.0	1.0	0.0	0.0	0.0	2.0	1.0	0.8	0.0	1.0	0.0	0.0	0.0	2.0	3.1	1.5	**6.6**
Baldeón Ecuador, 2019 (LMIC)	3.0	1.0	2.0	1.0	0.0	2.0	1.0	0.8	0.0	1.0	0.0	1.0	1.0	7.0	3.8	3.0	**13.8**
Brown et al. Bangladesh, 1986 (LMIC)	2.3	1.0	2.0	1.0	1.0	1.3	0.5	0.9	0.5	1.0	0.5	1.0	1.0	7.3	3.1	3.5	**13.9**
Cissé et al. Senegal, 2002 (LMIC)	0.8	1.0	0.5	0.0	1.0	0.0	0.0	0.3	0.0	0.8	0.0	0.5	1.0	3.3	0.3	2.3	**5.8**
Cheema et al. Australia, 2021 (HIC)	2.0	1.0	2.0	0.0	1.0	2.0	1.0	1.0	1.0	1.0	0.0	0.0	1.0	6.0	5.0	2.0	**13.0**
deFluiter et al. Netherlands, 2021 (HIC)	1.0	0.5	2.0	1.0	0.0	1.0	1.0	1.0	1.0	1.0	1.0	1.0	1.0	4.5	4.0	4.0	**12.5**
De la Garza Puentes et al. Spain, 2019 (HIC)	2.5	1.0	2.0	1.0	0.0	1.0	0.5	1.0	1.0	1.0	0.0	1.0	1.0	6.5	3.5	3.0	**13.0**
De Luca et al. France, 2016 (HIC)	No quality assessment completed. Authors provided unpublished data.																**N/A**
Dewey et al. USA, 1993 (HIC)	2.5	1.0	2.0	0.5	1.0	2.0	0.5	0.9	0.5	0.8	0.5	1.0	1.0	7.0	3.9	3.3	**14.1**
Ding et al. China, 2021 (UMIC)	0.3	1.0	2.0	0.0	0.0	1.0	1.0	0.9	0.5	0.5	0.0	0.0	0.5	3.3	3.4	1.0	**7.6**
Dorea et al. Brazil, 1993 (LMIC)	2.0	1.0	2.0	1.0	0.0	0.0	0.5	0.6	0.5	1.0	0.0	1.0	1.0	6.0	1.6	3.0	**10.6**
Ellsworth et al. USA, 2020 (HIC)	1.3	0.5	1.5	0.0	0.0	0.5	0.5	0.9	0.8	0.6	0.0	1.0	0.8	3.3	2.6	2.4	**8.3**
Enstad et al. USA, 2019 (HIC)	0.5	1.0	0.8	1.0	0.0	1.0	1.0	0.8	1.0	1.0	0.0	1.0	0.8	3.3	3.8	2.8	**9.8**
Fields et al. USA, 2017 (HIC)	3.0	1.0	0.5	1.0	0.0	2.0	0.5	0.9	0.8	1.0	1.0	1.0	1.0	5.5	4.1	4.0	**13.6**
Fornes et al. Brazil, 1995 (LMIC)	1.0	1.0	2.0	1.0	0.5	2.0	1.0	1.0	1.0	1.0	0.0	1.0	1.0	5.5	5.0	3.0	**13.5**
George et al. Australia, 2021a (HIC)	1.5	1.0	2.0	1.0	1.0	1.0	1.0	1.0	0.0	0.5	0.0	0.0	1.0	6.5	3.0	1.5	**11.0**
George et al. Australia, 2021b (HIC)	1.5	1.0	1.0	1.0	1.0	1.0	1.0	1.0	0.0	1.0	0.0	1.0	1.0	5.5	3.0	3.0	**11.5**
Goran et al. USA, 2017 (HIC)	3.0	1.0	2.0	1.0	0.0	2.0	0.0	1.0	1.0	1.0	0.0	1.0	1.0	7.0	4.0	3.0	**14.0**
Gridneva et al. Australia, 2018 (HIC)	2.3	1.0	1.5	1.0	0.5	1.5	0.5	1.0	0.5	1.0	0.0	1.0	1.0	6.3	3.5	3.0	**12.8**
Gridneva et al. Australia, 2019 (HIC)	0.0	0.0	2.0	1.0	1.0	2.0	1.0	1.0	0.0	1.0	0.0	1.0	0.5	4.0	4.0	2.5	**10.5**
Gridneva et al. Australia, 2022 (HIC)	2.0	0.5	2.0	1.0	1.0	2.0	0.0	1.0	1.0	1.0	1.0	1.0	1.0	6.5	4.0	4.0	**14.5**
Isganaitis et al. USA, 2019 (HIC)	2.5	1.0	2.0	1.0	0.0	2.0	1.0	0.9	1.0	1.0	0.0	1.0	1.0	6.5	4.9	3.0	**14.4**
Jacobson et al. Canada, 2008 (HIC)	No quality assessment completed. Authors provided unpublished data.																**N/A**
Janas et al. USA, 1986 (HIC)	2.0	0.5	1.3	1.0	1.0	1.0	0.0	0.3	0.0	1.0	0.0	1.0	1.0	5.8	1.3	3.0	**10.0**
Kon et al. Russia, 2014 (LMIC)	0.3	0.5	0.3	1.0	1.0	0.5	0.0	0.0	0.0	1.0	0.0	1.0	1.0	3.0	0.5	3.0	**6.5**
Larnkjaer et al. Denmark, 2016 (HIC)	0.8	1.0	1.3	0.0	0.0	1.0	1.0	1.0	1.0	0.8	0.0	0.5	0.8	3.0	4.0	2.0	**9.0**
Larson-Meyer et al. USA, 2020 (HIC)	2.0	1.0	2.0	1.0	0.0	0.5	1.0	0.5	0.0	1.0	0.0	1.0	0.5	6.0	2.0	2.5	**10.5**
Larsson et al. Denmark, 2018 (HIC)	0.3	0.0	0.5	1.0	1.0	0.5	0.0	0.9	0.1	1.0	1.0	1.0	0.5	2.8	1.5	3.5	**7.8**
Makela et al. Finland, 2013 (HIC)	0.5	1.0	2.0	0.0	0.0	1.3	0.5	0.9	0.0	0.8	0.0	0.5	0.8	3.5	2.6	2.0	**8.1**
Martini et al. Indonesia, 2020 (LMIC)	0.5	1.0	1.0	1.0	0.0	2.0	0.5	1.0	0.0	1.0	0.5	1.0	0.8	3.5	3.5	3.3	**10.3**
Miliku et al. Canada, 2019 (HIC)	1.0	1.0	1.3	0.0	0.0	1.3	1.0	1.0	1.0	0.8	0.0	0.5	1.0	3.3	4.3	2.3	**9.8**
Miller et al. USA, 2017 (HIC)	0.5	0.5	1.0	0.0	1.0	1.0	0.5	0.9	1.0	1.0	0.0	0.0	0.0	3.0	3.4	1.0	**7.4**
Minato et al. Japan, 2019 (HIC)	1.0	1.0	0.5	1.0	0.0	0.8	1.0	0.4	0.5	0.5	0.0	1.0	1.0	3.5	2.6	2.5	**8.6**
Mitoulas et al. Australia, 2002 (HIC)	0.8	1.0	2.0	1.0	1.0	0.0	0.5	0.3	0.0	1.0	0.0	0.5	0.5	5.8	0.8	2.0	**8.5**

Table S2. Study quality* assessment results: human milk macronutrients and child growth.

Study Details	Human Milk Exposure Assessment					Confounders Considered				Infant Anthropometric Outcomes				Summary & Total Scores**			
Much et al. Germany, 2013 (HIC)	0.5	1.0	1.5	1.0	0.0	1.3	1.0	0.5	1.0	1.0	0.0	1.0	1.0	4.0	3.8	3.0	**10.8**
Meyer et al. Germany, 2019 (HIC)	0.5	1.0	1.0	1.0	0.0	0.5	0.0	1.0	1.0	1.0	0.0	1.0	0.5	3.5	2.0	2.5	**8.0**
Mychaleckyj et al. Bangladesh, 2020 (LMIC)	0.0	1.0	2.0	0.0	0.0	0.8	0.0	1.0	0.0	1.0	1.0	1.0	1.0	3.0	1.8	4.0	**8.8**
Nikniaz Jr. et al. Iran, 2009 (LMIC)	0.8	0.3	0.5	0.0	0.0	2.0	1.0	0.8	1.0	1.0	0.0	0.0	0.3	1.5	4.8	1.3	**7.5**
Nuss et al. USA, 2019 (HIC)	0.5	1.0	0.8	0.0	0.0	0.5	0.0	0.9	0.5	1.0	1.0	0.0	0.8	2.3	1.9	2.8	**6.9**
Palmer et al. Zambia, 2016 (LMIC)	0.5	1.0	2.0	1.0	0.0	0.3	0.0	0.5	0.5	1.0	0.0	1.0	0.3	4.5	1.3	2.3	**8.0**
Peng et al. China, 2021 (UMIC)	1.5	1.0	2.0	1.0	0.0	1.0	0.0	1.0	0.5	1.0	0.0	1.0	0.0	5.5	2.5	2.0	**10.0**
Prentice et al. United Kingdom, 2016 (HIC)	1.5	1.0	2.0	0.5	0.0	1.0	1.0	1.0	1.0	1.0	0.5	1.0	0.8	5.0	4.0	3.3	**12.3**
Prentice et al. United Kingdom, 2019 (HIC)	No quality assessment completed. Authors provided unpublished data.																**N/A**
Reiderer et al. Austria, 2020 (HIC)	0.5	1.0	0.5	0.0	0.0	1.5	1.0	1.0	1.0	1.0	0.0	0.0	0.5	2.0	4.5	1.5	**8.0**
Rudolph et al. USA, 2017 (HIC)	1.5	0.5	1.3	1.0	0.0	1.0	1.0	1.0	1.0	1.0	1.0	1.0	0.8	4.3	4.0	3.8	**12.0**
Saben et al. USA, 2021 (HIC)	1.5	0.0	0.5	1.0	1.0	1.5	1.0	1.0	1.0	1.0	1.0	1.0	0.5	4.0	4.5	3.5	**12.0**
Scholtens et al. Netherlands, 2009 (HIC)	1.5	0.3	0.9	1.0	0.5	1.3	1.0	1.0	1.0	1.0	1.0	1.0	0.6	4.1	4.3	3.6	**12.0**
Sims et al. USA, 2020 (HIC)	1.5	0.5	0.5	1.0	1.0	0.8	1.0	0.9	1.0	1.0	0.0	1.0	1.0	4.5	2.6	3.0	**10.1**
Tyson et al. USA, 1992 (HIC)	1.5	1.0	0.8	0.5	1.0	1.0	0.8	0.8	0.5	1.0	0.5	1.0	1.0	4.8	3.0	3.5	**11.3**
Ulloa et al. Argentina, 2020 (HIC)	0.0	0.0	0.5	0.0	0.0	1.0	0.0	1.0	1.0	1.0	0.0	1.0	0.0	0.5	3.0	2.0	**5.5**
Urteaga et al. Bolivia, 2018 (LMIC)	2.0	1.0	0.5	0.0	1.0	1.8	0.5	0.8	0.5	0.5	0.0	0.0	0.0	4.5	3.5	0.5	**8.5**
van Sadelhoff et al. Netherlands, 2018 (HIC)	1.0	1.0	1.5	1.0	0.0	0.0	0.5	0.5	0.5	0.5	0.5	1.0	1.0	4.5	1.5	3.0	**9.0**
van Sadelhoff et al. Germany, 2021 (HIC)	0.5	0.0	1.0	1.0	0.0	0.0	1.0	1.0	1.0	0.0	0.0	1.0	0.5	2.5	3.0	1.5	**7.0**
Xiang et al. China, 1999 (LMIC)	0.8	1.0	1.3	0.0	0.0	2.0	0.5	1.0	0.5	0.0	0.0	0.5	0.8	3.0	4.0	1.3	**8.3**
Xiang et al. Sweden, 2000 (HIC)	0.5	0.5	0.3	1.0	0.0	2.0	0.5	0.5	0.3	1.0	0.0	1.0	0.5	2.3	3.3	2.5	**8.0**
Zhang et al. China, 2021 (UMIC)	1.5	1.0	2.0	1.0	0.0	0.0	0.0	0.5	0.5	1.0	1.0	1.0	1.0	5.5	1.0	4.0	**10.5**

*See Table S1 for 9 categories of quality assessment criteria. **Red = low, Yellow = moderate, Green = high. See Methods for details.
World Bank Income Setting: LMIC, low middle income country; UMIC, upper middle income country; HIC, high income country.

Table S3. Characteristics and results of included studies reporting on human milk carbohydrates and infant anthropometrics - organized by component.

Authors, country, publication year (income setting)	Design and participants	Timing of milk sampling	Timing of infant anthropometrics	Estimated intake or HM concentration*	Anthropometric outcome measures and standards	Associations**	Major confounders considered
**Total carbohydrates**							
deFluiter et al.<br>Netherlands, 2021<br>(HIC)	Cohort<br><br>133	1 and 3 mo	1, 3, 6, 9, 12, 18 and 24 mo	1 month timepoint<br>Carbohydrate (g/100 ml): 8.7 [8.5-8.9]<br><br>3 month timepoint<br>Carbohydrate (g/100 ml): 8.7 [8.5-8.8]<br><br>MIRIS human milk analyser	Weight, length, head circumference, WFL, WFA, HFA (SDs), FMI, Body composition Abdominal fat mass	3 month timepoint<br>(No) Association between HM carbohydrates and infant weight, length, head circumference, WFL, WFA, HFA (SDs), FMI, Body composition, Abdominal fat mass<br><br>6 month timepoint<br>(No) Association between HM carbohydrates and infant weight, length, head circumference, WFL, WFA, HFA (SDs), FMI, Body composition, Abdominal fat mass	EBF during first 3 mo no other confounders reported
Ellsworth et al.<br>USA, 2020<br>(HIC)	Longitudinal<br>55 enrolled<br>32 analyzed	2 wks	2 wks and 2 mo	7.0 ± 0.3 g/dL in normal weight mothers<br>6.8 ± 0.5 g/dL in mothers with overweight or obesity<br><br>MIRIS human milk analyser	WLZ<br>BMIZ<br>WAZ<br>LAZ<br>HC<br>WHO reference standards	Difference from 2 wks to 2 mo<br>(No) Association between carbohydrates and WLZ change<br>(No) Association between carbohydrates and BMIZ change<br>(No) Association between carbohydrates and WAZ change<br>(No) Association between carbohydrates and LAZ change<br>(No) Association between carbohydrates and HC	Infant sex
Gridneva et al.<br>Australia, 2018<br>(HIC)<br><br>Gridneva et al.<br>Australia, 2019<br>(HIC)<br><br>Gridneva et al.<br>Australia, 2022<br>(HIC)	Longitudinal<br>22 enrolled<br>20 analyzed	2 and/or 5, 9, and 12 mo	2 and/or 5, 9, and 12 mo	2 mo timepoint<br>8.67 ± SD 0.92 g/dL<br><br>5 mo timepoint<br>8.07 ± 0.79 g/dL<br><br>9 mo timepoint<br>8.78 ± 1.11 g/dL<br><br>12 mo timepoint<br>8.84 ± 2.12 g/dL<br>(concentration in milk and calculated daily intake)<br>Enzymatic and UV spectrophotometry	Weight<br>BMI<br>Length<br>Body composition (including fat mass, fat mass index, fat-free mass, and fat-free mass index)<br><br>Infant abdominal adiposity (Subcutaneous-abdominal depth,Visceral depth, Visceral/subcutaneous-abdominal depths ratio, Preperitoneal fat area, Subcutaneous-abdominal depth, Subcutaneous-abdominal fat area, Preperitoneal/subcutaneous-abdominal fat areas ratio)	2 mo timepoint<br>(+) Association between carbohydrates and fat mass<br>(+) Association between carbohydrates and fat mass index<br>(+) Association between carbohydrates and fat mass %<br><br>5, 9, and 12 month timepoints<br>(-) Association between carbohydrates and fat mass<br>(-) Association between carbohydrates and fat mass index<br>(-) Association between carbohydrates and fat mass %<br><br>All timepoints up to 12 mo (linear mixed effects model accounting for month)(+) Association between carbohydrates and weight<br>(No) Association between carbohydrates and BMI<br>(+) Association between carbohydrates and length<br>(No) Association between carbohydrates and fat mass<br>(No) Association between carbohydrates and fat mass index<br>(+) Association between carbohydrates and fat-free mass (based on ultrasound 4-skinfolds, but not 2-skinfolds)<br>(+) Association between carbohydrates and fat-free mass index<br>(+) Association between total carbohydrate and subcutaneous abdominal fat area	Exclusive breastfeeding<br>Infant sex<br>Infant age
Minato et al.<br>Japan, 2019<br>(HIC)	Longitudinal<br>129 enrolled<br>88 analyzed at 1 month<br>56 analyzed at 3 mo	1 and 3 mo	1 and 3 mo	1 month timepoint<br>7.4 g/dL (IQR 7.0, 7.5 g/dL) for lower weight gain infants, 7.5 g/dL (IQR 7.2, 7.7 g/dL) for normal weight gain infants<br><br>3 mo timepoint<br>7.5 g/dL (IQR 7.5, 7.7 g/dL) for lower weight gain infants, 7.6 g/dL (IQR 7.2, 7.7 g/dL) for normal weight gain infants<br><br>MIRIS human milk analyzer	Weight (infants categorized by lower or normal weight gain)	1 month timepoint<br>(+) Association between carbohydrates and weight<br><br>3 mo timepoint<br>(No) Association between carbohydrates and weight	Exclusive breastfeeding
Ulloa et al.<br>Argentina, 2020<br>(HIC)	Cohort/Longitudinal<br>22 (n=13, EWG; n=9, AWG)	Protocol entry (4.34 [2.07 - 5.93] mo	Protocol entry and monthly thereafter until 1 year of age	No data reported<br><br>Difference between total solid content and protein, fats and ash content.	Weight, length, WFA, LFA, WFL (Z-Scores)	(No) Association between carbohydrates and excessive weight gain.	Exclusively breastfeeding
**Lactose**							
Abdelhamid et al.<br>Egypt, 2020 (LMIC)	Cross-sectional<br><br>100	6-14 mo of age	6-14 mo of age	Lactose (g/100 g)<br>1.06 ± 0.28<br><br>Lactose content measured calorimetrically<br><br>(concentration in milk)	Weight and length, BMI	(No) Association between HM lactose and length, weight or BMI	None
Cheema et al.<br>Australia, 2021<br>(HIC)	Longitudinal<br>67<br>(57 analysed)	2 mo	3 mo	Lactose concentration:<br>86.56 ± 7.91 (g/L)<br>Lactose CDI:<br>68.35 ± 16.63 (g/day)<br><br>Lactose colorimetric enzymatic assay<br><br>(daily intake and concentration)	Weight, length, BMI, head circumference, FFM, FFMI, FM, FMI, %FM, FM/FFM and z-scores<br><br>(WHO Standards)	(+) Association between lactose (CDI) and infant weight and length, adiposity, lean body mass (FFM and FFMI) and WAZ	Exclusive breastfeeding, Maternal age. ethnicity, parity, mode of delivery, height, weight, gestational age, sex, birth weight, birth length,
Goran et al.<br>USA, 2017<br>(HIC)	Longitudinal<br>37 enrolled<br>25 analyzed	1 and 6 mo	1 and 6 mo	1 month timepoint<br>7.8 ± 0.8 g/dL<br><br>6 mo timepoint<br>7.5 ± 0.7 g/dL<br><br>Liquid chromatography-mass spectrometry	Weight<br>Length<br>WLZ<br>Body composition (lean mass, fat mass, fat mass %)<br>WHO reference standards	6 mo timepoint (hierarchical regression model)<br>(No) Association between lactose and weight<br>(No) Association between lactose and length<br>(No) Association between lactose and WLZ<br>(No) Association between lactose and lean mass<br>(No) Association between lactose and fat mass<br>(No) Association between lactose and fat mass %	Infant sex<br>Infant weight<br>Maternal pre-pregnancy BMI

Table S3. Characteristics and results of included studies reporting on human milk carbohydrates and infant anthropometrics - organized by component.

Authors, country, publication year (income setting)	Design and participants	Timing of milk sampling	Timing of infant anthropometrics	Estimated intake or HM concentration*	Anthropometric outcome measures and standards	Associations**	Major confounders considered
Gridneva et al.<br>Australia, 2018<br>(HIC)<br><br>Gridneva et al.<br>Australia, 2019<br>(HIC)<br><br>Gridneva et al.<br>Australia, 2021<br>(HIC)	Longitudinal<br>22 enrolled<br>20 analyzed	2 and/or 5, 9, and 12 mo	2 and/or 5, 9, and 12 mo	2 mo timepoint<br>6.45 ± 0.41 g/dL<br><br>5 mo timepoint<br>6.43 ± 0.59 g/dL<br><br>9 mo timepoint<br>6.53 ± 0.53 g/dL<br><br>12 mo timepoint<br>6.69 ± 0.40 g/dL<br><br>Enzymatic and UV spectrophotometry	Weight<br>Length<br>BMI<br>Body composition (including fat mass, fat mass index, fat-free mass, and fat-free mass index)<br>Subcutaneous-abdominal depth,Visceral depth, Visceral/subcutaneous-abdominal depths ratio, Preperitoneal fat area, Subcutaneous-abdominal depth, Subcutaneous-abdominal fat area, Preperitoneal/subcutaneous-abdominal fat areas ratio	All timepoints up to 12 mo (linear mixed effects model accounting for month)(No) Association between lactose and weight<br>(No) Association between lactose and length<br>(No) Association between lactose and BMI<br>(No) Association between lactose and body composition<br>(+) Association between Lactose and subcutaneous abdominal fat area	Infant sex<br>Infant age
Larsson et al.<br>Denmark, 2018<br>(HIC)	Longitudinal<br>59 enrolled<br>30 analyzed	5 mo	5 mo	7.63 ± 0.22 g/dL in the high weight gain group,<br>7.58 ± 0.19 g/dL in the normal weight group<br><br>MIRIS human milk analyser	BMIZ<br>WAZ<br>LAZ<br>WHO reference standards	5 mo timepoint<br>(No) Association between lactose and BMIZ<br>(No) Association between lactose and WAZ<br>(No) Association between lactose and LAZ<br><br>Difference between birth and 5 mo<br>(No) Association between lactose and change in BMIZ<br>(No) Association between lactose and change in WAZ<br>(No) Association between lactose and change in LAZ	None reported<br><br>Exclusive breastfeeding
Martini et al.<br>Indonesia, 2020<br>(LMIC)	Longitudinal<br>40 enrolled<br>30 analyzed	1, 2, and 3 mo	1, 2, and 3 mo	1 month timepoint<br>3.77 ± 0.72 g/dL<br><br>2 mo timepoint<br>3.72 ± 0.79 g/dL<br><br>3 mo timepoint<br>4.17 ± 0.81 g/dL<br><br>MIRIS human milk analyser	Weight<br>Length<br>HC	1 month timepoint<br>(No) Association between lactose and weight<br>(No) Association between lactose and length<br>(No) Association between lactose and HC<br><br>2 mo timepoint<br>(No) Association between lactose and weight<br>(No) Association between lactose and length<br>(No) Association between lactose and HC<br><br>3 mo timepoint<br>(No) Association between lactose and weight<br>(No) Association between lactose and length<br>(No) Association between lactose and HC	None reported
Mitoulas et al.<br>Australia, 2002<br>(HIC)	Longitudinal<br>17	1, 2, 4, 6, 9, and 12 mo	6 mo	1-12 mo<br>6.14 (SE 0.06 g/dL)<br><br>(Estimated intake and concentration in HM)<br><br>Modified<br>method of Kuhn & Lowenstein	Weight	Difference between birth and 6 mo<br>(No) Association between lactose and weight gain	None reported
Prentice et al.<br>United Kingdom, 2016<br>(HIC)<br><br>Prentice et al.<br>United Kingdom, 2019<br>(HIC)	Longitudinal<br>619 (subset of cohort)	4-8 wks	3 and 12 mo	Median 8.6 g/dL (IQR 8.2-8.8 g/dL)<br><br><br>H-Nuclear magnetic resonance (NMR) spectra	Weight<br>BMI<br>Length<br>Body composition (skinfold thickness)	3 mo timepoint<br>(No) Association between lactose and weight<br>(No) Association between lactose and BMI<br>(No) Association between lactose and length<br>(No) Association between lactose and skinfold thickness<br><br>1 year timepoint<br>(No) Association between lactose and weight<br>(+) Association between lactose and BMI<br>(No) Association between lactose and length<br>(+) Association between lactose and skinfold thickness<br><br>Difference between 3 mo and 1 year<br>(+) Association between lactose and weight increase<br>(+) Association between lactose and BMI increase<br>(No) Association between lactose and length increase<br>(+) Association between lactose and skinfold thickness increase	Infant sex<br>Infant birthweight<br>Infant gestational age<br>Exclusive breastfeeding<br>Duration of sample storage
**Glucose**							
Cheema et al.<br>Australia, 2021<br>(HIC)	Longitudinal<br>67<br>(57 analysed)	2 mo	3 mo	Glucose concentration:<br>0.26 ± 0.09 (g/L)<br>Glucose CDI:<br>0.20 ± 0.09 (g/day)<br><br>Lactose colorimetric enzymatic assay<br><br>(daily intake and concentration)	Weight, length, BMI, head circumference, FFM, FFMI, FM, FMI, %FM, FM/FFM and z-scores<br><br>(WHO Standards)	(+) association between glucose (CDI) and infant head circumference<br>(no) association between glucose(CDI) and infant weight, length, BMI, FFM, FFMI, FM, FMI, %FM, FM/FFM	Exclusive breastfeeding, Maternal age. ethnicity, parity, mode of delivery, height, weight, gestational age, sex, birth weight, birth length
Fields et al.<br>USA, 2012<br>(HIC)	Longitudinal<br>37 enrolled<br>30 analyzed	1 month	1 and 6 mo	Mean 254 mg/dL (SD 90 mg/dL)<br><br>Glucose oxidase method	Weight<br>Length<br>Body composition (fat mass, fat-free mass, trunk fat mass, fat mass %)	(No) Association between glucose and weight<br>(No) Association between glucose and length<br>(No) Association between glucose and body composition	Infant sex<br>Infant age<br>Infant body composition at 1 month<br>Maternal pre-pregnancy BMI category

Table S3. Characteristics and results of included studies reporting on human milk carbohydrates and infant anthropometrics - organized by component.

Authors, country, publication year (income setting)	Design and participants	Timing of milk sampling	Timing of infant anthropometrics	Estimated intake or HM concentration*	Anthropometric outcome measures and standards	Associations**	Major confounders considered
Goran et al.<br>USA, 2017<br>(HIC)	Longitudinal<br>37 enrolled<br>25 analyzed	1 and 6 mo	1 and 6 mo	1 month timepoint<br>263.6 ± 87.5 μg/mL<br><br>6 mo timepoint<br>246.8 ± 76.8 μg/mL<br><br>Liquid chromatography-mass spectrometry	Weight<br>Length<br>WLZ<br>Body composition (lean mass, fat mass, fat mass %)<br>WHO reference standards	(No) Association between glucose and weight<br>(No) Association between glucose and length<br>(No) Association between glucose and WLZ<br>(No) Association between glucose and lean mass<br>(No) Association between glucose and fat mass<br>(No) Association between glucose and fat mass %	Infant sex<br>Infant weight<br>Maternal pre-pregnancy BMI
**Fructose**							
Goran et al.<br>USA, 2017<br>(HIC)	Longitudinal<br>37 enrolled<br>25 analyzed	1 and 6 mo	1 and 6 mo	1 month timepoint<br>7.2 ± 1.72 μg/mL<br><br>6 mo timepoint<br>6.3 ± 1.7 μg/mL<br><br>Liquid chromatography-mass spectrometry	Weight<br>Length<br>WLZ<br>Body composition (lean mass, fat mass, fat mass %)<br>WHO reference standards	(+) Association between fructose and weight<br>(No) Association between fructose and length<br>(+) Association between fructose and WLZ<br>(+) Association between fructose and lean mass<br>(+) Association between fructose and fat mass<br>(No) Association between fructose and fat mass %	Infant sex<br>Infant weight<br>Maternal pre-pregnancy BMI

*Values reported as mean ± SD or median (IQR). **"No (assumed) associations = unreported associations assumed to be no association.

Abbreviations: BF, breastfeeding; HIC, high income countries; mo, months; HM, human milk; LMIC, low and middle income countries; NCHS, National Center for Health Statistics; RCT, randomized controlled trial; SCM, subclinical mastitis; WHO, World Health Organization; wks, weeks

Anthropometrics: BMI, body mass indez; HAZ, height for age z-score; HC, head circumference; HCAZ, head circumference z-score; LAZ, length for age Z-score; LFA, length for age; WAZ, weight for age z-score; WFA, weight for age; WLZ, weight-for-length z-score

Table S4. Characteristics and results of included studies reporting on human milk proteins and amino acids and infant anthropometrics - organized by component.

Authors, country, publication year (income setting)	Design and participants	Timing of milk sampling	Timing of infant anthropometrics	Estimated intake or HM concentration*	Anthropometric outcome measures and standards	Associations**	Major confounders considered
**Protein**							
Abdelhamid et al. 2020, Egypt, (LMIC)	Cross-sectional<br><br>100	6-14 mo of age	6-14 mo of age	Protein (g/100 g)<br>1.06 (0.78 - 1.34)<br><br>Crude protein<br><br>Semi-micro Kjeldahl distillation method (concentration in milk)	Weight and length, BMI	(No) Association between HM protein and length, weight or BMI	None reported
deFluiter et al. 2021, Netherlands (HIC)	Cohort<br><br>133	1 and 3 mo	1, 3, 6, 9, 12, 18 and 24 mo	1 month timepoint<br>Crude protein (g/100 ml) 1.3 [1.1-1.5]<br>True protein (g/100 ml) 1.0 [0.9-1.2]<br>3 month timepoint<br>Crude protein (g/100 ml) 1.0 [0.9-1.2]<br>True protein (g/100 ml) 0.8 [0.8-0.9]<br><br>Crude protein, true protein<br><br>MIRIS human milk analyser (concentration)	Weight, length, head circumference, WFL, WFA, HFA (SDs), FMI, Body composition Abdominal fat mass	3 month timepoint<br>(No) Association between HM Protein and infant weight, length, head circumference, WFL, WFA, HFA (SDs), FMI, Body composition,<br>Abdominal fat mass<br><br>6 month timepoint<br>(-) Association between HM crude and true protein at 3 mo and visceral FM (cm) at 6 mo (beta 0.271 (0.539 to 0.004) and beta 0.335 (0.670 to 0.001), respectively, both p = 0.049)<br>(No) Association between HM Protein and infant anthropometrics	EBF during first 3 mo no other confounders reported
De Luca et al., 2016 France, (HIC)	Longitudinal<br>165 enrolled<br>100 analyzed	1 month	1 month	Mean 1.0 g/dL (95% CI 0.9-1.0 g/dL) in normal weight mothers, 1.0 g/dL (95% CI 0.9-1.0 g/dL)<br><br>Did not state whether crude or true protein was used in analysis<br><br>MIRIS human milk analyser	Weight<br>Length	1 month timepoint<br>(+) Association between protein and weight<br>(+) Association between protein and length<br><br>Difference from birth to 1 month<br>(No) Association between protein and weight gain<br>(+) Association between protein and length gain	Unadjusted estimates provided by authors<br><br>Exclusive breastfeeding
Dorea Brazil, 1993 (LMIC)	Longitudinal<br>8	Bi-weekly or monthly between birth and 6 mo	Bi-weekly or monthly between birth and 6 mo<br><br>Association of Official Agricultural Chemists (A. O.A.C.)	Data not presented	Weight<br>Height	Difference from birth to 6 mo<br>(+) Association between protein and weight gain<br>(No) Association between protein and length change	Zinc, total nitrogen, and fat in multiple regression
Ellsworth et al. USA, 2020 (HIC)	Longitudinal<br>55 enrolled<br>32 analyzed	2 wks	2 wks and 2 mo	Mean 1.2 g/dL (SD 0.2 g/dL) in normal weight mothers, 1.2 g/dL (SD 0.4 g/dL) in mothers with overweight or obesity<br><br>Did not state whether crude or true protein was used in analysis<br><br>MIRIS human milk analyser	WLZ<br>BMIZ<br>WAZ<br>LAZ<br>HC<br>WHO reference standards	Difference from 2 wks to 2 mo<br>(No) Association between protein and WLZ change<br>(No) Association between protein and BMIZ change<br>(No) Association between protein and WAZ change<br>(No) Association between protein and LAZ change<br>(No) Association between protein and HC change	Infant sex
Gridneva et al. Australia, 2018 (HIC)<br><br>Gridneva et al. Australia, 2019 (HIC)<br><br>Gridneva et al. Australia, 2021 (HIC)	Longitudinal<br>22 enrolled<br>20 analyzed	2 and/or 5, 9, and 12 mo	2 and/or 5, 9, and 12 mo	2 mo timepoint<br>Mean 1.10 g/dL (SD 0.14 g/dL)<br><br>5 mo timepoint<br>Mean 1.19 g/dL (SD 0.14 g/dL)<br><br>9 mo timepoint<br>Mean 0.97 g/dL (SD 0.11 g/dL)<br><br>12 mo timepoint<br>Mean 1.07 g/dL (SD 0.28 g/dL)<br><br>Total (crude) protein<br><br>Bradford Protein Assay adapted from Mitoulas et al.	Weight<br>Length<br>HC<br>Body composition (including fat mass, fat mass %, and fat mass index, fat-free mass, and fat-free mass index)<br><br>Infant abdominal adiposity (Subcutaneous-abdominal depth,Visceral depth, Visceral/subcutaneous-abdominal depths ratio, Preperitoneal fat area, Subcutaneous-abdominal depth, Subcutaneous-abdominal fat area, Preperitoneal/subcutaneous-abdominal fat areas ratio)	All timepoints up to 12 mo (linear mixed effects model accounting for month)<br>(No) Association between protein and weight<br>(No) Association between protein and length<br>(No) Association between protein and HC<br>(No) Association between protein and body composition<br>(+) Association between total protein and infant abdominal adiposity<br><br>(+) Association between total protein and visceral/subcutaneous-abdominal depths ratio<br><br>(No) Association between total protein and Preperitoneal fat area<br><br>(No) Association between total protein and Subcutaneous-abdominal fat area (cm2)	Exclusive breastfeeding<br>Infant sex<br>Infant age

Table S4. Characteristics and results of included studies reporting on human milk proteins and amino acids and infant anthropometrics - organized by component.

Authors, country, publication year (income setting)	Design and participants	Timing of milk sampling	Timing of infant anthropometrics	Estimated intake or HM concentration*	Anthropometric outcome measures and standards	Associations**	Major confounders considered
Kon et al.<br>Russia, 2014<br>(LMIC)	Longitudinal<br>103 enrolled<br>99 analyzed	1, 2, and 3 mo	1, 2, and 3 mo	1 month timepoint<br>Mean 1.77 g/dL (SEM 0.22 g/dL) for low weight gain group, 1.70 g/dL (SEM 0.10 g/dL) for normal weight gain group, 1.66 g/dL (SEM 0.16 g/dL) for high weight gain group<br><br>2 mo timepoint<br>Mean 1.68 g/dL (SEM 0.15 g/dL) for normal weight gain group, 1.77 g/dL (SEM 0.12 g/dL) for high weight gain group<br><br>6 mo timepoint<br>Mean 1.50 g/dL (SEM 0.09 g/dL) for low weight gain group, 1.32 g/dL (SEM 0.08 g/dL) for normal weight gain group, 1.50 g/dL (SEM 0.20 g/dL) for high weight gain group<br><br>Crude protein<br><br>Protein - Kjeldahl method	Weight gain (infants categorized by low, normal, and high weight gain)	1 month timepoint<br>(No) Association between protein and weight gain<br><br>2 mo timepoint<br>(No) Association between protein and weight gain<br><br>3 mo timepoint<br>(No) Association between protein and weight gain	None reported
Larsson et al.<br>Denmark, 2018<br>(HIC)	Longitudinal<br>59 enrolled<br>30 analyzed	5 mo	5 mo	Mean 0.73 g/dL (SD 0.11 g/dL) in the high weight gain group, 0.80 g/dL (SD 0.11 g/dL) in the normal weight group<br><br>Did not state whether crude or true protein was used in analysis<br><br>MIRIS human milk analyser	BMIZ<br>WAZ<br>LAZ<br>WHO reference standards	5 mo timepoint<br>(No) Association between protein and BMIZ<br>(No) Association between protein and WAZ<br>(No) Association between protein and LAZ<br><br>Difference between birth and 5 mo<br>(No) Association between protein and change in BMIZ<br>(No) Association between protein and change in WAZ<br>(No) Association between protein and change in LAZ	None reported<br><br>Exclusive breastfeeding
Martini et al.<br>Indonesia, 2020<br>(LMIC)	Longitudinal<br>40 enrolled<br>30 analyzed	1, 2, and 3 mo	1, 2, and 3 mo<br><br>(does not clarify if Crude or True Protein reported)<br><br>MIRIS human milk analyser	1 month timepoint<br>Mean 1.89 g/dL (SD 0.38 g/dL)<br><br>2 mo timepoint<br>Mean 1.83 g/dL (SD 0.43 g/dL)<br><br>3 mo timepoint<br>Mean 1.72 g/dL (SD 0.37 g/dL)<br><br>Did not state whether crude or true protein was used in analysis<br><br>MIRIS human milk analyser	Weight<br>Length<br>HC	1 month timepoint<br>(No) Association between protein and weight<br>(+) Association between protein and length<br>(+) Association between protein and HC<br><br>2 mo timepoint<br>(No) Association between protein and weight<br>(No) Association between protein and length<br>(No) Association between protein and HC<br><br>3 mo timepoint<br>(No) Association between protein and weight<br>(No) Association between protein and length<br>(No) Association between protein and HC	None reported
Minato et al.<br>Japan, 2019<br>(HIC)	Longitudinal<br>129 enrolled<br>88 analyzed at 1 month<br>56 analyzed at 3 mo	1 and 3 mo	1 and 3 mo	1 month timepoint<br>Median 1.5 g/dL (IQR 1.4, 1.8 g/dL) for lower weight gain infants, 1.4 g/dL (IQR 1.3, 1.6 g/dL) for normal weight gain infants<br><br>3 mo timepoint<br>Median 1.2 g/dL (IQR 1.0, 1.2 g/dL) for lower weight gain infants, 1.1 g/dL (IQR 1.0, 1.2 g/dL) for normal weight gain infants<br><br>Did not state whether crude or true protein was used in analysis<br><br>MIRIS human milk analyzer	Weight (infants categorized by lower or normal weight gain)	1 month timepoint<br>(-) Association between protein and weight<br><br>3 mo timepoint<br>(No) Association between protein and weight	Exclusive breastfeeding
Mitoulas et al.<br>Australia, 2002<br>(HIC)	Longitudinal<br>17	1, 2, 4, 6, 9, and 12 mo	6 mo	1-12 mo<br>Mean 0.916 g/dL (SE 0.019 g/dL)<br><br>Crude protein<br><br>Kjeldahl procedure	Weight	Difference between birth and 6 mo<br>(No) Association between protein and weight gain	None reported

Table S4. Characteristics and results of included studies reporting on human milk proteins and amino acids and infant anthropometrics - organized by component.

Authors, country, publication year (income setting)	Design and participants	Timing of milk sampling	Timing of infant anthropometrics	Estimated intake or HM concentration*	Anthropometric outcome measures and standards	Associations**	Major confounders considered
Prentice et al.<br>United Kingdom, 2016 (HIC)<br><br>Prentice et al.<br>United Kingdom, 2019 (HIC)	Longitudinal<br>619 (subset of cohort)	4-8 wks	3 and 12 mo	Median 1.2 g/dL (IQR 1.1-1.2 g/dL)<br><br>Crude protein<br><br>Dumas method	Weight<br>BMI<br>Length<br>Body composition (skinfold thickness)	3 mo timepoint<br>(No) Association between protein and weight<br>(No) Association between protein and BMI<br>(No) Association between protein and length<br>(No) Association between protein and skinfold thickness<br><br>1 year timepoint<br>(No) Association between protein and weight<br>(No) Association between protein and BMI<br>(No) Association between protein and length<br>(No) Association between protein and skinfold thickness<br><br>Difference between 3 mo and 1 year<br>(No) Association between protein and weight change<br>(No) Association between protein and BMI change<br>(No) Association between protein and length change<br>(No) Association between protein and skinfold thickness change	Infant sex<br>Infant birthweight<br>Infant gestational age<br>Exclusive breastfeeding<br>Duration of sample storage
Ulloa et al. 2020, Argentina, (HIC)	Longitudinal<br>22 (n=13, EWG; n=9, AWG)	Protocol entry (4.34 [2.07 - 5.93] mo	Protocol entry and monthly thereafter until 1 year of age	Protein, (concentrations)<br><br>Total (crude) protein<br><br>Semi-automated protein analyser	Weight, length, WFA, LFA, WFL (Z-Scores)	(No) Association between protein and excessive weight gain	Exclusively breastfeeding
Zhang et al. 2021, China (UMIC)	Cohort<br><br>105	8–14 days, 1 month, and 6 mo	8–14 days, 1 month, and 6 mo	Total protein (mg/100mL)<br>1-5 days<br>1662.2 (447.7)<br>8-14 days<br>1545.2 (416.7)<br>1 month<br>1368.3 (406.3)<br>6 mo<br>993.9 (335.0)<br>(concentration)<br><br>Total (crude) protein<br><br>Bradford method	Length, weight, Z-scores LFA, WFA, WFL<br><br>(WHO)	1 month<br>(No) Association between total protein and infant anthropometry<br>6 mo<br>(No) Association between total protein and infant anthropometry	Postpartum time (wks), maternal age, education, household income, pre-gestational BMI, mode of delivery, parity, and gender of infant
**Essential amino acids**							
**Histidine**							
Baldeón<br>Ecuador, 2019 (LMIC)	Longitudinal<br>65 enrolled<br>61 analyzed at 1 week<br>47 analyzed at 2 wks<br>38 analyzed at 2 mo<br>37 analyzed at 4 mo	1 week, 2 wks, 2 mo and 4 mo	1 week, 2 wks, 2 mo and 4 mo	Data not presented<br><br>(concentrations in milk)<br><br>Automatic amino acid analyzer (model L-8900; Hitachi, Tokyo, Japan) for cation-exchange chromatography separation	Weight (children categorized by weight gain tertiles)<br>HC (children categorized by HC gain tertiles)	Difference between 1 week and 4 mo<br>(No) Association between histidine and weight gain<br>(No) Association between histidine and HC gain	Exclusive breastfeeding only<br>Infant sex
Reiderer et al. 2020 Austria (HIC)	Cohort<br><br>54 (47 analysed)	6-8 wks	14-16 wks	Amino Acid; median (IQR), µmol/l<br><br>26.39 (12.76)	Length. weight, body composition (FM, FFM), FMI< FFMI, BMI<br><br>(WHO)	No Associations reported for HM Amino Acids and infant anthropometry	BMI/Age ZScore, gestational weight gain, infant feeding mode (Fully BF, not fully BF)
Saben et al. 2021, USA (HIC)	Longitudinal (2 studies)<br>194 (normal weight, n= 68; OW, n=51; OB, n= 75)	0.5 mo<br>2 mo<br>6 mo	0.5 mo<br>2 mo<br>6 mo	0.5 mo (nmol/ml)<br>NW: 32.2 ± 1.7<br>OB: 22.1 ± 1.9<br>2 mo (nmol/ml)<br>NW: 33.5 ± 1.7<br>OB: 22.7 ± 1.6<br>6 mo (nmol/ml)<br>NW:28.9 ± 1.4<br>OB: 17.7 ± 1.7	Weight, length, Weight for GA, WAZ, WLZ, FMI, FFMI (WHO)	(No) Association between infant free amino acid intake and WAZ<br>(+) Association between [His] and WLZ<br>(+) Association between [His] and FMI.<br>(+) Association between [His] and FFMI.	Infant sex, body composition, birth weight
vanSadelhoff 2021, Germany (HIC)	Cohort<br><br>741 (441 analysed)	6 wks and 6 mo	6 wks and 6 mo	Free AAs (µmol/L):<br><br>6 wks:<br>28.0 (9.1)<br>6 mo :<br>29.7 (13.9)	Weight, length, weight gain, length gain. (Not WHO)	(No) Association between Histidine and infant growth<br>(-) Association between all (total) FAAs (free AAs) and infant weight gain at 6 wks	
**Isoleucine**							

Table S4. Characteristics and results of included studies reporting on human milk proteins and amino acids and infant anthropometrics - organized by component.

Authors, country, publication year (income setting)	Design and participants	Timing of milk sampling	Timing of infant anthropometrics	Estimated intake or HM concentration*	Anthropometric outcome measures and standards	Associations**	Major confounders considered
Baldeón Ecuador, 2019 (LMIC)	Longitudinal<br>65 enrolled<br>61 analyzed at 1 week<br>47 analyzed at 2 wks<br>38 analyzed at 2 mo<br>37 analyzed at 4 mo	1 week, 2 wks, 2 mo and 4 mo	1 week, 2 wks, 2 mo and 4 mo	Data not presented (concentrations in milk)<br><br>Automatic amino acid analyzer (model L-8900; Hitachi, Tokyo, Japan) for cation-exchange chromatography separation	Weight (children categorized by weight gain tertiles)<br>HC (children categorized by HC gain tertiles)	Difference between 1 week and 4 mo<br>(No) Association between isoleucine and weight gain<br>(No) Association between isoleucine and HC gain	Exclusive breastfeeding only<br>Infant sex
Reiderer et al. 2020 Austria (HIC)	Cohort<br><br>54 (47 analysed)	6-8 wks	14-16 wks	Amino Acid; median (IQR), μmol/l<br><br>6.04 (5.62)	Length. weight, body composition (FM, FFM), FMI< FFMI, BMI<br><br>(WHO)	No Associations reported for HM Amino Acids and infant anthropometry	BMI/Age ZScore, gestational weight gain, infant feeding mode (Fully BF, not fully BF)
Saben et al. 2021, USA (HIC)	Longitudinal (2 studies)<br>194 (normal weight, n= 68; OW, n=51; OB, n= 75)	0.5 mo<br>2 mo<br>6 mo	0.5 mo<br>2 mo<br>6 mo	0.5 mo (nmol/ml)<br>NW: 10.3 ± 1.0<br>OB: 16.7 ± 1.2<br>2 mo (nmol/ml)<br>NW: 10.4 ± 0.6<br>OB: 16.3 ± 1.0<br>6 mo (nmol/ml)<br>NW:10.3 ± 0.5<br>OB: 14.6 ± 1.0	Weight, length, Weight for GA, WAZ, WLZ, FMI, FFMI (WHO)	(No) Association between isoleucine intake or isoleucine concentrations and infant outcomes	Infant sex, body composition, birth weight
vanSadelhoff 2021, Germany (HIC)	Cohort<br><br>741 (441 analysed)	6 wks and 6 mo	6 wks and 6 mo	Free AAs (μmol/L):<br><br>6 wks:<br>15.9 (9.8)<br>6 mo :<br>12.7 (5.9)	Weight, length, weight gain, length gain. (Not WHO)	(No) Association between isoleucine intake or isoleucine concentrations and infant outcomes<br><br>(-) Association between all (total) FAAs (free AAs) and infant weight gain at 6 wks	
**Leucine**							
Baldeón Ecuador, 2019 (LMIC)	Longitudinal<br>65 enrolled<br>61 analyzed at 1 week<br>47 analyzed at 2 wks<br>38 analyzed at 2 mo<br>37 analyzed at 4 mo	1 week, 2 wks, 2 mo and 4 mo	1 week, 2 wks, 2 mo and 4 mo	Data not presented (concentrations in milk)<br><br>Automatic amino acid analyzer (model L-8900; Hitachi, Tokyo, Japan) for cation-exchange chromatography separation	Weight (children categorized by weight gain tertiles)<br>HC (children categorized by HC gain tertiles)	Difference between 1 week and 4 mo<br>(No) Association between leucine and weight gain<br>(No) Association between leucine and HC gain	Infant sex<br>Exclusive breastfeeding only
Reiderer et al. 2020 Austria (HIC)	Cohort<br><br>54 (47 analysed)	6-8 wks	14-16 wks	Amino Acid; median (IQR), μmol/l<br><br>40.42 (20.25)	Length. weight, body composition (FM, FFM), FMI< FFMI, BMI<br><br>(WHO)	No Associations reported for HM Amino Acids and infant anthropometry	BMI/Age ZScore, gestational weight gain, infant feeding mode (Fully BF, not fully BF)
Saben et al. 2021, USA (HIC)	Longitudinal (2 studies)<br>194 (normal weight, n= 68; OW, n=51; OB, n= 75)	0.5 mo<br>2 mo<br>6 mo	0.5 mo<br>2 mo<br>6 mo	0.5 mo (nmol/ml)<br>NW: 25.5 ± 2.6<br>OB: 36.7 ± 2.7<br>2 mo (nmol/ml)<br>NW: 27.5 ± 1.2<br>OB: 37.0 ± 2.3<br>6 mo (nmol/ml)<br>NW: 29.2 ± 1.1<br>OB: 33.5 ± 1.9	Weight, length, Weight for GA, WAZ, WLZ, FMI, FFMI (WHO)	(No) Association between leucine intake or leucine concentrations and infant outcomes	Infant sex, body composition, birth weight
vanSadelhoff 2021, Germany (HIC)	Cohort<br><br>741 (441 analysed)	6 wks and 6 mo	6 wks and 6 mo	Free AAs (μmol/L):<br><br>6 wks:<br>32.8 (15.7)<br>6 mo :<br>34.9 (11.4)	Weight, length, weight gain, length gain. (Not WHO)	(No) Association between leucine intake or leucine concentrations and infant outcomes<br><br>(-) Association between all (total) FAAs (free AAs) and infant weight gain at 6 wks	
**Lysine**							
Baldeón Ecuador, 2019 (LMIC)	Longitudinal<br>65 enrolled<br>61 analyzed at 1 week<br>47 analyzed at 2 wks<br>38 analyzed at 2 mo<br>37 analyzed at 4 mo	1 week, 2 wks, 2 mo and 4 mo	1 week, 2 wks, 2 mo and 4 mo	Data not presented (concentrations in milk)<br><br>Automatic amino acid analyzer (model L-8900; Hitachi, Tokyo, Japan) for cation-exchange chromatography separation	Weight (children categorized by weight gain tertiles)<br>HC (children categorized by HC gain tertiles)	Difference between 1 week and 4 mo<br>(No) Association between lysine and weight gain<br>(No) Association between lysine and HC gain	Infant sex<br>Exclusive breastfeeding only

Table S4. Characteristics and results of included studies reporting on human milk proteins and amino acids and infant anthropometrics - organized by component.

Authors, country, publication year (income setting)	Design and participants	Timing of milk sampling	Timing of infant anthropometrics	Estimated intake or HM concentration*	Anthropometric outcome measures and standards	Associations**	Major confounders considered
Isganaitis et al.<br>USA, 2019<br>(HIC)	Longitudinal<br>37 enrolled<br>31 at 1 month<br>26 at 6 mo	1 and 6 mo	1 and 6 mo	Data not presented	Body composition (including fat mass %, fat accrual between 1-6 mo)<br>Weight	1 month timepoint<br>(-) Association between lysine and weight<br><br>6 mo timepoint<br>(-) Association between lysine and fat mass %<br><br>Difference between 1-6 mo<br>(-) Association between lysine and fat accrual	Infant sex<br>Infant gestational age<br>Infant birthweight<br>Maternal parity
Reiderer et al. 2020<br>Austria (HIC)	Cohort<br><br>54 (47 analysed)	6-8 wks	14-16 wks	Amino Acid; median (IQR), μmol/l<br><br>10.52 (5.03)	Length. weight, body composition (FM, FFM), FMI< FFMI, BMI<br><br>(WHO)	No Associations reported for HM Amino Acids and infant anthropometry	BMI/Age ZScore, gestational weight gain, infant feeding mode (Fully BF, not fully BF)
Saben et al. 2021, USA (HIC)	Longitudinal (2 studies)<br>194 (normal weight, n= 68; OW, n=51; OB, n= 75)	0.5 mo<br>2 mo<br>6 mo	0.5 mo<br>2 mo<br>6 mo	0.5 mo (nmol/ml)<br>NW: 33.8 ± 3.2<br>OB: 44.1 ± 4.3<br>2 mo (nmol/ml)<br>NW: 21.6 ± 2.2<br>OB: 24.9 ± 2.7<br>6 mo (nmol/ml)<br>NW:19.8 ± 1.9<br>OB: 21.0 ± 2.2	Weight, length, Weight for GA, WAZ WLZ, FMI, FFMI (WHO)	(No) Association between lysine intake or lysine concentrations and infant outcomes	Infant sex, body composition, birth weight
vanSadelhoff 2021, Germany (HIC)	Cohort<br><br>741 (441 analysed)	6 wks and 6 mo	6 wks and 6 mo	Free AAs (μmol/L):<br><br>6 wks:<br>32.3 (20.4)<br>6 mo :<br>30.2 (15.0)	Weight, length, weight gain, length gain. (Not WHO)	(No) Association between lysine intake or lysine concentrations and infant outcomes<br><br>(-) Association between all (total) FAAs (free AAs) and infant weight gain at 6 wks	
**Methionine**							
Baldeón<br>Ecuador, 2019<br>(LMIC)	Longitudinal<br>65 enrolled<br>61 analyzed at 1 week<br>47 analyzed at 2 wks<br>38 analyzed at 2 mo<br>37 analyzed at 4 mo	1 week, 2 wks, 2 mo and 4 mo	1 week, 2 wks, 2 mo and 4 mo	Data not presented (concentrations in milk)<br><br>Automatic amino acid analyzer (model L-8900; Hitachi, Tokyo, Japan) for cation-exchange chromatography separation	Weight (children categorized by weight gain tertiles)<br>HC (children categorized by HC gain tertiles)	Difference between 1 week and 4 mo<br>(No) Association between methionine and weight gain<br>(No) Association between methionine and HC gain	Infant sex<br>Exclusive breastfeeding only
Isganaitis et al.<br>USA, 2019<br>(HIC)	Longitudinal<br>37 enrolled<br>31 at 1 month<br>26 at 6 mo	1 and 6 mo	1 and 6 mo	Data not presented	Body composition (including fat mass %, fat accrual between 1-6 mo)<br>Weight	1 month timepoint<br>(-) Association between methionine and weight	Infant sex<br>Infant gestational age<br>Infant birthweight<br>Maternal parity
Reiderer et al. 2020<br>Austria (HIC)	Cohort<br><br>54 (47 analysed)	6-8 wks	14-16 wks	Amino Acid; median (IQR), μmol/l<br><br>4.43 (2.44)	Length. weight, body composition (FM, FFM), FMI< FFMI, BMI<br><br>(WHO)	No Associations reported for HM Amino Acids and infant anthropometry	BMI/Age ZScore, gestational weight gain, infant feeding mode (Fully BF, not fully BF)
vanSadelhoff 2021, Germany (HIC)	Cohort<br><br>741 (441 analysed)	6 wks and 6 mo	6 wks and 6 mo	Free AAs (μmol/L):<br><br>6 wks:<br>29.5 (22.7)<br>6 mo :<br>34.6 (22.7)	Weight, length, weight gain, length gain. (Not WHO)	(No) Associations reported for methionine and infant anthropometry<br>(-) Association between all (total) FAAs (free AAs) and infant weight gain at 6 wks	
**Phenylalanine**							
Baldeón<br>Ecuador, 2019<br>(LMIC)	Longitudinal<br>65 enrolled<br>61 analyzed at 1 week<br>47 analyzed at 2 wks<br>38 analyzed at 2 mo<br>37 analyzed at 4 mo	1 week, 2 wks, 2 mo and 4 mo	1 week, 2 wks, 2 mo and 4 mo	Data not presented (concentrations in milk)<br><br>Automatic amino acid analyzer (model L-8900; Hitachi, Tokyo, Japan) for cation-exchange chromatography separation	Weight (children categorized by weight gain tertiles)<br>HC (children categorized by HC gain tertiles)	Difference between 1 week and 4 mo<br>(No) Association between phenylalanine and weight gain<br>(No) Association between phenylalanine and HC gain	Infant sex<br>Exclusive breastfeeding only
Reiderer et al. 2020<br>Austria (HIC)	Cohort<br><br>54 (47 analysed)	6-8 wks	14-16 wks	Amino Acid; median (IQR), μmol/l<br><br>12.10 (5.30)	Length. weight, body composition (FM, FFM), FMI< FFMI, BMI<br><br>(WHO)	No Associations reported for HM Amino Acids and infant anthropometry	BMI/Age ZScore, gestational weight gain, infant feeding mode (Fully BF, not fully BF)

Table S4. Characteristics and results of included studies reporting on human milk proteins and amino acids and infant anthropometrics - organized by component.

Authors, country, publication year (income setting)	Design and participants	Timing of milk sampling	Timing of infant anthropometrics	Estimated intake or HM concentration*	Anthropometric outcome measures and standards	Associations**	Major confounders considered
Saben et al. 2021, USA (HIC)	Longitudinal (2 studies)<br>194 (normal weight, n= 68; OW, n=51; OB, n= 75)	0.5 mo<br>2 mo<br>6 mo	0.5 mo<br>2 mo<br>6 mo	0.5 mo (nmol/ml)<br>NW: 10.1 ± 0.6<br>OB: 13.2 ± 0.8<br>2 mo (nmol/ml)<br>NW: 11.9 ± 0.7<br>OB: 14.3 ± 0.8<br>6 mo (nmol/ml)<br>NW: 12.3 ± 0.7<br>OB: 16.2 ± 1.1	Weight, length, Weight for GA, WAZ WLZ, FMI, FFMI (WHO)	(No) Association between phenylalanine intake or phenylalanine concentrations and infant outcomes	Infant sex, body composition, birth weight
vanSadelhoff 2021, Germany (HIC)	Cohort<br><br>741 (441 analysed)	6 wks and 6 mo	6 wks and 6 mo	Free AAs (µmol/L):<br><br>6 wks:<br>21.3 (10.1)<br>6 mo :<br>16.5 (5.6)	Weight, length, weight gain, length gain. (Not WHO)	(No) Association between phenylalanine intake or phenylalanine concentrations and infant outcomes<br><br>(-) Association between all (total) FAAs (free AAs) and infant weight gain at 6 wks	
**Threonine**							
Baldeón<br>Ecuador, 2019<br>(LMIC)	Longitudinal<br>65 enrolled<br>61 analyzed at 1 week<br>47 analyzed at 2 wks<br>38 analyzed at 2 mo<br>37 analyzed at 4 mo	1 week, 2 wks, 2 mo and 4 mo	1 week, 2 wks, 2 mo and 4 mo	Data not presented<br>(concentrations in milk)<br><br>Automatic amino acid analyzer (model L-8900; Hitachi, Tokyo, Japan) for cation-exchange chromatography separation	Weight (children categorized by weight gain tertiles)<br>HC (children categorized by HC gain tertiles)	Difference between 1 week and 4 mo<br>(No) Association between threonine and weight gain<br>(No) Association between threonine and HC gain	Infant sex<br>Exclusive breastfeeding only
Isganaitis et al.<br>USA, 2019<br>(HIC)	Longitudinal<br>37 enrolled<br>31 at 1 month<br>26 at 6 mo	1 and 6 mo	1 and 6 mo	Data not presented	Body composition (including fat mass %, fat accrual between 1-6 mo)<br>Weight	1 month timepoint<br>(+) Association between threonine and fat mass %	Infant sex<br>Infant gestational age<br>Infant birthweight<br>Maternal parity
Reiderer et al. 2020 Austria (HIC)	Cohort<br><br>54 (47 analysed)	6-8 wks	14-16 wks	Amino Acid; median (IQR), µmol/l<br><br>90.18 (36.89)	Length. weight, body composition (FM, FFM), FMI< FFMI, BMI<br><br>(WHO)	No Associations reported for HM Amino Acids and infant anthropometry	BMI/Age ZScore, gestational weight gain, infant feeding mode (Fully BF, not fully BF)
vanSadelhoff 2021, Germany (HIC)	Cohort<br><br>741 (441 analysed)	6 wks and 6 mo	6 wks and 6 mo	Free AAs (µmol/L):<br><br>6 wks:<br>70.8 (30.6)<br>6 mo :<br>97.1 (37.2)	Weight, length, weight gain, length gain. (Not WHO)	(-) Association between Threonine and infant weight gain at 6 wks<br><br>(No) Association between Threonine intake or Threonine concentrations and other infant outcomes<br><br>(-) Association between all (total) FAAs (free AAs) and infant weight gain at 6 wks	
**Valine**							
Baldeón<br>Ecuador, 2019<br>(LMIC)	Longitudinal<br>65 enrolled<br>61 analyzed at 1 week<br>47 analyzed at 2 wks<br>38 analyzed at 2 mo<br>37 analyzed at 4 mo	1 week, 2 wks, 2 mo and 4 mo	1 week, 2 wks, 2 mo and 4 mo	Data not presented	Weight (children categorized by weight gain tertiles)<br>HC (children categorized by HC gain tertiles)	Difference between 1 week and 4 mo<br>(No) Association between valine and weight gain<br>(No) Association between valine and HC gain	Infant sex<br>Exclusive breastfeeding only
Reiderer et al. 2020 Austria (HIC)	Cohort<br><br>54 (47 analysed)	6-8 wks	14-16 wks	Amino Acid; median (IQR), µmol/l<br><br>57.48 (19.58)	Length. weight, body composition (FM, FFM), FMI< FFMI, BMI<br><br>(WHO)	No Associations reported for HM Amino Acids and infant anthropometry	BMI/Age ZScore, gestational weight gain, infant feeding mode (Fully BF, not fully BF)
Saben et al. 2021, USA (HIC)	Longitudinal (2 studies)<br>194 (normal weight, n= 68; OW, n=51; OB, n= 75)	0.5 mo<br>2 mo<br>6 mo	0.5 mo<br>2 mo<br>6 mo	0.5 mo (nmol/ml)<br>NW: 40.7 ± 2.2<br>OB: 48.5 ± 2.6<br>2 mo (nmol/ml)<br>NW: 47.7 ± 2.2<br>OB: 49.6 ± 1.9<br>6 mo (nmol/ml)<br>NW:42.8 ± 2.0<br>OB: 44.9 ± 2.5	Weight, length, Weight for GA, WAZ, WLZ, FMI, FFMI (WHO)	(No) Association between valine intake or valine concentrations and infant outcomes	Infant sex, body composition, birth weight
vanSadelhoff 2021, Germany (HIC)	Cohort<br><br>741 (441 analysed)	6 wks and 6 mo	6 wks and 6 mo	Free AAs (µmol/L):<br><br>6 wks:<br>110.5 (54.9)<br>6 mo :<br>66.7 (27.4)	Weight, length, weight gain, length gain. (Not WHO)	(No) Association between valine intake or valine concentrations and infant outcomes<br><br>(-) Association between all (total) FAAs (free AAs) and infant weight gain at 6 wks	
**Non-essential amino acids**							
**Asparagine**							

Table S4. Characteristics and results of included studies reporting on human milk proteins and amino acids and infant anthropometrics - organized by component.

Authors, country, publication year (income setting)	Design and participants	Timing of milk sampling	Timing of infant anthropometrics	Estimated intake or HM concentration*	Anthropometric outcome measures and standards	Associations**	Major confounders considered
Baldeón Ecuador, 2019 (LMIC)	Longitudinal 65 enrolled 61 analyzed at 1 week 47 analyzed at 2 wks 38 analyzed at 2 mo 37 analyzed at 4 mo	1 week, 2 wks, 2 mo and 4 mo	1 week, 2 wks, 2 mo and 4 mo	Data not presented (concentrations in milk)<br><br>Automatic amino acid analyzer (model L-8900; Hitachi, Tokyo, Japan) for cation-exchange chromatography separation	Weight (children categorized by weight gain tertiles)<br>HC (children categorized by HC gain tertiles)	Difference between 1 week and 4 mo<br>(No) Association between asparagine and weight gain<br>(No) Association between asparagine and HC gain	Infant sex<br>Exclusive breastfeeding only
Reiderer et al. 2020 Austria (HIC)	Cohort<br><br>54 (47 analysed)	6-8 wks	14-16 wks	Amino Acid; median (IQR), µmol/l<br><br>14.40 (10.05)	Length. weight, body composition (FM, FFM), FMI< FFMI, BMI<br><br>(WHO)	No Associations reported for HM Amino Acids and infant anthropometry	BMI/Age ZScore, gestational weight gain, infant feeding mode (Fully BF, not fully BF)
Saben et al. 2021, USA (HIC)	Longitudinal (2 studies) 194 (normal weight, n= 68; OW, n=51; OB, n= 75)	0.5 mo<br>2 mo<br>6 mo	0.5 mo<br>2 mo<br>6 mo	0.5 mo (nmol/ml)<br>NW: 13.2 ± 1.4<br>OB: 11.5 ± 1.4<br>2 mo (nmol/ml)<br>NW: 19.0 ± 21.7<br>OB: 11.6 ± 1.4<br>6 mo (nmol/ml)<br>NW:23.0 ± 2.2<br>OB: 14.2 ± 2.2	Weight, length, Weight for GA, WAZ, WLZ, FMI, FFMI (WHO)	(No) Association between asparagine intake or asparagine concentrations and infant outcomes	Infant sex, body composition, birth weight
vanSadelhoff 2021, Germany (HIC)	Cohort<br><br>741 (441 analysed)	6 wks and 6 mo	6 wks and 6 mo	Free AAs (µmol/L):<br><br>6 wks:<br>25.7 (14.8)<br>6 mo :<br>17.7 (8.6)	Weight, length, weight gain, length gain. (Not WHO)	(No) Association between asparagine intake or asparagine concentrations and infant outcomes<br><br>(-) Association between all (total) FAAs (free AAs) and infant weight gain at 6 wks	
**Aspartic acid**							
Baldeón Ecuador, 2019 (LMIC)	Longitudinal 65 enrolled 61 analyzed at 1 week 47 analyzed at 2 wks 38 analyzed at 2 mo 37 analyzed at 4 mo	1 week, 2 wks, 2 mo and 4 mo	1 week, 2 wks, 2 mo and 4 mo	Data not presented (concentrations in milk)<br><br>Automatic amino acid analyzer (model L-8900; Hitachi, Tokyo, Japan) for cation-exchange chromatography separation	Weight (children categorized by weight gain tertiles)<br>HC (children categorized by HC gain tertiles)	Difference between 1 week and 4 mo<br>(No) Association between aspartic acid and weight gain<br>(No) Association between aspartic acid and HC gain	Infant sex<br>Exclusive breastfeeding only
Reiderer et al. 2020 Austria (HIC)	Cohort<br><br>54 (47 analysed)	6-8 wks	14-16 wks	Amino Acid; median (IQR), µmol/l<br><br>64.48 (48.36)	Length. weight, body composition (FM, FFM), FMI< FFMI, BMI<br><br>(WHO)	No Associations reported for HM Amino Acids and infant anthropometry	BMI/Age ZScore, gestational weight gain, infant feeding mode (Fully BF, not fully BF)
Saben et al. 2021, USA (HIC)	Longitudinal (2 studies) 194 (normal weight, n= 68; OW, n=51; OB, n= 75)	0.5 mo<br>2 mo<br>6 mo	0.5 mo<br>2 mo<br>6 mo	0.5 mo (nmol/ml)<br>NW: 44.7 ± 3.1<br>OB: 52.9 ± 3.3<br>2 mo (nmol/ml)<br>NW: 58.2 ± 4.2<br>OB: 87.4 ± 5.9<br>6 mo (nmol/ml)<br>NW:81.2 ± 7.0<br>OB: 104.3 ± 8.8	Weight, length, Weight for GA, WAZ WLZ, FMI, FFMI (WHO)	(+) Association between [Asp] and WLZ.<br>(+) Association between [Asp] and FMI.<br>(+) Association between [Asp] and FFMI.<br>(No) Associations between aspartic acid intake and infant outcomes	Infant sex, body composition, birth weight
vanSadelhoff 2021, Germany (HIC)	Cohort<br><br>741 (441 analysed)	6 wks and 6 mo	6 wks and 6 mo	Free AAs (µmol/L):<br><br>6 wks:<br>30.0 (17.4)<br>6 mo :<br>65.3 (39.1)	Weight, length, weight gain, length gain. (Not WHO)	(No) Associations between aspartic acid concentration or intake and infant outcomes<br><br>(-) Association between all (total) FAAs (free AAs) and infant weight gain at 6 wks	
**Cysteine**							
Baldeón Ecuador, 2019 (LMIC)	Longitudinal 65 enrolled 61 analyzed at 1 week 47 analyzed at 2 wks 38 analyzed at 2 mo 37 analyzed at 4 mo	1 week, 2 wks, 2 mo and 4 mo	1 week, 2 wks, 2 mo and 4 mo	Data not presented (concentrations in milk)<br><br>Automatic amino acid analyzer (model L-8900; Hitachi, Tokyo, Japan) for cation-exchange chromatography separation	Weight (children categorized by weight gain tertiles)<br>HC (children categorized by HC gain tertiles)	Difference between 1 week and 4 mo<br>(No) Association between cysteine and weight gain<br>(No) Association between cysteine and HC gain	Infant sex<br>Exclusive breastfeeding only

Table S4. Characteristics and results of included studies reporting on human milk proteins and amino acids and infant anthropometrics - organized by component.

Authors, country, publication year (income setting)	Design and participants	Timing of milk sampling	Timing of infant anthropometrics	Estimated intake or HM concentration*	Anthropometric outcome measures and standards	Associations**	Major confounders considered
Saben et al. 2021, USA (HIC)	Longitudinal (2 studies)<br>194 (normal weight, n= 68; OW, n=51; OB, n= 75)	0.5 mo<br>2 mo<br>6 mo	0.5 mo<br>2 mo<br>6 mo	0.5 mo (nmol/ml)<br>NW: 12.9 ± 0.7<br>OB: 10.2 ± 0.8 0<br>2 mo (nmol/ml)<br>NW: 21.6 ± 1.0<br>OB: 14.5 ± 0.8<br>6 mo (nmol/ml)<br>NW: 20.9 ± 1.1<br>OB: 19.7 ± 1.5	Weight, length, Weight for GA, WAZ, WLZ, FMI, FFMI (WHO)	(No) Association between cysteine intake and infant outcomes<br>(+) Association between [C-C] and WLZ<br>(+) Association between[C-C] and FMI.<br>(+) Association between [C-C] and FFMI.	Infant sex, body composition, birth weight
**Arginine**							
Baldeón<br>Ecuador, 2019<br>(LMIC)	Longitudinal<br>65 enrolled<br>61 analyzed at 1 week<br>47 analyzed at 2 wks<br>38 analyzed at 2 mo<br>37 analyzed at 4 mo	1 week, 2 wks, 2 mo and 4 mo	1 week, 2 wks, 2 mo and 4 mo	Data not presented	Weight (children categorized by weight gain tertiles)<br>HC (children categorized by HC gain tertiles)	Difference between 1 week and 4 mo<br>(No) Association between arginine and weight gain<br>(No) Association between arginine and HC gain	Infant sex<br>Exclusive breastfeeding only
Isganaitis et al.<br>USA, 2019<br>(HIC)	Longitudinal<br>37 enrolled<br>31 at 1 month<br>26 at 6 mo	1 and 6 mo	1 and 6 mo	Data not presented	Body composition (including fat mass %, fat accrual between 1-6 mo)<br>Weight	1 month timepoint<br>(-) Association between arginine and fat mass %<br>(-) Association between arginine and weight	Infant sex<br>Infant gestational age<br>Infant birthweight<br>Maternal parity
Reiderer et al. 2020 Austria (HIC)	Cohort<br><br>54 (47 analysed)	6-8 wks	14-16 wks	Amino Acid; median (IQR), µmol/l<br><br>6.32 (1.75)	Length. weight, body composition (FM, FFM), FMI< FFMI, BMI<br><br>(WHO)	No Associations reported for HM Amino Acids and infant anthropometry	BMI/Age ZScore, gestational weight gain, infant feeding mode (Fully BF, not fully BF)
vanSadelhoff 2021, Germany (HIC)	Cohort<br><br>741 (441 analysed)	6 wks and 6 mo	6 wks and 6 mo	Free AAs (µmol/L):<br><br>6 wks:<br>15.9 (9.5)<br>6 mo :<br>17.0 (6.4)	Weight, length, weight gain, length gain. (Not WHO)	(No) Associations between Arganine concentration or intake and infant outcomes<br><br>(-) Association between all (total) FAAs (free AAs) and infant weight gain at 6 wks	
**Glutamine**							
Baldeón<br>Ecuador, 2019<br>(LMIC)	Longitudinal<br>65 enrolled<br>61 analyzed at 1 week<br>47 analyzed at 2 wks<br>38 analyzed at 2 mo<br>37 analyzed at 4 mo	1 week, 2 wks, 2 mo and 4 mo	1 week, 2 wks, 2 mo and 4 mo	Data not presented (concentrations in milk)<br><br>Automatic amino acid analyzer (model L-8900; Hitachi, Tokyo, Japan) for cation-exchange chromatography separation	Weight (children categorized by weight gain tertiles)<br>HC (children categorized by HC gain tertiles)	Difference between 1 week and 4 mo<br>(No) Association between glutamine and weight gain<br>(No) Association between glutamine and HC gain	Infant sex<br>Exclusive breastfeeding only
Isganaitis et al.<br>USA, 2019<br>(HIC)	Longitudinal<br>37 enrolled<br>31 at 1 month<br>26 at 6 mo	1 and 6 mo	1 and 6 mo	Data not presented	Body composition (including fat mass %, fat accrual between 1-6 mo)<br>Weight	1 month timepoint<br>(+) Association between glutamine and fat mass %	Infant sex<br>Infant gestational age<br>Infant birthweight<br>Maternal parity
Larnkjaer et al.<br>Denmark, 2016<br>(HIC)	Longitudinal<br>78 enrolled<br>50 analyzed (exclusively breastfeeding)	4 mo	4 mo	Mean 1005 µmol/L (SD 242 µmol/L)	Weight<br>BMI<br>Length	(No) Association between glutamine and weight<br>(No) Association between glutamine and BMI<br>(+) Association between glutamine and length (not adjusted for birth length)<br>(No) Association between glutamine and length (adjusted for birth length)	Infant sex<br>Infant age<br>Infant birth anthropometry
Reiderer et al. 2020 Austria (HIC)	Cohort<br><br>54 (47 analysed)	6-8 wks	14-16 wks	Amino Acid; median (IQR), µmol/l<br><br>617.57 (431.77)	Length. weight, body composition (FM, FFM), FMI< FFMI, BMI<br><br>(WHO)	No Associations reported for HM Amino Acids and infant anthropometry	BMI/Age ZScore, gestational weight gain, infant feeding mode (Fully BF, not fully BF)
Saben et al. 2021, USA (HIC)	Longitudinal (2 studies)<br>194 (normal weight, n= 68; OW, n=51; OB, n= 75)	0.5 mo<br>2 mo<br>6 mo	0.5 mo<br>2 mo<br>6 mo	0.5 mo (nmol/ml)<br>NW: 108.6 ± 11.4<br>OB: 102.7 ± 10.9<br>2 mo (nmol/ml)<br>NW: 406.7 ± 21.5<br>OB: 286.2 ± 18.7<br>6 mo (nmol/ml)<br>NW: 535.1 ± 21.8<br>OB: 337.9 ± 26.3	Weight, length, Weight for GA, WAZ WLZ, FMI, FFMI (WHO)	(No) Association between glutamine intake and infant outcomes<br><br>(+) Association between [Gln] and FMI.<br>(+) Association between [Gln] and FFMI.	Infant sex, body composition, birth weight
vanSadelhoff 2021, Germany (HIC)	Cohort<br><br>741 (441 analysed)	6 wks and 6 mo	6 wks and 6 mo	Free AAs (µmol/L):<br><br>6 wks:<br>249.9 (162.9)<br>6 mo :<br>548.5 (268.6)	Weight, length, weight gain, length gain. (Not WHO)	(-) Association between glutamine and infant weight gain at 6 wks<br>(-) Association between Glutamine and infant length gain at 6 wks<br>(-) Association between all (total) FAAs (free AAs) and infant weight gain at 6 wks	

Table S4. Characteristics and results of included studies reporting on human milk proteins and amino acids and infant anthropometrics - organized by component.

Authors, country, publication year (income setting)	Design and participants	Timing of milk sampling	Timing of infant anthropometrics	Estimated intake or HM concentration*	Anthropometric outcome measures and standards	Associations**	Major confounders considered
**Glutamic acid**							
Baldeón<br>Ecuador, 2019<br>(LMIC)	Longitudinal<br>65 enrolled<br>61 analyzed at 1 week<br>47 analyzed at 2 wks<br>38 analyzed at 2 mo<br>37 analyzed at 4 mo	1 week, 2 wks, 2 mo and 4 mo	1 week, 2 wks, 2 mo and 4 mo	Data not presented<br><br>(concentrations in milk)<br><br>Automatic amino acid analyzer (model L-8900; Hitachi, Tokyo, Japan) for cation-exchange chromatography separation	Weight (infants categorized by weight gain tertiles)<br>HC (infants categorized by HC gain tertiles)	Difference between 1 week and 4 mo<br>(+) Association between glutamic acid and weight gain<br>(No) Association between glutamic acid and HC gain	Infant sex<br>Exclusive breastfeeding only
Larnkjaer et al.<br>Denmark, 2016<br>(HIC)	Longitudinal<br>78 enrolled<br>50 analyzed (exclusively breastfeeding)	4 mo	4 mo	Mean 262 μmol/L (SD 91 μmol/L)	Weight<br>BMI<br>Length	(No) Association between glutamic acid and weight<br>(No) Association between glutamic acid and BMI<br>(No) Association between glutamic acid and length	Infant sex<br>Infant age<br>Infant birth anthropometry
Reiderer et al. 2020<br>Austria (HIC)	Cohort<br><br>54 (47 analysed)	6-8 wks	14-16 wks	Amino Acid; median (IQR), μmol/l<br><br>1632.81 (418.04)	Length. weight, body composition (FM, FFM), FMI< FFMI, BMI<br><br>(WHO)	No Associations reported for HM Amino Acids and infant anthropometry	BMI/Age ZScore, gestational weight gain, infant feeding mode (Fully BF, not fully BF)
Saben et al. 2021, USA<br>(HIC)	Longitudinal (2 studies)<br>194 (normal weight, n= 68; OW, n=51; OB, n= 75)	0.5 mo<br>2 mo<br>6 mo	0.5 mo<br>2 mo<br>6 mo	0.5 mo (nmol/ml)<br>NW: 645.8 ± 25.4<br>OB: 606.3 ± 24.5<br>2 mo (nmol/ml)<br>NW: 851.5 ± 26.6<br>OB: 743.0 ± 23.5<br>6 mo (nmol/ml)<br>NW: 866.2 ± 24.8<br>OB: 789.9 ± 25.5	Weight, length, Weight for GA, WAZ, WLZ, FMI, FFMI (WHO)	(No) Association between glutamic acid intake and infant outcomes<br>(+) Association between [Glu] and WLZ<br>(+) Association between [Glu] and FMI.<br>(+) Association between [Glu] and FFMI.	Infant sex, body composition, birth weight
vanSadelhoff 2021,<br>Germany (HIC)	Cohort<br><br>741 (441 analysed)	6 wks and 6 mo	6 wks and 6 mo	Free AAs (μmol/L):<br><br>6 wks:<br>1270.8 (395.3)<br>6 mo :<br>1530.1 (328.5)	Weight, length, weight gain, length gain.<br>(Not WHO)	(-) Association between Glutamate and infant weight gain at 6 wks<br>(No) Association between glutamate and infant length or length gain<br><br>(-) Association between all (total) FAAs (free AAs) and infant weight gain at 6 wks	
**Alanine**							
Baldeón<br>Ecuador, 2019<br>(LMIC)	Longitudinal<br>65 enrolled<br>61 analyzed at 1 week<br>47 analyzed at 2 wks<br>38 analyzed at 2 mo<br>37 analyzed at 4 mo	1 week, 2 wks, 2 mo and 4 mo	1 week, 2 wks, 2 mo and 4 mo	Data not presented<br>(concentrations in milk)<br><br>Automatic amino acid analyzer (model L-8900; Hitachi, Tokyo, Japan) for cation-exchange chromatography separation	Weight (children categorized by weight gain tertiles)<br>HC (children categorized by HC gain tertiles)	Difference between 1 week and 4 mo<br>(+) Association between alanine and weight gain<br>(No) Association between alanine and HC gain	Infant sex<br>Exclusive breastfeeding only
Reiderer et al. 2020<br>Austria (HIC)	Cohort<br><br>54 (47 analysed)	6-8 wks	14-16 wks	Amino Acid; median (IQR), μmol/l<br><br>240.07 (81.50)	Length. weight, body composition (FM, FFM), FMI< FFMI, BMI<br><br>(WHO)	No Associations reported for HM Amino Acids and infant anthropometry	BMI/Age ZScore, gestational weight gain, infant feeding mode (Fully BF, not fully BF)
Saben et al. 2021, USA<br>(HIC)	Longitudinal (2 studies)<br>194 (normal weight, n= 68; OW, n=51; OB, n= 75)	0.5 mo<br>2 mo<br>6 mo	0.5 mo<br>2 mo<br>6 mo	0.5 mo (nmol/ml)<br>NW: 158.8 ± 8.3<br>OB: 165.4 ± 8.3<br>2 mo (nmol/ml)<br>NW: 188.6 ± 8.7<br>OB: 209.7 ± 10.6<br>6 mo (nmol/ml)<br>NW: 211.1 ± 8.2<br>OB: 210.9 ± 11.6	Weight, length, Weight for GA, WAZ, WLZ, FMI, FFMI (WHO)	(No) Association between alanine intake and infant outcomes	Infant sex, body composition, birth weight
vanSadelhoff 2021,<br>Germany (HIC)	Cohort<br><br>741 (441 analysed)	6 wks and 6 mo	6 wks and 6 mo	Free AAs (μmol/L):<br><br>6 wks:<br>228.2 (62.6)<br>6 mo :<br>242.7 (72.2)	Weight, length, weight gain, length gain.<br>(Not WHO)	(No) Association between alanine concentration or intake and infant outcomes<br><br>(-) Association between all (total) FAAs (free AAs) and infant weight gain at 6 wks	
**Glycine**							

Table S4. Characteristics and results of included studies reporting on human milk proteins and amino acids and infant anthropometrics - organized by component.

Authors, country, publication year (income setting)	Design and participants	Timing of milk sampling	Timing of infant anthropometrics	Estimated intake or HM concentration*	Anthropometric outcome measures and standards	Associations**	Major confounders considered
Baldeón<br>Ecuador, 2019<br>(LMIC)	Longitudinal<br>65 enrolled<br>61 analyzed at 1 week<br>47 analyzed at 2 wks<br>38 analyzed at 2 mo<br>37 analyzed at 4 mo	1 week, 2 wks, 2 mo and 4 mo	1 week, 2 wks, 2 mo and 4 mo	Data not presented<br>(concentrations in milk)<br><br>Automatic amino acid analyzer (model L-8900; Hitachi, Tokyo, Japan) for cation-exchange chromatography separation	Weight (children categorized by weight gain tertiles)<br>HC (children categorized by HC gain tertiles)	Difference between 1 week and 4 mo<br>(No) Association between glycine and weight gain<br>(No) Association between glycine and HC gain	Infant sex<br>Exclusive breastfeeding only
Isganaitis et al.<br>USA, 2019<br>(HIC)	Longitudinal<br>37 enrolled<br>31 at 1 month<br>26 at 6 mo	1 and 6 mo	1 and 6 mo	Data not presented	Body composition (including fat mass %, fat accrual between 1-6 mo)<br>Weight	6 mo timepoint<br>(-) Association between glycine and fat mass %	Infant sex<br>Infant gestational age<br>Infant birthweight<br>Maternal parity
Reiderer et al. 2020<br>Austria (HIC)	Cohort<br><br>54 (47 analysed)	6-8 wks	14-16 wks	Amino Acid; median (IQR), µmol/l<br><br>119.57 (53.32)	Length. weight, body composition (FM, FFM), FMI< FFMI, BMI<br><br>(WHO)	No Associations reported for HM Amino Acids and infant anthropometry	BMI/Age ZScore, gestational weight gain, infant feeding mode (Fully BF, not fully BF)
Saben et al. 2021, USA (HIC)	Longitudinal (2 studies)<br>194 (normal weight, n= 68; OW, n=51; OB, n= 75)	0.5 mo<br>2 mo<br>6 mo	0.5 mo<br>2 mo<br>6 mo	0.5 mo (nmol/ml)<br>NW: 70.6 ± 3.5<br>OB: 66.1 ± 3.2<br>2 mo (nmol/ml)<br>NW: 105.9 ± 4.9<br>OB: 86.5 ± 4.0<br>6 mo (nmol/ml)<br>NW: 126.5 ± 5.0<br>OB: 98.5 ± 5.6	Weight, length, Weight for GA, WAZ, WLZ, FMI, FFMI (WHO)	(No) Association between glycine intake and infant outcomes	Infant sex, body composition, birth weight
vanSadelhoff 2021, Germany (HIC)	Cohort<br><br>741 (441 analysed)	6 wks and 6 mo	6 wks and 6 mo	Free AAs (µmol/L):<br><br>6 wks:<br>138.7 (37.1)<br>6 mo :<br>146.3 (48.5)	Weight, length, weight gain, length gain. (Not WHO)	(No) Association between glycine concentration or intake and infant outcomes<br>(-) Association between all (total) FAAs (free AAs) and infant weight gain at 6 wks	
**Ornithine**							
Isganaitis et al.<br>USA, 2019<br>(HIC)	Longitudinal<br>37 enrolled<br>31 at 1 month<br>26 at 6 mo	1 and 6 mo	1 and 6 mo	Data not presented	Body composition (including fat mass %, fat accrual between 1-6 mo)<br>Weight	Difference between 1 and 6 mo<br>(+) Association between ornithine and fat accrual	Infant sex<br>Infant gestational age<br>Infant birthweight<br>Maternal parity
Reiderer et al. 2020<br>Austria (HIC)	Cohort<br><br>54 (47 analysed)	6-8 wks	14-16 wks	Amino Acid; median (IQR), µmol/l<br><br>2.96 (1.56)	Length. weight, body composition (FM, FFM), FMI< FFMI, BMI<br><br>(WHO)	No Associations reported for HM Amino Acids and infant anthropometry	BMI/Age ZScore, gestational weight gain, infant feeding mode (Fully BF, not fully BF)
vanSadelhoff 2021, Germany (HIC)	Cohort<br><br>741 (441 analysed)	6 wks and 6 mo	6 wks and 6 mo	Free AAs (µmol/L):<br><br>6 wks:<br>14.1 (15.1)<br>6 mo :<br>15.7 (27.0)	Weight, length, weight gain, length gain. (Not WHO)	(No) Associations between Ornithine concentration or intake and infant anthropometry<br><br>(-) Association between all (total) FAAs (free AAs) and infant weight gain at 6 wks	
**Proline**							
Baldeón<br>Ecuador, 2019<br>(LMIC)	Longitudinal<br>65 enrolled<br>61 analyzed at 1 week<br>47 analyzed at 2 wks<br>38 analyzed at 2 mo<br>37 analyzed at 4 mo	1 week, 2 wks, 2 mo and 4 mo	1 week, 2 wks, 2 mo and 4 mo	Data not presented<br><br>(concentrations in milk)<br><br>Automatic amino acid analyzer (model L-8900; Hitachi, Tokyo, Japan) for cation-exchange chromatography separation	Weight (children categorized by weight gain tertiles)<br>HC (children categorized by HC gain tertiles)	Difference between 1 week and 4 mo<br>(No) Association between proline and weight gain<br>(No) Association between proline and HC gain	Infant sex<br>Exclusive breastfeeding only
Isganaitis et al.<br>USA, 2019<br>(HIC)	Longitudinal<br>37 enrolled<br>31 at 1 month<br>26 at 6 mo	1 and 6 mo	1 and 6 mo	Data not presented	Body composition (including fat mass %, fat accrual between 1-6 mo)<br>Weight	1 month timepoint<br>(-) Association between proline and weight	Infant sex<br>Infant gestational age<br>Infant birthweight<br>Maternal parity
Reiderer et al. 2020<br>Austria (HIC)	Cohort<br><br>54 (47 analysed)	6-8 wks	14-16 wks	Amino Acid; median (IQR), µmol/l<br><br>28.36 (22.22)	Length. weight, body composition (FM, FFM), FMI< FFMI, BMI<br><br>(WHO)	No Associations reported for HM Amino Acids and infant anthropometry	BMI/Age ZScore, gestational weight gain, infant feeding mode (Fully BF, not fully BF)

Table S4. Characteristics and results of included studies reporting on human milk proteins and amino acids and infant anthropometrics - organized by component.

Authors, country, publication year (income setting)	Design and participants	Timing of milk sampling	Timing of infant anthropometrics	Estimated intake or HM concentration*	Anthropometric outcome measures and standards	Associations**	Major confounders considered
Saben et al. 2021, USA (HIC)	Longitudinal (2 studies)<br>194 (normal weight, n= 68; OW, n=51; OB, n= 75)	0.5 mo<br>2 mo<br>6 mo	0.5 mo<br>2 mo<br>6 mo	0.5 mo (nmol/ml)<br>NW: 44.4 ± 3.1<br>OB: 52.1 ± 3.2<br>2 mo (nmol/ml)<br>NW: 40.7 ± 1.9<br>OB: 42.0 ± 2.3<br>6 mo (nmol/ml)<br>NW: 36.0 ± 1.4<br>OB: 38.6 ± 1.9	Weight, length, Weight for GA, WAZ, WLZ, FMI, FFMI (WHO)	(No) Association between proline intake and infant outcomes	Infant sex, body composition, birth weight
**Tyrosine**							
Baldeón<br>Ecuador, 2019<br>(LMIC)	Longitudinal<br>65 enrolled<br>61 analyzed at 1 week<br>47 analyzed at 2 wks<br>38 analyzed at 2 mo<br>37 analyzed at 4 mo	1 week, 2 wks, 2 mo and 4 mo	1 week, 2 wks, 2 mo and 4 mo	Data not presented<br><br>(concentrations in milk)<br><br>Automatic amino acid analyzer (model L-8900; Hitachi, Tokyo, Japan) for cation-exchange chromatography separation	Weight (children categorized by weight gain tertiles)<br>HC (children categorized by HC gain tertiles)	Difference between 1 week and 4 mo<br>(No) Association between tyrosine and weight gain<br>(No) Association between tyrosine and HC gain	Infant sex<br>Exclusive breastfeeding only
Reiderer et al. 2020 Austria (HIC)	Cohort<br><br>54 (47 analysed)	6-8 wks	14-16 wks	Amino Acid; median (IQR), µmol/l<br><br>17.23 (6.77)	Length. weight, body composition (FM, FFM), FMI< FFMI, BMI<br><br>(WHO)	No Associations reported for HM Amino Acids and infant anthropometry	BMI/Age ZScore, gestational weight gain, infant feeding mode (Fully BF, not fully BF)
Saben et al. 2021, USA (HIC)	Longitudinal (2 studies)<br>194 (normal weight, n= 68; OW, n=51; OB, n= 75)	0.5 mo<br>2 mo<br>6 mo	0.5 mo<br>2 mo<br>6 mo	0.5 mo (nmol/ml)<br>NW: 13.6 ± 0.9<br>OB: 20.3 ± 1.4<br>2 mo (nmol/ml)<br>NW: 12.9 ± 0.9<br>OB: 18.9 ± 1.2<br>6 mo (nmol/ml)<br>NW:13.2 ± 0.9<br>OB: 17.3 ± 1.2	Weight, length, Weight for GA, WAZ, WLZ, FMI, FFMI (WHO)	(No) Association between tyrosine intake and infant outcomes	Infant sex, body composition, birth weight
vanSadelhoff 2021, Germany (HIC)	Cohort<br><br>741 (441 analysed)	6 wks and 6 mo	6 wks and 6 mo	Free AAs (µmol/L):<br><br>6 wks:<br>28.0 (21.0)<br>6 mo :<br>17.5 (7.8)	Weight, length, weight gain, length gain. (Not WHO)	(No) Association between tyrosine intake and infant outcomes<br><br>(-) Association between all (total) FAAs (free AAs) and infant weight gain at 6 wks	
**Serine**							
Baldeón<br>Ecuador, 2019<br>(LMIC)	Longitudinal<br>65 enrolled<br>61 analyzed at 1 week<br>47 analyzed at 2 wks<br>38 analyzed at 2 mo<br>37 analyzed at 4 mo	1 week, 2 wks, 2 mo and 4 mo	1 week, 2 wks, 2 mo and 4 mo	Data not presented<br><br>(concentrations in milk)<br><br>Automatic amino acid analyzer (model L-8900; Hitachi, Tokyo, Japan) for cation-exchange chromatography separation	Weight (children categorized by weight gain tertiles)<br>HC (children categorized by HC gain tertiles)	Difference between 1 week and 4 mo<br>(No) Association between serine and weight gain<br>(No) Association between serine and HC gain	Infant sex<br>Exclusive breastfeeding only
Reiderer et al. 2020 Austria (HIC)	Cohort<br><br>54 (47 analysed)	6-8 wks	14-16 wks	Amino Acid; median (IQR), µmol/l<br><br>113.59 (49.88)	Length. weight, body composition (FM, FFM), FMI< FFMI, BMI<br><br>(WHO)	No Associations reported for HM Amino Acids and infant anthropometry	BMI/Age ZScore, gestational weight gain, infant feeding mode (Fully BF, not fully BF)
Saben et al. 2021, USA (HIC)	Longitudinal (2 studies)<br>194 (normal weight, n= 68; OW, n=51; OB, n= 75)	0.5 mo<br>2 mo<br>6 mo	0.5 mo<br>2 mo<br>6 mo	0.5 mo (nmol/ml)<br>NW: 94.9 ± 4.7<br>OB: 94.9 ± 4.0<br>2 mo (nmol/ml)<br>NW: 147.3 ± 6.0<br>OB: 125.1 ± 4.7<br>6 mo (nmol/ml)<br>NW:171.8 ± 7.7<br>OB: 132.9 ± 7.0	Weight, length, Weight for GA, WAZ, WLZ, FMI, FFMI (WHO)	(No) Association between serine intake and infant outcomes<br><br>(+) Association between [Ser] and FMI.<br>(+) Association between [Ser] and FFMI.	Infant sex, body composition, birth weight
vanSadelhoff 2021, Germany (HIC)	Cohort<br><br>741 (441 analysed)	6 wks and 6 mo	6 wks and 6 mo	Free AAs (µmol/L):<br><br>6 wks:<br>100.0 (33.1)<br>6 mo :<br>137.8 (61.7)	Weight, length, weight gain, length gain. (Not WHO)	(-) Association between Serine and infant weight gain at 6 wks<br>(No) Association between Serine and infant length or gain<br>(-) Association between all (total) FAAs (free AAs) and infant weight gain at 6 wks	

*Values reported as mean ± SD or median (IQR). **"No (assumed) associations = unreported associations assumed to be no association.

Abbreviations: BF, breastfeeding; HIC, high income countries; mo, months; HM, human milk; LMIC, low and middle income countries; NCHS, National Center for Health Statistics; RCT, randomized controlled trial; SCM, subclinical mastitis; WHO, World Health Organization; wks, weeks

Anthropometrics: BMI, body mass indez; HAZ, height for age z-score; HC, head circumference; HCAZ, head circumference z-score; LAZ, length for age Z-score; LFA, length for age; WAZ, weight for age z-score; WFA, weight for age; WLZ, weight-for-length z-score

Table S5. Characteristics and results of included studies reporting on human milk fat and fatty acids and infant anthropometrics - organized by component.

Authors, country, publication year (income setting)	Design and participants	Timing of milk sampling	Timing of infant anthropometrics	Estimated intake or HM concentration*	Anthropometric outcome measures and standards	Associations**	Major confounders considered
**Fat**							
Abdelhamid et al. 2020, Egypt, (LMIC)	Cross-sectional<br><br>100	6-14 mo of age	6-14 mo of age	Fat (g/100 g)<br>4.58 ± 0.94<br><br>Gerber method	Weight and length, BMI	(-) Association between HM fat and infant BMI<br>(No) Association between HM fat and length or weight	None reported
Aksit et al.<br>Turkey, 2002<br>(LMIC)	Cross-sectional<br>80	2 mo	2 mo	Fat<br><br>Creamatocrit (% cream)	Weight (infants categorized by high or low weight gain)	Difference from birth to 2 mo<br>(-) Association between fat and weight gain<br><br>Mean 11.7% (SD 4.0%) at the end of breastfeeding in infants with higher weight gain; 14.3% (SD 3.8%) in infants with lower weight gain	None reported
deFluiter et al. 2021, Netherlands (HIC)	Cohort<br><br>133	1 and 3 mo	1, 3, 6, 9, 12, 18 and 24 mo	1 month timepoint<br>Fat (g/100 ml): 4.4 [3.3-5.8]<br><br>3 month timepoint<br>Fat (g/100 ml): 3.9 [2.6-5.6]<br><br>MIRIS human milk analyser	Weight, length, head circumference, WFL, WFA, HFA (SDs), FMI, Body composition Abdominal fat mass	3 month timepoint<br>(+) Association between HM fat (g/100 ml) and subcutaneous FM (cm) at 3 mo (beta 0.013 (0.002-0.025)<br>(+) Association between HM fat (g/100 ml) at 3 mo and change in FM% SDS from 1 to 6 mo (beta 0.088 (0.005-0.171), p = 0.039<br><br>6 month timepoint<br>(+) Association between HM fat at 3 mo and FM% at 6 mo (beta 0.387 (0.006-0.767) p < 0.049)	EBF during first 3 mo no other confounders reported
De Luca et al.<br>France, 2016<br>(HIC)	Longitudinal<br>165 enrolled<br>100 analyzed	1 month	1 month	Mean 3.4 g/dL (95% CI 3.0-3.7 g/dL) in normal weight mothers, 3.7 g/dL (95% CI 3.2-4.2 g/dL)<br><br>MIRIS human milk analyser	Weight<br>Length	1 month timepoint<br>(No) Association between fat and weight<br>(No) Association between fat and length<br><br>Difference from birth to 1 month<br>(+) Association between fat and weight gain<br>(No) Association between fat and length gain	Unadjusted estimates provided by authors<br><br>Exclusive breastfeeding
Dewey et al.<br>USA, 1993<br>(HIC)	Longitudinal<br>92 enrolled<br>46 analyzed	3, 6, 9, and 12 mo	Monthly from 1 to 18 mo then at 21 and 24 mo	3 mo timepoint<br>Mean 3.62 g/dL (SD 0.70 g/dL)<br><br>6 mo timepoint<br>Mean 3.77 g/dL (SD 0.96 g/dL)<br><br>9 mo timepoint<br>Mean 3.81 g/dL (SD 0.80 g/dL)<br><br>12 mo timepoint<br>Mean 3.72 g/dL (SD 1.13 g/dL)<br><br>Modified Folch extraction	WLZ<br>Skinfold thickness<br>Body composition (fat mass %)<br>NCHS reference standards	Any timepoint up to 24 mo<br>(No) Association between fat and WLZ<br>(No) Association between fat and skinfold thickness<br>(No) Association between fat and fat mass %	None reported
Dorea<br>Brazil, 1993<br>(LMIC)	Longitudinal<br>8	Bi-weekly or monthly between birth and 6 mo	Bi-weekly or monthly between birth and 6 mo	Data not presented<br><br>Association of Official Agricultural Chemists (A.O. A.C.)	Weight<br>Length	Difference from birth to 6 mo<br>(No) Association between fat and weight change<br>(No) Association between fat and length change	Multiple regression with zinc, total nitrogen, and fat
Ellsworth et al.<br>USA, 2020<br>(HIC)	Longitudinal<br>55 enrolled<br>32 analyzed	2 wks	2 wks and 2 mo	Mean 3.7 g/dL (SD 1.1 g/dL) in normal weight mothers, 3.5 g/dL (SD 0.9 g/dL) in mothers with overweight or obesity<br><br>MIRIS human milk analyser	WLZ<br>BMIZ<br>WAZ<br>LAZ<br>HC<br>WHO reference standards	Difference from 2 wks to 2 mo<br>(No) Association between fat and WLZ change<br>(No) Association between fat and BMIZ change<br>(+) Association between fat and WAZ change (exclusively breastfed infants only)<br>(No) Association between fat and LAZ change<br>(No) Association between fat and HC change	Infant sex
Fornes et al.<br>Brazil, 1995<br>(LMIC)	Longitudinal<br>39 enrolled	Bi-weekly intervals between 15 days and 3 mo	Bi-weekly intervals between 15 days and 3 mo	15 day timepoint<br>Mean 4.17 g/dL (SD 1.84 g/dL)<br><br>3 month timepoint<br>Mean 4.54 g/dL (SD 2.05 g/dL)<br><br>Creamatocrit	Weight<br>Length	(No) Association between fat and weight change<br>(No) Association between fat and length change	Exclusive breastfeeding
George et al. 2021, Australia<br>(HIC)	Cohort<br>30 enrolled, 18 analysed	Birth, 1, 2, 3, 4, 5, 6 mo postpartum	Birth, 1, 2, 3, 4, 5, 6 mo postpartum	Fatty Acids<br><br>(daily intake and concentration)<br><br>Fatty Acid Methyl Esters (FAME) and chromatography<br>Creamatocrit	Weight, length, head circumference, WFL Z Score, HC Z score, BMI	Monthly intake and growth (adjusted for multiple comparisons)<br>(+) Association between total lipids and HCZ, WLZ, weight, and BMI<br><br>Intake and growth at 6 mo (adjusted for multiple comparisons)(+) Association between total lipids and weight	None reported<br>Exclusive breastfeeding

Table S5. Characteristics and results of included studies reporting on human milk fat and fatty acids and infant anthropometrics - organized by component.

Authors, country, publication year (income setting)	Design and participants	Timing of milk sampling	Timing of infant anthropometrics	Estimated intake or HM concentration*	Anthropometric outcome measures and standards	Associations**	Major confounders considered
Kon et al.<br>Russia, 2014<br>(LMIC)	Longitudinal<br>103 enrolled<br>99 analyzed	1, 2, and 3 mo	1, 2, and 3 mo	1 month timepoint<br>Mean 4.08 g/dL (SEM 0.45 g/dL) for low weight gain group, 4.39 g/dL (SEM 0.84 g/dL) for normal weight gain group, 4.51 g/dL (SEM 0.57 g/dL) for high weight gain group<br><br>2 mo timepoint<br>Mean 4.43 g/dL (SEM 0.52 g/dL) for normal weight gain group, 3.65 g/dL (SEM 0.36 g/dL) for high weight gain group<br><br>6 mo timepoint<br>Mean 2.51 g/dL (SEM 0.26 g/dL) for low weight gain group, 3.76 g/dL (SEM 0.47 g/dL) for normal weight gain group, 3.80 g/dL (SEM 0.25 g/dL) for high weight gain group<br><br>Van de Kamer method	Weight gain (infants categorized by low, normal, and high weight gain)	1 month timepoint<br>(No) Association between fat and weight gain<br><br>2 mo timepoint<br>(No) Association between fat and weight gain<br><br>3 mo timepoint<br>(No) Association between fat and weight gain	None reported
Larsson et al.<br>Denmark, 2018<br>(HIC)	Longitudinal<br>59 enrolled<br>30 analyzed	5 mo	5 mo	Mean 3.02 g/dL (SD 1.59 g/dL) in the high weight gain group, 3.61 g/dL (SD 0.87 g/dL) in the normal weight group<br><br>MIRIS human milk analyser	BMIZ<br>WAZ<br>LAZ<br>WHO reference standards	5 mo timepoint<br>(No) Association between fat and BMIZ<br>(No) Association between fat and WAZ<br>(No) Association between fat and LAZ<br><br>Difference between birth and 5 mo<br>(No) Association between fat and change in BMIZ<br>(No) Association between fat and change in WAZ<br>(No) Association between fat and change in LAZ	None reported<br>Exclusive breastfeeding
Makela et al.<br>Finland, 2013<br>(HIC)	Longitudinal<br>100 enrolled<br>88 with anthropometry	3 mo	13 mo	Mean 3.13 g/dL (SD 1.57 g/dL) in overweight mothers, 3.00 g/dL (SD 1.08 g/dL) in normal weight mothers<br><br>Fatty Acid Methyl Esters (FAME) and chromatography<br>Creamatocrit	Weight<br>BMI<br>Length	13 mo timepoint<br>(No) Association between fat and weight<br>(No) Association between fat and BMI<br>(No) Association between fat and length<br><br>Difference between birth and 13 mo<br>(No) Association between fat and weight gain<br>(No) Association between fat and BMI gain	None
Martini et al.<br>Indonesia, 2020<br>(LMIC)	Longitudinal<br>40 enrolled<br>30 analyzed	1, 2, and 3 mo	1, 2, and 3 mo	1 month timepoint<br>Mean 3.41 g/dL (SD 0.97 g/dL)<br><br>2 mo timepoint<br>Mean 3.56 g/dL (SD 0.96 g/dL)<br><br>3 mo timepoint<br>Mean 2.80 g/dL (SD 1.16 g/dL)<br><br>MIRIS human milk analyser	Weight<br>Length<br>HC	1 month timepoint<br>(No) Association between fat and weight<br>(No) Association between fat and length<br>(No) Association between fat and HC<br><br>2 mo timepoint<br>(No) Association between fat and weight<br>(No) Association between fat and length<br>(No) Association between fat and HC<br><br>3 mo timepoint<br>(No) Association between fat and weight<br>(No) Association between fat and length<br>(No) Association between fat and HC	None reported
Minato et al.<br>Japan, 2019<br>(HIC)	Longitudinal<br>129 enrolled<br>88 analyzed at 1 month<br>56 analyzed at 3 mo	1 and 3 mo	1 and 3 mo	1 month timepoint<br>Median 3.9 g/dL (IQR 3.0, 4.9 g/dL) for lower weight gain infants, 3.7 g/dL (IQR 2.7, 4.9 g/dL) for normal weight gain infants<br><br>3 mo timepoint<br>Median 2.6 g/dL (IQR 1.9, 4.9 g/dL) for lower weight gain infants, 3.0 g/dL (IQR 2.1, 4.8 g/dL) for normal weight gain infants<br><br>MIRIS Human milk analyzer	Weight (infants categorized by lower or normal weight gain)	1 month timepoint<br>(No) Association between fat and weight<br><br>3 mo timepoint<br>(No) Association between fat and weight	Exclusive breastfeeding
Mitoulas et al.<br>Australia, 2002<br>(HIC)	Longitudinal<br>17	1, 2, 4, 6, 9, and 12 mo	6 mo	1-12 mo<br>Mean 3.74 g/dL (SE 0.06 g/dL)<br><br>(Estimated intake and concentration in HM)<br><br>Modified colorimetric spectrophotometric method (Stern & Shapiro)	Weight	Difference between birth and 6 mo<br>(No) Association between fat and weight gain	None reported
Nikniaz et al.<br>Iran, 2009<br>(LMIC)	Cross-sectional<br>182	3-4 mo	3-4 mo	Mean 3.52 g/dL (SD 1.41 g/dL)<br><br>Gerber method	WAZ<br>NCHS reference standards	(+) Association between fat and WAZ	Infant birthweight<br>Maternal BMI<br>Maternal age<br>Maternal energy intake
Palmer et al.<br>Zambia, 2016<br>(LMIC)	Randomized controlled trial<br>149 enrolled<br>145 analyzed	4-12 mo	4-12 mo	Mean 3.79 g/dL (SD 1.17 g/dL)<br><br>Creamatocrit	Weight<br>Length	(No) Association between fat and weight<br>(No) Association between fat and length	Unadjusted estimates provided by authors

Table S5. Characteristics and results of included studies reporting on human milk fat and fatty acids and infant anthropometrics - organized by component.

Authors, country, publication year (income setting)	Design and participants	Timing of milk sampling	Timing of infant anthropometrics	Estimated intake or HM concentration*	Anthropometric outcome measures and standards	Associations**	Major confounders considered
Prentice et al.<br>United Kingdom, 2019<br>(HIC)	Longitudinal<br>619 (subset of cohort)	4-8 wks	3, 12, and 24 mo	Median 2.6 g/dL (IQR 1.7-3.6 g/dL)<br><br>H-Nuclear magnetic resonance (NMR) spectra	Weight<br>BMI<br>Length<br>Body composition (skinfold thickness)	3 mo timepoint<br>(No) Association between fat and weight<br>(No) Association between fat and BMI<br>(No) Association between fat and length<br>(No) Association between fat and skinfold thickness<br><br>1 year timepoint<br>(No) Association between fat and weight<br>(-) Association between fat and BMI<br>(No) Association between fat and length<br>(-) Association between fat and skinfold thickness<br><br>Difference between 3 mo and 1 year<br>(-) Association between fat and weight increase<br>(-) Association between fat and BMI increase<br>(No) Association between fat and length increase<br>(-) Association between fat and skinfold thickness increase<br><br>2 year timepoint<br>(No) Association between fat and weight<br>(No) Association between fat and BMI<br>(No) Association between fat and length<br>(No) Association between fat and skinfold thickness<br><br>Difference between 1 and 2 years<br>(No) Association between fat and weight increase<br>(No) Association between fat and BMI increase<br>(No) Association between fat and length increase<br>(No) Association between fat and skinfold thickness increase	Infant sex<br>Infant birthweight<br>Infant gestational age<br>Exclusive breastfeeding<br>Duration of sample storage
Tyson et al.<br>USA, 1992<br>(HIC)	Longitudinal<br>40	2 and 6 wks	2 and 6 wks	Mean 4.85% (SD 1.60%) for the low fat yield group; 5.25% (1.85%) for the high fat yield group<br><br>Creamatocrit	Weight<br>Length<br>HC<br>Body composition (skinfold thickness)	Difference between birth and 2 wks<br>(No) Association between fat yield and weight gain<br><br>Difference between birth and 6 wks<br>(+) Association between fat yield and weight gain<br>(No) Association between fat yield and length gain<br>(No) Association between fat yield and change in head circumference<br><br>Difference between 2 and 6 wks<br>(No) Association between fat yield and weight gain<br>(No) Association between fat yield and length gain<br>(No) Association between fat yield and change in head circumference<br>(+) Association between fat yield and change in skinfold thickness	None reported
Ulloa et al. 2020,<br>Argentina, (HIC)	Cohort/Longitudina<br>l<br>22 (n=13, EWG;<br>n=9, AWG)	Protocol entry (4.34 [2.07 - 5.93] mo	Protocol entry and monthly thereafter until 1 year of age	No data reported<br><br>Gerber method	Weight, length, WFA, LFA, WFL (Z-Scores)	(No) Association between fat and excessive weight gain	Exclusive breastfeeding
Urteaga et al.<br>Bolivia, 2018<br>(LMIC)	Cross-sectional<br>18	2-6 mo	2-6 mo	Mean 4.11 g/dL (SD 1.29 g/dL)<br><br>Creamatocrit	WLZ<br>BMIZ<br>WAZ<br>LAZ<br>Body composition (fat mass and fat mass %)<br>WHO reference standards	(No) Association between fat and WLZ<br>(No) Association between fat and BMIZ<br>(No) Association between fat and WAZ<br>(No) Association between fat and LAZ<br>(No) Association between fat and fat mass<br>(No) Association between fat and fat mass %	Exclusive breastfeeding
**Hind milk**							
Larson-Meyer et al.<br>USA, 2020<br>(HIC)	Longitudinal<br>24 enrolled<br>22 analyzed at 1 month<br>15 analyzed at 6 mo	1 and 6 mo	1, 6, and 12 mo	1 month timepoint<br>Mean 13.0% (SD 15.1%)	Weight<br>WLZ<br>WAZ<br>WHO reference standards	1 month timepoint<br>(No) Association between hind milk fat and WLZ<br>(No) Association between hind milk fat and WAZ<br><br>Difference from birth to 1 month<br>(No) Association between hind milk fat and weight gain<br><br>6 mo timepoint<br>(No) Association between hind milk fat and WLZ<br>(No) Association between hind milk fat and WAZ<br><br>Difference from birth to 6 mo<br>(No) Association between hind milk fat and weight gain<br><br>12 mo timepoint<br>(No) Association between hind milk fat and WLZ<br>(No) Association between hind milk fat and WAZ<br><br>Difference from birth to 12 mo<br>(No) Association between hind milk fat and weight gain	Infant sex

Table S5. Characteristics and results of included studies reporting on human milk fat and fatty acids and infant anthropometrics - organized by component.

Authors, country, publication year (income setting)	Design and participants	Timing of milk sampling	Timing of infant anthropometrics	Estimated intake or HM concentration*	Anthropometric outcome measures and standards	Associations**	Major confounders considered
Miller et al.<br>USA, 2017<br>(HIC)	Cross-sectional<br>63	1-9 mo (mean 5.0, SD 2.4)	1-9 mo (mean 5.0, SD 2.4)	Mean 5.3% (SD 1.8%)	WAZ<br>LAZ<br>WHO reference standards	(No) Association between hind milk % fat and WAZ<br>(-) Association between hind milk % fat and LAZ	Infant sex<br>Infant age<br>Maternal age<br>Maternal BMI<br>Exclusive breastfeeding<br>Nursing session variables (session duration, time since last session, time of day session)
**Fore milk**							
Larson-Meyer et al.<br>USA, 2020<br>(HIC)	Longitudinal<br>24 enrolled<br>22 analyzed at 1 month<br>15 analyzed at 6 mo	1 and 6 mo	1, 6, and 12 mo	1 month timepoint<br>Mean 7.4% (SD 10.8%)	Weight<br>WLZ<br>WAZ<br>WHO reference standards	1 month timepoint<br>(No) Association between fore milk fat and WLZ<br>(+) Association between fore milk fat and WAZ<br><br>Difference from birth to 1 month<br>(No) Association between fore milk fat and weight gain<br><br>6 mo timepoint<br>(No) Association between fore milk fat and WLZ<br>(No) Association between fore milk fat and WAZ<br><br>Difference from birth to 6 mo<br>(No) Association between fore milk fat and weight gain<br><br>12 mo timepoint<br>(No) Association between fore milk fat and WLZ<br>(No) Association between fore milk fat and WAZ<br><br>Difference from birth to 12 mo<br>(No) Association between fore milk fat and weight gain	Infant sex
Miller et al.<br>USA, 2017<br>(HIC)	Cross-sectional<br>63	1-9 mo (mean 5.0, SD 2.4)	1-9 mo (mean 5.0, SD 2.4)	Mean 3.6% (SD 1.5%)	WAZ<br>LAZ<br>WHO reference standards	(No) Association between fore milk % fat and WAZ<br>(No) Association between fore milk % fat and LAZ	Infant sex<br>Infant age<br>Maternal age<br>Maternal BMI<br>Exclusive breastfeeding<br>Nursing session variables (session duration, time since last session, time of day session)
**Short-chain fatty acids**							
**Butyrate**							
Prentice et al.<br>United Kingdom, 2019<br>(HIC)	Longitudinal<br>619 (subset of cohort)	4-8 wks	3, 12, and 24 mo	Range 0-0.4 mM	Weight<br>BMI<br>Length<br>Body composition (skinfold thickness)	3 mo timepoint<br>(No) Association between butyrate and weight<br>(No) Association between butyrate and BMI<br>(No) Association between butyrate and length<br>(No) Association between butyrate and skinfold thickness<br><br>1 year timepoint<br>(No) Association between butyrate and weight<br>(-) Association between butyrate and BMI<br>(No) Association between butyrate and length<br>(-) Association between butyrate and skinfold thickness<br><br>Difference between 3 mo and 1 year<br>(-) Association between butyrate and weight increase<br>(-) Association between butyrate and BMI increase<br>(No) Association between butyrate and length increase<br>(No) Association between butyrate and skinfold thickness increase<br><br>2 year timepoint<br>(No) Association between butyrate and weight<br>(No) Association between butyrate and length<br>(No) Association between butyrate and BMI<br>(No) Association between butyrate and skinfold thickness<br><br>Difference between 1 and 2 years<br>(+) Association between butyrate and weight increase<br>(+) Association between butyrate and BMI increase<br>(No) Association between butyrate and length increase<br>(+) Association between butyrate and skinfold thickness increase	Infant sex<br>Infant birthweight<br>Infant gestational age<br>Exclusive breastfeeding<br>Duration of sample storage
**Formate**							

Table S5. Characteristics and results of included studies reporting on human milk fat and fatty acids and infant anthropometrics - organized by component.

Authors, country, publication year (income setting)	Design and participants	Timing of milk sampling	Timing of infant anthropometrics	Estimated intake or HM concentration*	Anthropometric outcome measures and standards	Associations**	Major confounders considered
Prentice et al.<br>United Kingdom, 2019<br>(HIC)	Longitudinal<br>619 (subset of cohort)	4-8 wks	3, 12, and 24 mo	Range 0.1-7.5 mM	Weight<br>BMI<br>Length<br>Body composition (skinfold thickness)	3 mo timepoint<br>(No) Association between formate and weight<br>(-) Association between formate and BMI<br>(No) Association between formate and length<br>(No) Association between formate and skinfold thickness<br><br>1 year timepoint<br>(No) Association between formate and weight<br>(-) Association between formate and BMI<br>(No) Association between formate and length<br>(No) Association between formate and skinfold thickness<br><br>Difference between 3 mo and 1 year<br>(No) Association between formate and weight increase<br>(No) Association between formate and BMI increase<br>(No) Association between formate and length increase<br>(No) Association between formate and skinfold thickness increase<br><br>2 year timepoint<br>(-) Association between formate and weight<br>(-) Association between formate and BMI<br>(No) Association between formate and length<br>(-) Association between formate and skinfold thickness<br><br>Difference between 1 and 2 years<br>(No) Association between formate and weight increase<br>(No) Association between formate and BMI increase<br>(No) Association between formate and length increase<br>(No) Association between formate and skinfold thickness increase	Infant sex<br>Infant birthweight<br>Infant gestational age<br>Exclusive breastfeeding<br>Duration of sample storage
**Acetate**							
Prentice et al.<br>United Kingdom, 2019<br>(HIC)	Longitudinal<br>619 (subset of cohort)	4-8 wks	3, 12, and 24 mo	Range 0.1-8.5 mM	Weight<br>Length<br>BMI<br>Skinfold thickness	3 mo timepoint<br>(No) Association between acetate and weight<br>(No) Association between acetate and BMI<br>(No) Association between acetate and length<br>(-) Association between acetate and skinfold thickness<br><br>1 year timepoint<br>(No) Association between acetate and weight<br>(No) Association between acetate and BMI<br>(No) Association between acetate and length<br>(No) Association between acetate and skinfold thickness<br><br>Difference between 3 mo and 1 year<br>(No) Association between acetate and weight increase<br>(No) Association between acetate and BMI increase<br>(No) Association between acetate and BMI increase<br>(No) Association between acetate and skinfold thickness increase<br><br>2 year timepoint<br>(No) Association between acetate and weight<br>(No) Association between acetate and BMI<br>(No) Association between acetate and length<br>(-) Association between acetate and skinfold thickness<br><br>Difference between 1 and 2 years<br>(No) Association between acetate and weight increase<br>(No) Association between acetate and BMI increase<br>(No) Association between acetate and length increase<br>(No) Association between acetate and skinfold thickness change	Infant sex<br>Infant birthweight<br>Infant gestational age<br>Exclusive breastfeeding<br>Duration of sample storage
**Saturated fatty acids**							
**10:0 (capric acid, CAP)**							
Jacobson et al.<br>Canada, 2008<br>(HIC)	Longitudinal<br>109 enrolled<br>74 analyzed at 6 mo<br>67 analyzed at 12 mo	6 mo and 1 year	6 mo and 1 year	Data not presented	Weight<br>Length<br>HC	6 mo timepoint<br>(No) Association between CAP and weight<br>(No) Association between CAP and length<br>(No) Association between CAP and HC<br><br>1 year timepoint<br>(-) Association between CAP and weight<br>(No) Association between CAP and length<br>(-) Association between CAP and HC	Unadjusted estimates provided by authors

Table S5. Characteristics and results of included studies reporting on human milk fat and fatty acids and infant anthropometrics - organized by component.

Authors, country, publication year (income setting)	Design and participants	Timing of milk sampling	Timing of infant anthropometrics	Estimated intake or HM concentration*	Anthropometric outcome measures and standards	Associations**	Major confounders considered
Miliku et al.<br>Canada, 2019<br>(HIC)	Longitudinal<br>1094 (subset of cohort)	3-4 mo	3 mo and 1 year	Mean 0.71% (SD 0.30%)	Weight<br>Length	3 month timepoint<br>(No) Association between CAP and weight<br>(No) Association between CAP and length<br><br>1 year timepoint<br>(No) Association between CAP and weight<br>(No) Association between CAP and length	Unadjusted estimates provided by authors
Mychaleckyj et al.<br>Bangladesh, 2020<br>(LMIC)	Longitudinal<br>700 enrolled<br>563 analyzed	3–43 days	6 wks, 1 year, and 2 years	Mean 1.16% (SD 0.54%)	WAZ<br>LAZ<br>WHO reference standards	Difference between 6 wks and 1 year<br>(No) Association between CAP and change in WAZ<br>(No) Association between CAP and change in LAZ<br><br>Difference between 6 wks and 2 years<br>(No) Association between CAP and change in WAZ<br>(No) Association between CAP and change in LAZ	Infant serum zinc<br>Infant sex<br>Infant age at the time of sample and data collection<br>Infant gestational age<br>Human milk AA and DHA, log(%AA) and log(%DHA)
Peng et al. 2021, China<br>(UMIC)	Longitudinal<br>101	1, 2 and 3 mo	1, 2 and 3 mo	Fatty Acids<br><br>(realtive abundance and ratios)	Weight, length, BMI, head circumference	(No) Associations reported.	none
**12:0 (lauric acid, LAU)**							
Jacobson et al.<br>Canada, 2008<br>(HIC)	Longitudinal<br>109 enrolled<br>74 analyzed at 6 mo<br>67 analyzed at 12 mo	6 mo and 1 year	6 mo and 1 year	Data not presented	Weight<br>Length<br>HC	6 mo timepoint<br>(No) Association between LAU and weight<br>(No) Association between LAU and length<br>(No) Association between LAU and HC<br><br>1 year timepoint<br>(No) Association between LAU and weight<br>(No) Association between LAU and length<br>(-) Association between LAU and HC	Unadjusted estimates provided by authors
Miliku et al.<br>Canada, 2019<br>(HIC)	Longitudinal<br>1094 (subset of cohort)	3-4 mo	3 mo and 1 year	Mean 4.80% (SD 1.57%)	Weight<br>Length	3 month timepoint<br>(No) Association between LAU and weight<br>(No) Association between LAU and length<br><br>1 year timepoint<br>(No) Association between LAU and weight<br>(No) Association between LAU and length	Unadjusted estimates provided by authors
Mychaleckyj et al.<br>Bangladesh, 2020<br>(LMIC)	Longitudinal<br>700 enrolled<br>563 analyzed	3–43 days	6 wks, 1 year, and 2 years	Mean 8.16% (SD 3.08%)	WAZ<br>LAZ<br>WHO reference standards	Difference between 6 wks and 1 year<br>(No) Association between LAU and change in WAZ<br>(No) Association between LAU and change in LAZ<br><br>Difference between 6 wks and 2 years<br>(No) Association between LAU and change in WAZ<br>(No) Association between LAU and change in LAZ	Infant serum zinc<br>Infant sex<br>Infant age at the time of sample and data collection<br>Infant gestational age<br>Human milk AA and DHA, log(%AA) and log(%DHA)
Peng et al. 2021, China<br>(UMIC)	Longitudinal<br>101	1, 2 and 3 mo	1, 2 and 3 mo	Fatty Acids<br><br>(realtive abundance and ratios)	Weight, length, BMI, head circumference	(No) Associations reported.	none
**14:0 (myristic acid, MYR)**							
George et al. 2021,<br>Australia<br>(HIC)	Cohort<br>30 enrolled, 18 analysed	Birth, 1, 2, 3, 4, 5, 6 mo postpartum	Birth, 1, 2, 3, 4, 5, 6 mo postpartum	Fatty Acids<br><br>(daily intake)	Weight, length, head circumference, WFL Z Score, HC Z score, BMI	Monthly intake and growth (adjusted for multiple comparisons)<br>(+) Association between myristoleic acid and HCZ, head circumference, weight, length and BMI<br><br>Intake and growth at 6 mo (adjusted for multiple comparisons)<br>(+) Association between myristoleic acid and HCZ, HC	None reported
Jacobson et al.<br>Canada, 2008<br>(HIC)	Longitudinal<br>109 enrolled<br>74 analyzed at 6 mo<br>67 analyzed at 12 mo	6 mo and 1 year	6 mo and 1 year	Data not presented	Weight<br>Length<br>HC	6 mo timepoint<br>(No) Association between MYR and weight<br>(No) Association between MYR and length<br>(No) Association between MYR and HC<br><br>1 year timepoint<br>(No) Association between MYR and weight<br>(No) Association between MYR and length<br>(-) Association between MYR and HC	Unadjusted estimates provided by authors
Miliku et al.<br>Canada, 2019<br>(HIC)	Longitudinal<br>1094 (subset of cohort)	3-4 mo	3 mo and 1 year	Mean 5.97% (SD 1.80%)	Weight<br>Length	3 month timepoint<br>(No) Association between MYR and weight<br>(No) Association between MYR and length<br><br>1 year timepoint<br>(No) Association between MYR and weight<br>(No) Association between MYR and length	Unadjusted estimates provided by authors

Table S5. Characteristics and results of included studies reporting on human milk fat and fatty acids and infant anthropometrics - organized by component.

Authors, country, publication year (income setting)	Design and participants	Timing of milk sampling	Timing of infant anthropometrics	Estimated intake or HM concentration*	Anthropometric outcome measures and standards	Associations**	Major confounders considered
Mychaleckyj et al. Bangladesh, 2020 (LMIC)	Longitudinal 700 enrolled 563 analyzed	3–43 days	6 wks, 1 year, and 2 years	Mean 8.04% (SD 3.13%)	WAZ LAZ WHO reference standards	Difference between 6 wks and 1 year<br>(No) Association between MYR and change in WAZ<br>(No) Association between MYR and change in LAZ<br><br>Difference between 6 wks and 2 years<br>(No) Association between MYR and change in WAZ<br>(No) Association between MYR and change in LAZ	Infant serum zinc<br>Infant sex<br>Infant age at the time of sample and data collection<br>Infant gestational age<br>Human milk AA and DHA, log(%AA) and log(%DHA)
Peng et al. 2021, China (UMIC)	Longitudinal 101	1, 2 and 3 mo	1, 2 and 3 mo	Fatty Acids<br><br>(realtive abundance and ratios)	Weight, length, BMI, head circumference	(No) Associations reported.	none
**14:1n-9 (tetradecanoic acid)**							
George et al. 2021, Australia (HIC)	Cohort 30 enrolled, 18 analysed	Birth, 1, 2, 3, 4, 5, 6 mo postpartum	Birth, 1, 2, 3, 4, 5, 6 mo postpartum	Fatty Acids<br><br>(daily intake)	Weight, length, head circumference, WFL Z Score, HC Z score, BMI	Monthly intake and growth (adjusted for multiple comparisons)<br>(+) Association between tetradecanoic acid and HCZ, WLZ, and BMI<br><br>Intake and growth at 6 mo (adjusted for multiple comparisons)<br>(+) Association between tetradecanoic acid and HCZ	None reported
Jacobson et al. Canada, 2008 (HIC)	Longitudinal 109 enrolled 74 analyzed at 6 mo 67 analyzed at 12 mo	6 mo and 1 year	6 mo and 1 year	Data not presented	Weight Length HC	6 mo timepoint<br>(No) Association between tetradecanoic acid and weight<br>(No) Association between tetradecanoic acid and length<br>(No) Association between tetradecanoic acidand HC<br><br>1 year timepoint<br>(No) Association between tetradecanoic acid and weight<br>(No) Association between tetradecanoic acid and length<br>(No) Association between tetradecanoic acid and HC	Unadjusted estimates provided by authors
Miliku et al. Canada, 2019 (HIC)	Longitudinal 1094 (subset of cohort)	3-4 mo	3 mo and 1 year	Mean 0.21% (SD 0.09%)	Weight Length	3 month timepoint<br>(No) Association between tetradecanoic acid and weight<br>(No) Association between tetradecanoic acid and length<br><br>1 year timepoint<br>(No) Association between tetradecanoic acid and weight<br>(No) Association between tetradecanoic acid and length	Unadjusted estimates provided by authors
Peng et al. 2021, China (UMIC)	Longitudinal 101	1, 2 and 3 mo	1, 2 and 3 mo	Fatty Acids<br><br>(realtive abundance and ratios)	Weight, length, BMI, head circumference	(No) Associations reported.	none
**15:0 (pentadecanoic acid)**							
George et al. 2021, Australia (HIC)	Cohort 30 enrolled, 18 analysed	Birth, 1, 2, 3, 4, 5, 6 mo postpartum	Birth, 1, 2, 3, 4, 5, 6 mo postpartum	Fatty Acids<br><br>(daily intake)	Weight, length, head circumference, WFL Z Score, HC Z score, BMI	Monthly intake and growth (adjusted for multiple comparisons)<br>(+) Association between pentadecanoic acid and HCZ, weight, length and BMI<br><br>Intake and growth at 6 mo (adjusted for multiple comparisons)<br>(+) Association between pentadecanoic acid and HCZ, HC	None reported
Jacobson et al. Canada, 2008 (HIC)	Longitudinal 109 enrolled 74 analyzed at 6 mo 67 analyzed at 12 mo	6 mo and 1 year	6 mo and 1 year	Data not presented	Weight Length HC	6 mo timepoint<br>(No) Association between pentadecanoic acid and weight<br>(No) Association between pentadecanoic acid and length<br>(No) Association between pentadecanoic acid and HC<br><br>1 year timepoint<br>(No) Association between pentadecanoic acid and weight<br>(No) Association between pentadecanoic acid and length<br>(No) Association between pentadecanoic acid and HC	Unadjusted estimates provided by authors
Miliku et al. Canada, 2019 (HIC)	Longitudinal 1094 (subset of cohort)	3-4 mo	3 mo and 1 year	Mean 0.31% (SD 0.10%)	Weight Length	3 month timepoint<br>(No) Association between pentadecanoic acid and weight<br>(No) Association between pentadecanoic acid and length<br><br>1 year timepoint<br>(No) Association between pentadecanoic acid and weight<br>(No) Association between pentadecanoic acid and length	Unadjusted estimates provided by authors
Peng et al. 2021, China (UMIC)	Longitudinal 101	1, 2 and 3 mo	1, 2 and 3 mo	Fatty Acids<br><br>(realtive abundance and ratios)	Weight, length, BMI, head circumference	(No) Associations reported.	none
**16:0 (palmitic acid, PAL)**							
George et al. 2021, Australia (HIC)	Cohort 30 enrolled, 18 analysed	Birth, 1, 2, 3, 4, 5, 6 mo postpartum	Birth, 1, 2, 3, 4, 5, 6 mo postpartum	Fatty Acids<br><br>(daily intake)	Weight, length, head circumference, WFL Z Score, HC Z score, BMI	Monthly intake and growth (adjusted for multiple comparisons)<br>(+) Association between palmitic acid and HCZ, WLZ, weight, and BMI<br><br>Intake and growth at 6 mo (adjusted for multiple comparisons)<br>(No) Association between palmitic acid and HCZ, HC, WLZ weight, length and BMI	None reported

Table S5. Characteristics and results of included studies reporting on human milk fat and fatty acids and infant anthropometrics - organized by component.

Authors, country, publication year (income setting)	Design and participants	Timing of milk sampling	Timing of infant anthropometrics	Estimated intake or HM concentration*	Anthropometric outcome measures and standards	Associations**	Major confounders considered
Jacobson et al.<br>Canada, 2008<br>(HIC)	Longitudinal<br>109 enrolled<br>74 analyzed at 6 mo<br>67 analyzed at 12 mo	6 mo and 1 year	6 mo and 1 year	Data not presented	Weight<br>Length<br>HC	6 mo timepoint<br>(No) Association between PAL and weight<br>(No) Association between PAL and length<br>(No) Association between PAL and HC<br><br>1 year timepoint<br>(No) Association between PAL and weight<br>(No) Association between PAL and length<br>(No) Association between PAL and HC	Unadjusted estimates provided by authors
Miliku et al.<br>Canada, 2019<br>(HIC)	Longitudinal<br>1094 (subset of cohort)	3-4 mo	3 mo and 1 year	Mean 20.90% (SD 2.76%)	Weight<br>Length	3 month timepoint<br>(No) Association between PAL and weight<br>(No) Association between PAL and length<br><br>1 year timepoint<br>(No) Association between PAL and weight<br>(No) Association between PAL and length	Unadjusted estimates provided by authors
Mychaleckyj et al.<br>Bangladesh, 2020<br>(LMIC)	Longitudinal<br>700 enrolled<br>563 analyzed	3–43 days	6 wks, 1 year, and 2 years	Mean 26.6% (SD 3.72%)	WAZ<br>LAZ<br>WHO reference standards	Difference between 6 wks and 1 year<br>(No) Association between PAL and change in WAZ<br>(No) Association between PAL and change in LAZ<br><br>Difference between 6 wks and 2 years<br>(No) Association between PAL and change in WAZ<br>(No) Association between PAL and change in LAZ	Infant serum zinc<br>Infant sex<br>Infant age at the time of sample and data collection<br>Infant gestational age<br>Human milk AA and DHA, log(%AA) and log(%DHA)
Peng et al. 2021, China (UMIC)	Longitudinal<br>101	1, 2 and 3 mo	1, 2 and 3 mo	Fatty Acids<br><br>(realtive abundance and ratios)	Weight, length, BMI, head circumference	(No) Associations reported.	none
**17:0 (heptadecanoic acid)**							
George et al. 2021,<br>Australia<br>(HIC)	Cohort<br>30 enrolled, 18 analysed	Birth, 1, 2, 3, 4, 5, 6 mo postpartum	Birth, 1, 2, 3, 4, 5, 6 mo postpartum	Fatty Acids<br><br>(daily intake)	Weight, length, head circumference, WFL Z Score, HC Z score, BMI	Monthly intake and growth (adjusted for multiple comparisons)<br>(+) Association between heptadecanoic acid acid and HCZ, WLZ, weight, and BMI<br><br>Intake and growth at 6 mo (adjusted for multiple comparisons)<br>(+) Association between heptadecanoic acid acid and HCZ,, weight,	None reported
Miliku et al.<br>Canada, 2019<br>(HIC)	Longitudinal<br>1094 (subset of cohort)	3-4 mo	3 mo and 1 year	Mean 0.31% (SD 0.08%)	Weight<br>Length	3 month timepoint<br>(No) Association between heptadecanoic acid and weight<br>(No) Association between heptadecanoic acid and length<br><br>1 year timepoint<br>(No) Association between heptadecanoic acid and weight<br>(No) Association between heptadecanoic acid and length	Unadjusted estimates provided by authors
Peng et al. 2021, China (UMIC)	Longitudinal<br>101	1, 2 and 3 mo	1, 2 and 3 mo	Fatty Acids<br><br>(realtive abundance and ratios)	Weight, length, BMI, head circumference	(No) Associations reported.	none
**18:0 (stearic acid, STE)**							
George et al. 2021,<br>Australia<br>(HIC)	Cohort<br>30 enrolled, 18 analysed	Birth, 1, 2, 3, 4, 5, 6 mo postpartum	Birth, 1, 2, 3, 4, 5, 6 mo postpartum	Fatty Acids<br><br>(daily intake)	Weight, length, head circumference, WFL Z Score, HC Z score, BMI	Monthly intake and growth (adjusted for multiple comparisons)<br>(No) Association between octadecanoic acid (18:0) and growth<br><br>Intake and growth at 6 mo (adjusted for multiple comparisons)<br>(+) Association between octadecanoic acid (18:0) and Weight	None reported
Jacobson et al.<br>Canada, 2008<br>(HIC)	Longitudinal<br>109 enrolled<br>74 analyzed at 6 mo<br>67 analyzed at 12 mo	6 mo and 1 year	6 mo and 1 year	Data not presented	Weight<br>Length<br>HC	6 mo timepoint<br>(No) Association between STE and weight<br>(No) Association between STE and length<br>(No) Association between STE and HC<br><br>1 year timepoint<br>(No) Association between STE and weight<br>(No) Association between STE and length<br>(No) Association between STE and HC	Unadjusted estimates provided by authors
Miliku et al.<br>Canada, 2019<br>(HIC)	Longitudinal<br>1094 (subset of cohort)	3-4 mo	3 mo and 1 year	Mean 0.31% (SD 0.08%)	Weight<br>Length	3 month timepoint<br>(No) Association between STE and weight<br>(No) Association between STE and length<br><br>1 year timepoint<br>(No) Association between STE and weight<br>(No) Association between STE and length	Unadjusted estimates provided by authors
Mychaleckyj et al.<br>Bangladesh, 2020<br>(LMIC)	Longitudinal<br>700 enrolled<br>563 analyzed	3–43 days	6 wks, 1 year, and 2 years	Mean 3.94% (SD 0.85%)	WAZ<br>LAZ<br>WHO reference standards	Difference between 6 wks and 1 year<br>(No) Association between STE and change in WAZ<br>(No) Association between STE and change in LAZ<br><br>Difference between 6 wks and 2 years<br>(No) Association between STE and change in WAZ<br>(No) Association between STE and change in LAZ	Infant serum zinc<br>Infant sex<br>Infant age at the time of sample and data collection<br>Infant gestational age<br>Human milk AA and DHA, log(%AA) and log(%DHA)

Table S5. Characteristics and results of included studies reporting on human milk fat and fatty acids and infant anthropometrics - organized by component.

Authors, country, publication year (income setting)	Design and participants	Timing of milk sampling	Timing of infant anthropometrics	Estimated intake or HM concentration*	Anthropometric outcome measures and standards	Associations**	Major confounders considered
Peng et al. 2021, China (UMIC)	Longitudinal 101	1, 2 and 3 mo	1, 2 and 3 mo	Fatty Acids<br><br>(realtive abundance and ratios)	Weight, length, BMI, head circumference	(-) Association between (SFA) C18:0 (2 month) and infant head circumference at 2 mo<br>(-) Association between (SFA) C18:0 (3 month) and infant head circumference at 3 mo	none
**20:0 (arachidic acid, ARA)**							
George et al. 2021, Australia (HIC)	Cohort 30 enrolled, 18 analysed	Birth, 1, 2, 3, 4, 5, 6 mo postpartum	Birth, 1, 2, 3, 4, 5, 6 mo postpartum	Fatty Acids<br><br>(daily intake)	Weight, length, head circumference, WFL Z Score, HC Z score, BMI	Monthly intake and growth (adjusted for multiple comparisons)<br>(No) Association between arachidic acid and HCZ, HC, WLZ weight, length and BMI<br><br>Intake and growth at 6 mo (adjusted for multiple comparisons)<br>(No) Association between arachidic acid and HCZ, HC, WLZ weight, length and BMI	None reported
Jacobson et al. Canada, 2008 (HIC)	Longitudinal 109 enrolled<br>74 analyzed at 6 mo<br>67 analyzed at 12 mo	6 mo and 1 year	6 mo and 1 year	Data not presented	Weight<br>Length<br>HC	6 mo timepoint<br>(No) Association between ARA and weight<br>(No) Association between ARA and length<br>(No) Association between ARA and HC<br><br>1 year timepoint<br>(No) Association between ARA and weight<br>(No) Association between ARA and length<br>(No) Association between ARA and HC	Unadjusted estimates provided by authors
Miliku et al. Canada, 2019 (HIC)	Longitudinal 1094 (subset of cohort)	3-4 mo	3 mo and 1 year	Mean 0.16% (SD 0.06%)	Weight<br>Length	3 month timepoint<br>(No) Association between ARA and weight<br>(No) Association between ARA and length<br><br>1 year timepoint<br>(No) Association between ARA and weight<br>(No) Association between ARA and length	Unadjusted estimates provided by authors
Mychaleckyj et al. Bangladesh, 2020 (LMIC)	Longitudinal 700 enrolled<br>563 analyzed	3–43 days	6 wks, 1 year, and 2 years	Mean 0.14% (SD 0.03%)	WAZ<br>LAZ<br>WHO reference standards	Difference between 6 wks and 1 year<br>(No) Association between ARA and change in WAZ<br>(No) Association between ARA and change in LAZ<br><br>Difference between 6 wks and 2 years<br>(No) Association between ARA and change in WAZ<br>(No) Association between ARA and change in LAZ	Infant serum zinc<br>Infant sex<br>Infant age at the time of sample and data collection<br>Infant gestational age<br>Human milk AA and DHA, log(%AA) and log(%DHA)
Peng et al. 2021, China (UMIC)	Longitudinal 101	1, 2 and 3 mo	1, 2 and 3 mo	Fatty Acids<br><br>(realtive abundance and ratios)	Weight, length, BMI, head circumference	(No) Associations reported.	none
**22:0 (behenic acid, BEH)**							
George et al. 2021, Australia (HIC)	Cohort 30 enrolled, 18 analysed	Birth, 1, 2, 3, 4, 5, 6 mo postpartum	Birth, 1, 2, 3, 4, 5, 6 mo postpartum	Fatty Acids<br><br>(daily intake)	Weight, length, head circumference, WFL Z Score, HC Z score, BMI	Monthly intake and growth (adjusted for multiple comparisons)<br>(No) Association between 22:0 and HCZ, HC, WLZ weight, length and BMI<br><br>Intake and growth at 6 mo (adjusted for multiple comparisons)<br>(No) Association between 22:0 and HCZ, HC, WLZ weight, length and BMI	None reported
Jacobson et al. Canada, 2008 (HIC)	Longitudinal 109 enrolled<br>74 analyzed at 6 mo<br>67 analyzed at 12 mo	6 mo and 1 year	6 mo and 1 year	Data not presented	Weight<br>Length<br>HC	6 mo timepoint<br>(No) Association between BEH and weight<br>(No) Association between BEH and length<br>(No) Association between BEH and HC<br><br>1 year timepoint<br>(No) Association between BEH and weight<br>(No) Association between BEH and length<br>(No) Association between BEH and HC	Unadjusted estimates provided by authors
Mychaleckyj et al. Bangladesh, 2020 (LMIC)	Longitudinal 700 enrolled<br>563 analyzed	3–43 days	6 wks, 1 year, and 2 years	Mean 0.07% (SD 0.02%)	WAZ<br>LAZ<br>WHO reference standards	Difference between 6 wks and 1 year<br>(No) Association between BEH and change in WAZ<br>(No) Association between BEH and change in LAZ<br><br>Difference between 6 wks and 1 year<br>(No) Association between BEH and change in WAZ<br>(No) Association between BEH and change in LAZ	Infant serum zinc<br>Infant sex<br>Infant age at the time of sample and data collection<br>Infant gestational age<br>Human milk AA and DHA, log(%AA) and log(%DHA)
Peng et al. 2021, China (UMIC)	Longitudinal 101	1, 2 and 3 mo	1, 2 and 3 mo	Fatty Acids<br><br>(realtive abundance and ratios)	Weight, length, BMI, head circumference	(No) Associations reported.	none
**24:0 (lignoceric acid, LIG)**							
George et al. 2021, Australia (HIC)	Cohort 30 enrolled, 18 analysed	Birth, 1, 2, 3, 4, 5, 6 mo postpartum	Birth, 1, 2, 3, 4, 5, 6 mo postpartum	Fatty Acids<br><br>(daily intake)	Weight, length, head circumference, WFL Z Score, HC Z score, BMI	Monthly intake and growth (adjusted for multiple comparisons)<br>(No) Association between tetracosanoic acid (24:0) and HCZ, HC, WLZ weight, length and BMI<br><br>Intake and growth at 6 mo (adjusted for multiple comparisons)<br>(No) Association between tetracosanoic acid (24:0) and HCZ, HC, WLZ weight, length and BMI	None reported

Table S5. Characteristics and results of included studies reporting on human milk fat and fatty acids and infant anthropometrics - organized by component.

Authors, country, publication year (income setting)	Design and participants	Timing of milk sampling	Timing of infant anthropometrics	Estimated intake or HM concentration*	Anthropometric outcome measures and standards	Associations**	Major confounders considered
Jacobson et al.<br>Canada, 2008<br>(HIC)	Longitudinal<br>109 enrolled<br>74 analyzed at 6 mo<br>67 analyzed at 12 mo	6 mo and 1 year	6 mo and 1 year	Data not presented	Weight<br>Length<br>HC	6 mo timepoint<br>(No) Association between LIG and weight<br>(No) Association between LIG and length<br>(No) Association between LIG and HC<br><br>1 year timepoint<br>(No) Association between LIG and weight<br>(No) Association between LIG and length<br>(No) Association between LIG and HC	Unadjusted estimates provided by authors
Mychaleckyj et al.<br>Bangladesh, 2020<br>(LMIC)	Longitudinal<br>700 enrolled<br>563 analyzed	3–43 days	6 wks, 1 year, and 2 years	Mean 0.08% (SD 0.03%)	WAZ<br>LAZ<br>WHO reference standards	Difference between 6 wks and 1 year<br>(No) Association between LIG and change in WAZ<br>(No) Association between LIG and change in LAZ<br><br>Difference between 6 wks and 2 years<br>(No) Association between LIG and change in WAZ<br>(No) Association between LIG and change in LAZ	Infant serum zinc<br>Infant sex<br>Infant age at the time of sample and data collection<br>Infant gestational age<br>Human milk AA and DHA, log(%AA) and log(%DHA)
Peng et al. 2021, China (UMIC)	Longitudinal<br>101	1, 2 and 3 mo	1, 2 and 3 mo	Fatty Acids<br><br>(realtive abundance and ratios)	Weight, length, BMI, head circumference	(No) Associations reported.	none
**Saturated fatty acids, SFAs**							
Makela et al.<br>Finland, 2013<br>(HIC)	Longitudinal<br>100 enrolled<br>88 with anthropometry	3 mo	13 mo	Mean 46.3% (SD 4.4%) in overweight mothers, 43.6% (SD 6.0%) in normal weight mothers	Weight<br>BMI<br>Length	13 mo timepoint<br>(No) Association between SFAs and weight<br>(No) Association between SFAs and BMI<br>(No) Association between SFAs and length<br><br>Difference between birth and 13 mo<br>(+) Association between SFAs and weight gain<br>(+) Association between SFAs and BMI gain	None reported
Peng et al. 2021, China (UMIC)	Longitudinal<br>101	1, 2 and 3 mo	1, 2 and 3 mo	Fatty Acids<br><br>(realtive abundance and ratios)	Weight, length, BMI, head circumference	(No) Associations reported.	none
**Monounsaturated fatty acids**							
**16:1n-7 (palmitoleic acid, PLE)**							
Jacobson et al.<br>Canada, 2008<br>(HIC)	Longitudinal<br>109 enrolled<br>74 analyzed at 6 mo<br>67 analyzed at 12 mo	6 mo and 1 year	6 mo and 1 year	Data not presented	Weight<br>Length<br>HC	6 mo timepoint<br>(No) Association between PLE and weight<br>(No) Association between PLE and length<br>(No) Association between PLE and HC<br><br>1 year timepoint<br>(No) Association between PLE and weight<br>(No) Association between PLE and length<br>(No) Association between PLE and HC	Unadjusted estimates provided by authors
Miliku et al.<br>Canada, 2019<br>(HIC)	Longitudinal<br>1094 (subset of cohort)	3-4 mo	3 mo and 1 year	Mean 2.69% (SD 0.68%)	Weight<br>Length	3 month timepoint<br>(-) Association between PLE and weight<br>(-) Association between PLE and length<br><br>1 year timepoint<br>(No) Association between PLE and weight<br>(No) Association between PLE and length	Unadjusted estimates provided by authors
Mychaleckyj et al.<br>Bangladesh, 2020<br>(LMIC)	Longitudinal<br>700 enrolled<br>563 analyzed	3–43 da ys	6 wks, 1 year, and 2 years	Mean 2.91% (SD 1.08%)	WAZ<br>LAZ<br>WHO reference standards	Difference between 6 wks and 1 year<br>(No) Association between PLE and change in LAZ<br>(No) Association between PLE and change in WAZ<br><br>Difference between 6 wks and 2 years<br>(No) Association between PLE and change in LAZ<br>(No) Association between PLE and change in WAZ	Infant serum zinc<br>Infant sex<br>Infant age at the time of sample and data collection<br>Infant gestational age<br>Human milk AA and DHA, log(%AA) and log(%DHA)
**18:1n-9 (oleic acid, OLE)**							
George et al. 2021, Australia<br>(HIC)	Cohort<br>30 enrolled, 18 analysed	Birth, 1, 2, 3, 4, 5, 6 mo postpartum	Birth, 1, 2, 3, 4, 5, 6 mo postpartum	Fatty Acids<br><br>(daily intake)	Weight, length, head circumference, WFL Z Score, HC Z score, BMI	Monthly intake and growth (adjusted for multiple comparisons)<br>(+) Association between 18:1n9 and HCZ, WLZ weight, and BMI<br><br>Intake and growth at 6 mo (adjusted for multiple comparisons)<br>(+) Association between 18:1n9 and weight,	None reported
Jacobson et al.<br>Canada, 2008<br>(HIC)	Longitudinal<br>109 enrolled<br>74 analyzed at 6 mo<br>67 analyzed at 12 mo	6 mo and 1 year	6 mo and 1 year	Data not presented	Weight<br>Length<br>HC	6 mo timepoint<br>(No) Association between OLE and weight<br>(No) Association between OLE and length<br>(No) Association between OLE and HC<br><br>1 year timepoint<br>(No) Association between OLE and weight<br>(No) Association between OLE and length<br>(No) Association between OLE and HC	Unadjusted estimates provided by authors

Table S5. Characteristics and results of included studies reporting on human milk fat and fatty acids and infant anthropometrics - organized by component.

Authors, country, publication year (income setting)	Design and participants	Timing of milk sampling	Timing of infant anthropometrics	Estimated intake or HM concentration*	Anthropometric outcome measures and standards	Associations**	Major confounders considered
Miliku et al.<br>Canada, 2019<br>(HIC)	Longitudinal<br>1094 (subset of cohort)	3-4 mo	3 mo and 1 year	Mean 37.05% (SD 3.59%)	Weight<br>Length	3 month timepoint<br>(No) Association between OLE and weight<br>(No) Association between OLE and length<br><br>1 year timepoint<br>(No) Association between OLE and weight<br>(No) Association between OLE and length	Unadjusted estimates provided by authors
Mychaleckyj et al.<br>Bangladesh, 2020<br>(LMIC)	Longitudinal<br>700 enrolled<br>563 analyzed	3–43 days	6 wks, 1 year, and 2 years	Mean 33.2% (SD 4.63%)	WAZ<br>LAZ<br>WHO reference standards	Difference between 6 wks and 1 year<br>(No) Association between OLE and change in LAZ<br>(No) Association between OLE and change in WAZ<br><br>Difference between 6 wks and 2 years<br>(No) Association between OLE and change in LAZ<br>(No) Association between OLE and change in WAZ	Infant serum zinc<br>Infant sex<br>Infant age at the time of sample and data collection<br>Infant gestational age<br>Human milk AA and DHA, log(%AA) and log(%DHA)
**20:1n-9 (eicosenoic acid, EIC)**							
George et al. 2021,<br>Australia<br>(HIC)	Cohort<br>30 enrolled, 18 analysed	Birth, 1, 2, 3, 4, 5, 6 mo postpartum	Birth, 1, 2, 3, 4, 5, 6 mo postpartum	Fatty Acids<br><br>(daily intake)	Weight, length, head circumference, WFL Z Score, HC Z score, BMI	Monthly intake and growth (adjusted for multiple comparisons)<br>(No) Association between cis-11,eicosenoic acid (24:0) and HCZ, HC, WLZ weight, length and BMI<br><br>Intake and growth at 6 mo (adjusted for multiple comparisons)<br>(+) Association between cis-11,eicosenoic acid and weight	None reported
Jacobson et al.<br>Canada, 2008<br>(HIC)	Longitudinal<br>109 enrolled<br>74 analyzed at 6 mo<br>67 analyzed at 12 mo	6 mo and 1 year	6 mo and 1 year	Data not presented	Weight<br>Length<br>HC	6 mo timepoint<br>(+) Association between EIC and weight<br>(No) Association between EIC and length<br>(+) Association between EIC and HC<br><br>1 year timepoint<br>(No) Association between EIC and weight<br>(No) Association between EIC and length<br>(No) Association between EIC and HC	Unadjusted estimates provided by authors
Mychaleckyj et al.<br>Bangladesh, 2020<br>(LMIC)	Longitudinal<br>700 enrolled<br>563 analyzed	3–43 days	6 wks, 1 year, and 2 years	Mean 0.42% (SD 0.16%)	WAZ<br>LAZ<br>WHO reference standards	Difference between 6 wks and 1 year<br>(No) Association between EIC and change in LAZ<br>(No) Association between EIC and change in WAZ<br><br>Difference between 6 wks and 2 years<br>(No) Association between EIC and change in LAZ<br>(No) Association between EIC and change in WAZ	Infant serum zinc<br>Infant sex<br>Infant age at the time of sample and data collection<br>Infant gestational age<br>Human milk AA and DHA, log(%AA) and log(%DHA)
**24:1n-9 (nervonic acid, NER)**							
George et al. 2021,<br>Australia<br>(HIC)	Cohort<br>30 enrolled, 18 analysed	Birth, 1, 2, 3, 4, 5, 6 mo postpartum	Birth, 1, 2, 3, 4, 5, 6 mo postpartum	Fatty Acids<br><br>(daily intake)	Weight, length, head circumference, WFL Z Score, HC Z score, BMI	Monthly intake and growth (adjusted for multiple comparisons)<br>(No) Association between cis-15-tetracosanoic acid (24:0) and HCZ, HC, WLZ weight, length and BMI<br><br>Intake and growth at 6 mo (adjusted for multiple comparisons)<br>(No) Association between cis-15-tetracosanoic acid (24:0) and HCZ, HC, WLZ weight, length and BMI	None reported
Jacobson et al.<br>Canada, 2008<br>(HIC)	Longitudinal<br>109 enrolled<br>74 analyzed at 6 mo<br>67 analyzed at 12 mo	6 mo and 1 year	6 mo and 1 year	Data not presented	Weight<br>Length<br>HC	6 mo timepoint<br>(No) Association between NER and weight<br>(No) Association between NER and length<br>(No) Association between NER and HC<br><br>1 year timepoint<br>(No) Association between NER and weight<br>(No) Association between NER and length<br>(No) Association between NER and HC	Unadjusted estimates provided by authors
Mychaleckyj et al.<br>Bangladesh, 2020<br>(LMIC)	Longitudinal<br>700 enrolled<br>563 analyzed	3–43 days	6 wks, 1 year, and 2 years	Mean 0.15% (SD 0.11%)	WAZ<br>LAZ<br>WHO reference standards	Difference between 6 wks and 1 year<br>(No) Association between NER and change in LAZ<br>(No) Association between NER and change in WAZ<br><br>Difference between 6 wks and 2 years<br>(No) Association between NER and change in LAZ<br>(No) Association between NER and change in WAZ	Infant serum zinc<br>Infant sex<br>Infant age at the time of sample and data collection<br>Infant gestational age<br>Human milk AA and DHA, log(%AA) and log(%DHA)
**18:1 c-11 (vacennic acid)**							
Miliku et al.<br>Canada, 2019<br>(HIC)	Longitudinal<br>1094 (subset of cohort)	3-4 mo	3 mo and 1 year	Mean 1.62% (SD 0.40%)	Weight<br>Length	3 month timepoint<br>(No) Association between vacennic acid and weight<br>(-) Association between vacennic acid and length<br><br>1 year timepoint<br>(No) Association between vacennic acid and weight<br>(-) Association between vacennic acid and length	Unadjusted estimates provided by authors
**Monounsaturated fatty acids, MUFAs**							

Table S5. Characteristics and results of included studies reporting on human milk fat and fatty acids and infant anthropometrics - organized by component.

Authors, country, publication year (income setting)	Design and participants	Timing of milk sampling	Timing of infant anthropometrics	Estimated intake or HM concentration*	Anthropometric outcome measures and standards	Associations**	Major confounders considered
Makela et al.<br>Finland, 2013<br>(HIC)	Longitudinal<br>100 enrolled<br>88 with anthropometry	3 mo	13 mo	Mean 38.8% (SD 3.6%) in overweight mothers, 40.1% (SD 4.0%) in normal weight mothers	Weight<br>BMI<br>Length	13 mo timepoint<br>(No) Association between MUFAs and weight<br>(No) Association between MUFAs and BMI<br>(No) Association between MUFAs and length<br><br>Difference between birth and 13 mo<br>(No) Association between total MUFAs and weight gain<br>(No) Association between total MUFAs and BMI gain	None
Peng et al. 2021, China<br>(UMIC)	Longitudinal<br>101	1, 2 and 3 mo	1, 2 and 3 mo	Fatty Acids<br><br>(realtive abundance and ratios)	Weight, length, BMI, head circumference	(No) Associations reported.	none
**Trans-fatty acids**							
**16:1n-7t (palmitelaidic acid, PLA)**							
Mychaleckyj et al.<br>Bangladesh, 2020<br>(LMIC)	Longitudinal<br>700 enrolled<br>563 analyzed	3–43 days	6 wks, 1 year, and 2 years	Mean 0.06% (SD 0.03%)	WAZ<br>LAZ<br>WHO reference standards	Difference between 6 wks and 1 year<br>(No) Association between PLA and change in LAZ<br>(No) Association between PLA and change in WAZ<br><br>Difference between 6 wks and 2 years<br>(No) Association between PLA and change in LAZ<br>(No) Association between PLA and change in WAZ	Infant serum zinc<br>Infant sex<br>Infant age at the time of sample and data collection<br>Infant gestational age<br>Human milk AA and DHA, log(%AA) and log(%DHA)
**18:1t (elaidic acid, ELA)**							
George et al. 2021,<br>Australia<br>(HIC)	Cohort<br>30 enrolled, 18 analysed	Birth, 1, 2, 3, 4, 5, 6 mo postpartum	Birth, 1, 2, 3, 4, 5, 6 mo postpartum	Fatty Acids<br><br>(daily intake)	Weight, length, head circumference, WFL Z Score, HC Z score, BMI	Monthly intake and growth (adjusted for multiple comparisons)<br>(+) Association between elaidic acid and HCZ, HC, weight, length and BMI<br><br>Intake and growth at 6 mo (adjusted for multiple comparisons)<br>(+) Association between elaidic acid and HCZ, HC	None reported
Mychaleckyj et al.<br>Bangladesh, 2020<br>(LMIC)	Longitudinal<br>700 enrolled<br>563 analyzed	3–43 days	6 wks, 1 year, and 2 years	Mean 0.36% (SD 0.26%)	WAZ<br>LAZ<br>WHO reference standards	Difference between 6 wks and 1 year<br>(No) Association between ELA and change in LAZ<br>(No) Association between ELA and change in WAZ<br><br>Difference between 6 wks and 2 years<br>(No) Association between ELA and change in LAZ<br>(No) Association between ELA and change in WA(Z	Infant serum zinc<br>Infant sex<br>Infant age at the time of sample and data collection<br>Infant gestational age<br>Human milk AA and DHA, log(%AA) and log(%DHA)
Peng et al. 2021, China<br>(UMIC)	Longitudinal<br>101	1, 2 and 3 mo	1, 2 and 3 mo	Fatty Acids<br><br>(realtive abundance and ratios)	Weight, length, BMI, head circumference	(No) Associations reported.	none
**18:1t-11 (trans-vaccenic acid, TVA)**							
Miliku et al.<br>Canada, 2019<br>(HIC)	Longitudinal<br>1094 (subset of cohort)	3-4 mo	3 mo and 1 year	Mean 1.44% (SD 1.08%)	Weight<br>Length	3 month timepoint<br>(No) Association between TVA and weight<br>(No) Association between TVA and length<br><br>1 year timepoint<br>(No) Association between TVA and weight<br>(No) Association between TVA and length	Unadjusted estimates provided by authors
**18:2n-6t (linoelaidic acid, LLA)**							
George et al. 2021,<br>Australia<br>(HIC)	Cohort<br>30 enrolled, 18 analysed	Birth, 1, 2, 3, 4, 5, 6 mo postpartum	Birth, 1, 2, 3, 4, 5, 6 mo postpartum	Fatty Acids<br><br>(daily intake)	Weight, length, head circumference, WFL Z Score, HC Z score, BMI	Monthly intake and growth (adjusted for multiple comparisons)<br>(No) Association between 18:2n-6t and HCZ, HC, WLZ weight, length and BMI<br><br>Intake and growth at 6 mo (adjusted for multiple comparisons)<br>(No) Association between 18:2n-6t and HCZ, HC, WLZ weight, length and BMI	None reported
Mychaleckyj et al.<br>Bangladesh, 2020<br>(LMIC)	Longitudinal<br>700 enrolled<br>563 analyzed	3–43 days	6 wks, 1 year, and 2 years	Mean 0.32% (SD 0.18%)	WAZ<br>LAZ<br>WHO reference standards	Difference between 6 wks and 1 year<br>(No) Association between LLA and change in LAZ<br>(No) Association between LLA and change in WAZ<br><br>Difference between 6 wks and 2 years<br>(No) Association between LLA and change in LAZ<br>(No) Association between LLA and change in WAZ	Infant serum zinc<br>Infant sex<br>Infant age at the time of sample and data collection<br>Infant gestational age<br>Human milk AA and DHA, log(%AA) and log(%DHA)
**Trans-fatty acids, Trans-FA**							
Peng et al. 2021, China<br>(UMIC)	Longitudinal<br>101	1, 2 and 3 mo	1, 2 and 3 mo	Fatty Acids<br><br>(realtive abundance and ratios)	Weight, length, BMI, head circumference	(No) Associations reported.	none
**Omega-3 polyunsaturated fatty acids**							
**18:3n-3 (alpha-linolenic acid, ALA)**							
Babiszewska<br>Poland, 2020<br>(HIC)	Cross-sectional<br>60	3-6 mo	3-6 mo	Mean 9.50% (SD 2.35%)<br><br>Fourier-transform infrared spectroscopy	HC<br>Head volume<br>Cranial indices (breadth/length, height/breadth, and height/length)	(No) Association between ALA and head circumference<br>(No) Association between ALA and head volume<br>(+) Association between ALA and cranial height/length ratio<br>(No) Association between ALA and cranial breadth/length ratio<br>(No) Association between ALA and cranial height/breadth ratio	Exclusive breastfeeding only<br>Infant sex<br>Infant age<br>Maternal socioeconomic status<br>Maternal cranial indices

Table S5. Characteristics and results of included studies reporting on human milk fat and fatty acids and infant anthropometrics - organized by component.

Authors, country, publication year (income setting)	Design and participants	Timing of milk sampling	Timing of infant anthropometrics	Estimated intake or HM concentration*	Anthropometric outcome measures and standards	Associations**	Major confounders considered
De la Garza Puentes et al.<br>Spain, 2019<br>(HIC)	Longitudinal<br>78 (subset of cohort)	2-4 days (colostrum) and 28-32 days (mature milk)	6 and 18 mo	Mean 0.59% (SD 0.21%) in normal weight mothers, 0.58% (SD 0.16%) in overweight mothers, 0.46% (0.08%) in mothers with obesity	BMIZ<br>WAZ<br>LAZ<br>WHO reference standards	6 mo timepoint (colostrum sample)<br>(No) Association between ALA and BMIZ<br>(No) Association between ALA and WAZ<br>(No) Association between ALA and LAZ<br><br>6 mo timepoint (mature milk sample)<br>(No) Association between ALA and BMIZ<br>(No) Association between ALA and WAZ<br>(No) Association between ALA and LAZ<br><br>18 mo timepoint (colostrum sample)<br>(No) Association between ALA and BMIZ<br>(No) Association between ALA and WAZ<br>(No) Association between ALA and LAZ<br><br>18 mo timepoint (mature milk sample)<br>(No) Association between ALA and BMIZ<br>(No) Association between ALA and WAZ<br>(No) Association between ALA and LAZ	Infant sex<br>Maternal BMI<br>Maternal weight gain during pregnancy<br>Maternal smoking<br>Maternal education<br>Infant feeding practices
George et al. 2021, Australia<br>(HIC)	Cohort<br>30 enrolled, 18 analysed	Birth, 1, 2, 3, 4, 5, 6 mo postpartum	Birth, 1, 2, 3, 4, 5, 6 mo postpartum	Fatty Acids<br><br>(daily intake)	Weight, length, head circumference, WFL Z Score, HC Z score, BMI	Monthly intake and growth (adjusted for multiple comparisons)<br>(+) Association between linolenic acid and HCZ<br><br>Intake and growth at 6 mo (adjusted for multiple comparisons)<br>(No) Association between linolenic acid and HCZ, HC, WLZ weight, length and BMI	None reported
Isganaitis et al.<br>USA, 2019<br>(HIC)	Longitudinal<br>37 enrolled<br>31 at 1 month<br>26 at 6 mo	1 and 6 mo	1 and 6 mo	Data not presented	Body composition (including fat mass %, fat accrual between 1-6 mo)<br>Weight	6 mo timepoint<br>(-) Association between LA or ALA and fat mass %	Infant sex<br>Infant gestational age<br>Infant birthweight<br>Maternal parity
Jacobson et al.<br>Canada, 2008<br>(HIC)	Longitudinal<br>109 enrolled<br>74 analyzed at 6 mo<br>67 analyzed at 12 mo	6 mo and 1 year	6 mo and 1 year	Data not presented	Weight<br>Length<br>HC	6 mo timepoint<br>(No) Association between ALA and weight<br>(No) Association between ALA and length<br>(+) Association between ALA and HC<br><br>1 year timepoint<br>(No) Association between ALA and weight<br>(No) Association between ALA and length<br>(+) Association between ALA and HC	Unadjusted estimates provided by authors
Miliku et al.<br>Canada, 2019<br>(HIC)	Longitudinal<br>1094 (subset of cohort)	3-4 mo	3 mo and 1 year	Mean 1.92% (SD 0.61%)	Weight<br>Length	3 month timepoint<br>(No) Association between ALA and weight<br>(No) Association between ALA and length<br><br>1 year timepoint<br>(No) Association between ALA and weight<br>(No) Association between ALA and length	Unadjusted estimates provided by authors
Mychaleckyj et al.<br>Bangladesh, 2020<br>(LMIC)	Longitudinal<br>700 enrolled<br>563 analyzed	3–43 days	6 wks, 1 year, and 2 years	Mean 0.54% (SD 0.40%)	WAZ<br>LAZ<br>WHO reference standards	Difference between 6 wks and 1 year<br>(No) Association between ALA and change in LAZ<br>(No) Association between ALA and change in WAZ<br><br>Difference between 6 wks and 2 years<br>(No) Association between ALA and change in LAZ<br>(No) Association between ALA and change in WAZ	Infant serum zinc<br>Infant sex<br>Infant age at the time of sample and data collection<br>Infant gestational age<br>Human milk AA and DHA, log(%AA) and log(%DHA)
Scholtens et al.<br>The Netherlands, 2009<br>(HIC)	Longitudinal<br>244 enrolled (subset of cohort)<br>177 analyzed	3-4 mo	1 year	Mean 1.016% (SD 0.375%)	Weight<br>BMI<br>Length	Difference between birth and 1 year<br>(No) Association between ALA and weight gain<br>(No) Association between ALA and BMI gain<br>(No) Association between ALA and length gain	Infant age<br>Breastfeeding duration<br>Low and high fatty acid tertiles<br>Sample collection time
Xiang et al.<br>Sweden, 2000<br>(LMIC)	Longitudinal<br>19	1 and 3 mo	1 and 3 mo	1 month timepoint<br>Mean 1.25% (SE 0.08%)<br><br>3 month timepoint<br>Mean 1.60% (SE 0.13%)	Occipito-frontal HC<br>Brain weight	Difference between birth and 1 month<br>(No) Association between ALA and occipito-frontal HC change<br>(No) Association between ALA and brain weight change<br><br>Difference between birth and 3 mo<br>(No) Association between ALA and occipito-frontal HC change<br>(No) Association between ALA and brain weight change	None reported
**20:4n-3 (eicosatetraenoic acid, ETA)**							
Jacobson et al.<br>Canada, 2008<br>(HIC)	Longitudinal<br>109 enrolled<br>74 analyzed at 6 mo<br>67 analyzed at 12 mo	6 mo and 1 year	6 mo and 1 year	Data not presented	Weight<br>Length<br>HC	6 mo timepoint<br>(No) Association between ETA and weight<br>(No) Association between ETA and length<br>(No) Association between ETA and HC<br><br>1 year timepoint<br>(No) Association between ETA and weight<br>(No) Association between ETA and length<br>(No) Association between ETA and HC	Unadjusted estimates provided by authors

Table S5. Characteristics and results of included studies reporting on human milk fat and fatty acids and infant anthropometrics - organized by component.

Authors, country, publication year (income setting)	Design and participants	Timing of milk sampling	Timing of infant anthropometrics	Estimated intake or HM concentration*	Anthropometric outcome measures and standards	Associations**	Major confounders considered
Miliku et al.<br>Canada, 2019<br>(HIC)	Longitudinal<br>1094 (subset of cohort)	3-4 mo	3 mo and 1 year	Mean 0.08% (SD 0.03%)	Weight<br>Length	3 month timepoint<br>(-) Association between ETA and weight<br>(-) Association between ETA and length<br><br>1 year timepoint<br>(No) Association between ETA and weight<br>(No) Association between ETA and length	Unadjusted estimates provided by authors
**20:5n-3 (eicosapentaenoic acid, EPA)**							
De la Garza Puentes et al.<br>Spain, 2019<br>(HIC)	Longitudinal<br>78 (subset of cohort)	2-4 days (colostrum) and 28-32 days (mature milk)	6, 18, and 36 mo	Mean 0.07% (SD 0.03%) in normal weight mothers, 0.06% (SD 0.02%) in overweight mothers, 0.07% (0.02%) in mothers with obesity	BMIZ<br>WAZ<br>LAZ<br>WHO reference standards	6 mo timepoint (colostrum sample)<br>(-) Association between EPA and BMIZ<br>(No) Association between EPA and WAZ<br>(No) Association between EPA and LAZ<br><br>6 mo timepoint (mature milk sample)<br>(No) Association between EPA and BMIZ<br>(No) Association between EPA and WAZ<br>(No) Association between EPA and LAZ<br><br>18 mo timepoint (colostrum sample)<br>(No) Association between EPA and BMIZ<br>(No) Association between EPA and WAZ<br>(No) Association between EPA and LAZ<br><br>18 mo timepoint (mature milk sample)<br>(No) Association between EPA and BMIZ<br>(No) Association between EPA and WAZ<br>(No) Association between EPA and LAZ	Infant sex<br>Maternal BMI<br>Maternal weight gain during pregnancy<br>Maternal smoking<br>Maternal education<br>Infant feeding practices
George et al. 2021,<br>Australia<br>(HIC)	Cohort<br>30 enrolled, 18 analysed	Birth, 1, 2, 3, 4, 5, 6 mo postpartum	Birth, 1, 2, 3, 4, 5, 6 mo postpartum	Fatty Acids<br><br>(daily intake)	Weight, length, head circumference, WFL Z Score, HC Z score, BMI	Monthly intake and growth (adjusted for multiple comparisons)<br>(No) Association between cis-5,8,11,14,17-eicosapentanoic acid and HCZ, HC, WLZ weight, length and BMI<br><br>Intake and growth at 6 mo (adjusted for multiple comparisons)<br>(No) Association between cis-5,8,11,14,17-eicosapentanoic acid and HCZ, HC, WLZ weight, length and BMI	None reported
Isganaitis et al.<br>USA, 2019<br>(HIC)	Longitudinal<br>37 enrolled<br>31 at 1 month<br>26 at 6 mo	1 and 6 mo	1 and 6 mo	Data not presented	Body composition (including fat mass %, fat accrual between 1-6 mo)<br>Weight	6 mo timepoint<br>(-) Association between EPA and fat mass %	Infant sex<br>Infant gestational age<br>Infant birthweight<br>Maternal parity
Jacobson et al.<br>Canada, 2008<br>(HIC)	Longitudinal<br>109 enrolled<br>74 analyzed at 6 mo<br>67 analyzed at 12 mo	6 mo and 1 year	6 mo nd 1 year	Data not presented	Weight<br>Length<br>HC	6 mo timepoint<br>(+) Association between EPA and weight<br>(+) Association between EPA and length<br>(+) Association between EPA and HC<br><br>1 year timepoint<br>(No) Association between EPA and weight<br>(No) Association between EPA and length<br>(No) Association between EPA and HC	Unadjusted estimates provided by authors
Miliku et al.<br>Canada, 2019<br>(HIC)	Longitudinal<br>1094 (subset of cohort)	3-4 mo	3 mo and 1 year	Mean 0.08% (SD 0.05%)	Weight<br>Length	3 month timepoint<br>(-) Association between EPA and weight<br>(No) Association between EPA and length<br><br>1 year timepoint<br>(No) Association between EPA and weight<br>(No) Association between EPA and length	Unadjusted estimates provided by authors

Table S5. Characteristics and results of included studies reporting on human milk fat and fatty acids and infant anthropometrics - organized by component.

Authors, country, publication year (income setting)	Design and participants	Timing of milk sampling	Timing of infant anthropometrics	Estimated intake or HM concentration*	Anthropometric outcome measures and standards	Associations**	Major confounders considered
Much et al.<br>Germany, 2013<br>(HIC)<br><br>Meyer et al.<br>Germany, 2019<br>(HIC)	Randomized controlled trial<br>208 infants enrolled<br>152 analyzed at 6 wks<br>120 analyzed at 4 mo	6 wks and 4 mo	6 wks, 4 mo 1 year, 2 years	6 week timepoint<br>Mean 0.08% (SD 0.04%) in control group, 0.18% (0.15%) in intervention group<br><br>4 month timepoint<br>Mean 0.07% (SD 0.04%) in control group, 0.15% (0.06%) in intervention group	Weight<br>BMI<br>Length<br>Body composition (skinfold thickness, fat mass, fat mass %, subcutaneous/preperitoneal fat, ponderal index)	6 wks timepoint (6 week sample)<br>(No) Association between EPA and weight<br>(No) Association between EPA and BMI<br>(No) Association between EPA and length<br>(No) Association between EPA and skinfold thickness<br>(No) Association between EPA and fat mass or fat mass %<br>(No) Association between EPA and subcutaneous/preperitoneal fat<br><br>4 mo timepoint (6 week sample)<br>(No) Association between EPA and weight<br>(No) Association between EPA and BMI<br>(No) Association between EPA and length<br>(No) Association between EPA and skinfold thickness<br>(No) Association between EPA and fat mass or fat mass %<br>(No) Association between EPA and subcutaneous/preperitoneal fat<br><br>4 mo timepoint (4 month sample)<br>(No) Association between EPA and weight<br>(No) Association between EPA and BMI<br>(-) Association between EPA and length<br>(No) Association between EPA and skinfold thickness<br>(No) Association between EPA and fat mass or fat mass %<br>(No) Association between EPA and subcutaneous/preperitoneal fat<br><br>1 year timepoint (6 week sample)<br>(No) Association between EPA and weight<br>(No) Association between EPA and BMI<br>(No) Association between EPA and length<br>(+) Association between EPA and skinfold thickness<br>(No) Association between EPA and fat mass or fat mass %<br>(No) Association between EPA and subcutaneous/preperitoneal fat<br><br>1 year timepoint (4 month sample)<br>(No) Association between EPA and weight<br>(No) Association between EPA and BMI<br>(-) Association between EPA and length<br>(No) Association between EPA and skinfold thickness<br>(No) Association between EPA and fat mass or fat mass %<br>(No) Association between EPA and subcutaneous/preperitoneal fat<br><br>2 year timepoint (6 week sample)<br>(No) Association between EPA and weight<br>(No) Association between EPA and BMI<br>(No) Association between EPA and length<br>(No) Association between EPA and skinfold thickness<br>(No) Association between EPA and fat mass or fat mass %<br>(No) Association between EPA and subcutaneous/preperitoneal fat<br><br>2 year timepoint (4 month sample)<br>(No) Association between EPA and weight<br>(No) Association between EPA and BMI<br>(No) Association between EPA and length<br>(No) Association between EPA and skinfold thickness<br>(No) Association between EPA and fat mass or fat mass %<br>(No) Association between EPA and subcutaneous/preperitoneal fat	Infant sex<br>Infant gestational age<br>Infant ponderal index at birth<br>Pregnancy duration<br>Maternal parity<br>Study group<br>Breastfeeding
Mychaleckyj et al.<br>Bangladesh, 2020<br>(LMIC)	Longitudinal<br>700 enrolled<br>563 analyzed	3–43 days	6 wks, 1 year, and 2 years	Mean 0.06% (SD 0.07%)	WAZ<br>LAZ<br>WHO reference standards	Difference between 6 wks and 1 year<br>(No) Association between EPA and change in LAZ<br>(No) Association between EPA and change in WAZ<br><br>Difference between 6 wks and 2 years<br>(No) Association between EPA and change in LAZ<br>(No) Association between EPA and change in WAZ	Infant serum zinc<br>Infant sex<br>Infant age at the time of sample and data collection<br>Infant gestational age<br>Human milk AA and DHA, log(%AA) and log(%DHA)
Peng et al. 2021, China (UMIC)	Longitudinal<br>101	1, 2 and 3 mo	1, 2 and 3 mo	Fatty Acids<br><br>(realtive abundance and ratios)	Weight, length, BMI, head circumference	(-) Association between (PUFA, n-3 profile) C20:5n3 (3 month) and infant head circumference at 3 mo<br>No other associations reported.	none
Scholtens et al.<br>The Netherlands, 2009<br>(HIC)	Longitudinal<br>244 enrolled<br>(subset of cohort)<br>177 analyzed	3-4 mo	1 year	Mean 0.050% (SD 0.042%)	Weight<br>BMI<br>Length	Difference between birth and 1 year<br>(No) Association between EPA and weight gain<br>(No) Association between EPA and BMI gain<br>(No) Association between EPA and length gain	Infant age<br>Breastfeeding duration<br>Low and high fatty acid tertiles<br>Sample collection time
**22:5n-3 (docosapentaenoic-n3 acid, DPA)**							

Table S5. Characteristics and results of included studies reporting on human milk fat and fatty acids and infant anthropometrics - organized by component.

Authors, country, publication year (income setting)	Design and participants	Timing of milk sampling	Timing of infant anthropometrics	Estimated intake or HM concentration*	Anthropometric outcome measures and standards	Associations**	Major confounders considered
Jacobson et al.<br>Canada, 2008<br>(HIC)	Longitudinal<br>109 enrolled<br>74 analyzed at 6 mo<br>67 analyzed at 12 mo	6 mo and 1 year	6 mo and 1 year	Data not presented	Weight<br>Length<br>HC	6 mo timepoint<br>(+) Association between DPA and weight<br>(+) Association between DPA and length<br>(+) Association between DPA and HC<br><br>1 year timepoint<br>(No) Association between DPA and weight<br>(No) Association between DPA and length<br>(No) Association between DPA and HC	Unadjusted estimates provided by authors
Miliku et al.<br>Canada, 2019<br>(HIC)	Longitudinal<br>1094 (subset of cohort)	3-4 mo	3 mo and 1 year	Mean 0.13% (SD 0.05%)	Weight<br>Length	3 month timepoint<br>(-) Association between DPA and weight<br>(No) Association between DPA and length<br><br>1 year timepoint<br>(No) Association between DPA and weight<br>(No) Association between DPA and length	Unadjusted estimates provided by authors
Mychaleckyj et al.<br>Bangladesh, 2020<br>(LMIC)	Longitudinal<br>700 enrolled<br>563 analyzed	3–43 days	6 wks, 1 year, and 2 years	Mean 0.14% (SD 0.08%)	WAZ<br>LAZ<br>WHO reference standards	Difference between 6 wks and 1 year<br>(No) Association between DPA and change in LAZ<br>(No) Association between DPA and change in WAZ<br><br>Difference between 6 wks and 2 years<br>(No) Association between DPA and change in LAZ<br>(No) Association between DPA and change in WAZ	Infant serum zinc<br>Infant sex<br>Infant age at the time of sample and data collection<br>Infant gestational age<br>Human milk AA and DHA, log(%AA) and log(%DHA)
Peng et al. 2021, China (UMIC)	Longitudinal<br>101	1, 2 and 3 mo	1, 2 and 3 mo	Fatty Acids<br><br>(realtive abundance and ratios)	Weight, length, BMI, head circumference	(No) Associations reported.	none
**22:6n-3 (docosahexanoic acid, DHA)**							
De la Garza Puentes et al.<br>Spain, 2019<br>(HIC)	Longitudinal<br>78 (subset of cohort)	2-4 days (colostrum) and 28-32 days (mature milk)	6 and 18 mo	Mean 0.28% (SD 0.11%) in normal weight mothers, 0.22% (SD 0.06%) in overweight mothers, 0.25% (0.07%) in mothers with obesity	BMIZ<br>WAZ<br>LAZ<br>WHO reference standards	6 mo timepoint (colostrum sample)<br>(-) Association between DHA and BMIZ<br>(No) Association between DHA and WAZ<br>(No) Association between DHA and LAZ<br><br>6 mo timepoint (mature milk sample)<br>(No) Association between DHA and BMIZ<br>(No) Association between DHA and WAZ<br>(No) Association between DHA and LAZ<br><br>18 mo timepoint (colostrum sample)<br>(No) Association between DHA and BMIZ<br>(No) Association between DHA and WAZ<br>(No) Association between DHA and LAZ<br><br>18 mo timepoint (mature milk sample)<br>(No) Association between DHA and BMIZ<br>(No) Association between DHA and WAZ<br>(No) Association between DHA and LAZ	Infant sex<br>Maternal BMI<br>Maternal weight gain during pregnancy<br>Maternal smoking<br>Maternal education<br>Infant feeding practices
George et al. 2021, Australia<br>(HIC)	Cohort<br>30 enrolled, 18 analysed	Birth, 1, 2, 3, 4, 5, 6 mo postpartum	Birth, 1, 2, 3, 4, 5, 6 mo postpartum	Fatty Acids<br><br>(daily intake)	Weight, length, head circumference, WFL Z Score, HC Z score, BMI	Monthly intake and growth (adjusted for multiple comparisons)<br>(No) Association between 4,7,10,13,16,19-docosahexaenoic acid and HCZ, HC, WLZ weight, length and BMI<br><br>Intake and growth at 6 mo (adjusted for multiple comparisons)<br>(No) Association between 4,7,10,13,16,19-docosahexaenoic acid and HCZ, HC, WLZ weight, length and BMI	None reported
Isganaitis et al.<br>USA, 2019<br>(HIC)	Longitudinal<br>37 enrolled<br>31 at 1 month<br>26 at 6 mo	1 and 6 mo	1 and 6 mo	Data not presented	Body composition (including fat mass %, fat accrual between 1-6 mo)<br>Weight	1 month timepoint<br>(-) Association between DHA and weight	Infant sex<br>Infant gestational age<br>Infant birthweight<br>Maternal parity
Jacobson et al.<br>Canada, 2008<br>(HIC)	Longitudinal<br>109 enrolled<br>74 analyzed at 6 mo<br>67 analyzed at 12 mo	6 mo and 1 year	6 mo and 1 year	Data not presented	Weight<br>Length<br>HC	6 mo timepoint<br>(No) Association between DHA and weight<br>(No) Association between DHA and length<br>(No) Association between DHA and HC<br><br>1 year timepoint<br>(No) Association between DHA and weight<br>(No) Association between DHA and length<br>(No) Association between DHA and HC	Unadjusted estimates provided by authors
Miliku et al.<br>Canada, 2019<br>(HIC)	Longitudinal<br>1094 (subset of cohort)	3-4 mo	3 mo and 1 year	Mean 0.18% (SD 0.12%)	Weight<br>Length	3 month timepoint<br>(-) Association between DHA and weight<br>(-) Association between DHA and length<br><br>1 year timepoint<br>(No) Association between DHA and weight<br>(No) Association between DHA and length	Unadjusted estimates provided by authors

Table S5. Characteristics and results of included studies reporting on human milk fat and fatty acids and infant anthropometrics - organized by component.

Authors, country, publication year (income setting)	Design and participants	Timing of milk sampling	Timing of infant anthropometrics	Estimated intake or HM concentration*	Anthropometric outcome measures and standards	Associations**	Major confounders considered
Much et al.<br>Germany, 2013<br>(HIC)<br><br>Meyer et al.<br>Germany, 2019<br>(HIC)	Randomized controlled trial<br>208 infants enrolled<br>152 analyzed at 6 wks<br>120 analyzed at 4 mo	6 wks and 4 mo	6 wks, 4 mo 1 year, 2 years	6 week timepoint<br>Mean 0.28% (SD 0.14%) in control group, 1.34% (0.67%) in intervention group<br><br>4 month timepoint<br>Mean 0.24% (SD 0.13%) in control group, 1.12% (0.39%) in intervention group	Weight<br>BMI<br>Length<br>Body composition (skinfold thickness, fat mass, fat mass %, subcutaneous/preperitoneal fat, ponderal index)	6 wks timepoint (6 week sample)<br>(No) Association between DHA and weight<br>(No) Association between DHA and BMI<br>(No) Association between DHA and length<br>(No) Association between DHA and skinfold thickness<br>(No) Association between DHA and fat mass or fat mass %<br>(+) Association between DHA and subcutaneous/preperitoneal fat<br><br>4 mo timepoint (6 week sample)<br>(No) Association between DHA and weight<br>(No) Association between DHA and BMI<br>(No) Association between DHA and length<br>(+) Association between DHA and skinfold thickness<br>(No) Association between DHA and fat mass<br>(+) Association between DHA and fat mass %<br>(No) Association between DHA and subcutaneous/preperitoneal fat<br><br>4 mo timepoint (4 month sample)<br>(No) Association between DHA and weight<br>(No) Association between DHA and BMI<br>(No) Association between DHA and length<br>(No) Association between DHA and skinfold thickness<br>(No) Association between DHA and fat mass or fat mass %<br>(No) Association between DHA and subcutaneous/preperitoneal fat<br><br>1 year timepoint (6 week sample)<br>(No) Association between DHA and weight<br>(No) Association between DHA and BMI<br>(No) Association between DHA and length<br>(+) Association between DHA and skinfold thickness<br>(No) Association between DHA and fat mass or fat mass %<br>(No) Association between DHA and subcutaneous/preperitoneal fat<br><br>1 year timepoint (4 month sample)<br>(No) Association between DHA and weight<br>(-) Association between DHA and BMI<br>(-) Association between DHA and length<br>(+) Association between DHA and skinfold thickness<br>(No) Association between DHA and fat mass or fat mass %<br>(No) Association between DHA and subcutaneous/preperitoneal fat<br><br>2 year timepoint (6 week sample)<br>(No) Association between DHA and weight<br>(+) Association between DHA and BMI<br>(No) Association between DHA and length<br>(No) Association between DHA and skinfold thickness<br>(No) Association between DHA and fat mass<br>(+) Association between DHA and fat mass %<br>(No) Association between DHA and subcutaneous/preperitoneal fat<br><br>2 year timepoint (4 month sample)<br>(No) Association between DHA and weight<br>(No) Association between DHA and BMI<br>(No) Association between DHA and length<br>(No) Association between DHA and skinfold thickness<br>(No) Association between DHA and fat mass or fat mass %<br>(No) Association between DHA and subcutaneous/preperitoneal fat	Infant sex<br>Infant gestational age<br>Infant ponderal index at birth<br>Maternal parity<br>Study group<br>Breastfeeding
Mychaleckyj et al.<br>Bangladesh, 2020<br>(LMIC)	Longitudinal<br>700 enrolled<br>563 analyzed	3–43 days	6 wks, 1 year, and 2 years	Mean 0.39% (SD 0.14%)	WAZ<br>LAZ<br>WHO reference standards	Difference between 6 wks and 1 year<br>(No) Association between DHA and change in LAZ<br>(No) Association between DHA and change in WAZ<br><br>Difference between 6 wks and 2 years<br>(No) Association between DHA and change in LAZ<br>(No) Association between DHA and change in WAZ	Infant serum zinc<br>Infant sex<br>Infant age at the time of sample and data collection<br>Infant gestational age<br>Human milk AA and DHA, log(%AA) and log(%DHA)
Peng et al. 2021, China (UMIC)	Longitudinal<br>101	1, 2 and 3 mo	1, 2 and 3 mo	Fatty Acids<br><br>(realtive abundance and ratios)	Weight, length, BMI, head circumference	(No) Associations reported.	none
Scholtens et al.<br>The Netherlands, 2009<br>(HIC)	Longitudinal<br>244 enrolled<br>(subset of cohort)<br>177 analyzed	3-4 mo	1 year	Mean 0.195% (SD 0.128%)	Weight<br>BMI<br>Length	Difference between birth and 1 year<br>(No) Association between DHA and weight gain<br>(No) Association between DHA and BMI gain<br>(No) Association between DHA and length gain	Infant age<br>Breastfeeding duration<br>Low and high fatty acid tertiles<br>Sample collection time

Table S5. Characteristics and results of included studies reporting on human milk fat and fatty acids and infant anthropometrics - organized by component.

Authors, country, publication year (income setting)	Design and participants	Timing of milk sampling	Timing of infant anthropometrics	Estimated intake or HM concentration*	Anthropometric outcome measures and standards	Associations**	Major confounders considered
Xiang et al.<br>China, 1999<br>(LMIC)	Cross-sectional<br>41 (18 infants 1 month old, 23 infants 3 mo old)	1 or 3 mo	1 or 3 mo	1 month timepoint<br>Mean 0.33% (SEM 0.05%)<br><br>3 month timepoint<br>Mean 0.18% (SEM 0.02%)	Weight<br>Length	Difference between birth and 1 month<br>(No) Association between DHA and weight gain<br>(+) Association between DHA and length gain<br><br>Difference between birth and 3 mo<br>(+) Association between DHA and weight gain<br>(+) Association between DHA and length gain	None
Xiang et al.<br>Sweden, 2000<br>(LMIC)	Longitudinal<br>19	1 and 3 mo	1 and 3 mo	1 month timepoint<br>Mean 0.28% (SE 0.02%)<br><br>3 month timepoint<br>Mean 0.25% (SE 0.01%)	Occipito-frontal HC<br>Brain weight	Difference between birth and 1 month<br>(No) Association between DHA and occipito-frontal HC change<br>(No) Association between DHA and brain weight change<br><br>Difference between birth and 3 mo<br>(No) Association between DHA and occipito-frontal HC change<br>(No) Association between DHA and brain weight change	None reported
**n-3 PUFAs**							
De la Garza Puentes et al.<br>Spain, 2019<br>(HIC)	Longitudinal<br>78 (subset of cohort)	2-4 days (colostrum) and 28-32 days (mature milk)	6 and 18 mo	Mean 1.05% (SD 0.24%) in normal weight mothers, 0.96% (SD 0.21%) in overweight mothers, 0.90% (0.13%) in mothers with obesity	BMIZ<br>WAZ<br>LAZ<br>WHO reference standards	6 mo timepoint (colostrum sample)<br>(-) Association between n-3 PUFAs and BMIZ<br>(No) Association between n-3 PUFAs and WAZ<br>(No) Association between n-3 PUFAs and LAZ<br><br>6 mo timepoint (mature milk sample)<br>(No) Association between n-3 PUFAs and BMIZ<br>(No) Association between n-3 PUFAs and WAZ<br>(No) Association between n-3 PUFAs and LAZ<br><br>18 mo timepoint (colostrum sample)<br>(No) Association between n-3 PUFAs and BMIZ<br>(No) Association between n-3 PUFAs and WAZ<br>(No) Association between n-3 PUFAs and LAZ<br><br>18 mo timepoint (mature milk sample)<br>(No) Association between n-3 PUFAs and BMIZ<br>(No) Association between n-3 PUFAs and WAZ<br>(No) Association between n-3 PUFAs and LAZ	Infant sex<br>Maternal BMI<br>Maternal weight gain during pregnancy<br>Maternal smoking<br>Maternal education<br>Infant feeding practices
Makela et al.<br>Finland, 2013<br>(HIC)	Longitudinal<br>100 enrolled<br>88 with anthropometry	3 mo	13 mo	Mean 2.2% (SD 0.79%) in overweight mothers, 2.7% (SD 1.1%) in normal weight mothers	Weight<br>BMI<br>Length	13 mo timepoint<br>(No) Association between n-3 PUFAs and weight<br>(No) Association between n-3 PUFAs and BMI<br>(No) Association between n-3 PUFAs and length<br><br>Difference between birth and 13 mo<br>(No) Association between n-3 PUFAs and weight gain<br>(No) Association between n-3 PUFAs and BMI gain	None
Nuss et al.<br>USA, 2019<br>(HIC)	Cross-sectional<br>33	4-8 wks	4-8 wks	Mean 1.4% (SD 0.5%)	Weight<br>Length<br>HC<br>Body composition (fat mass %)	(-) Association between total n-3 PUFAs and weight<br>(No) Association between total n-3 PUFAs and length<br>(+) Association between total n-3 PUFAs and HC<br>(+) Association between total n-3 PUFAs and % fat mass	Infant age
Peng et al. 2021, China (UMIC)	Longitudinal<br>101	1, 2 and 3 mo	1, 2 and 3 mo	Fatty Acids<br><br>(realtive abundance and ratios)	Weight, length, BMI, head circumference	(-) Association between (PUFA, n-3 profile) C20:3n3 (3 month) and infant head circumference at 3 mo<br>(-) Association between (PUFA, n-3 profile) C20:5n3 (3 month) and infant head circumference at 3 mo<br>No other associations reported.	none
Scholtens et al.<br>The Netherlands, 2009<br>(HIC)	Longitudinal<br>244 enrolled<br>(subset of cohort)<br>177 analyzed	3-4 mo	1 year	Mean 1.016% (SD 0.375%)	Weight<br>BMI<br>Length	Difference between birth and 1 year<br>(No) Association between n-3 PUFAs and weight gain<br>(No) Association between n-3 PUFAs and BMI gain<br>(No) Association between n-3 PUFAs and length gain	Infant age<br>Breastfeeding duration<br>Low and high fatty acid tertiles<br>Sample collection time
**Total n-3 LCPUFAs**							

Table S5. Characteristics and results of included studies reporting on human milk fat and fatty acids and infant anthropometrics - organized by component.

Authors, country, publication year (income setting)	Design and participants	Timing of milk sampling	Timing of infant anthropometrics	Estimated intake or HM concentration*	Anthropometric outcome measures and standards	Associations**	Major confounders considered
De la Garza Puentes et al.<br>Spain, 2019<br>(HIC)	Longitudinal<br>78 (subset of cohort)	2-4 days (colostrum) and 28-32 days (mature milk)	6 and 18 mo	Mean 0.45% (SD 0.16%) in normal weight mothers, 0.38% (SD 0.11%) in overweight mothers, 0.44% (0.09%) in mothers with obesity	BMIZ<br>WAZ<br>LAZ<br>WHO reference standards	6 mo timepoint (colostrum sample)<br>(-) Association between total n-3 LCPUFAs and BMIZ<br>(No) Association between total n-3 LCPUFAs and WAZ<br>(No) Association between total n-3 LCPUFAs and LAZ<br><br>6 mo timepoint (mature milk sample)<br>(No) Association between total n-3 LCPUFAs and BMIZ<br>(No) Association between total n-3 LCPUFAs and WAZ<br>(No) Association between total n-3 LCPUFAs and LAZ<br><br>18 mo timepoint (colostrum sample)<br>(No) Association between total n-3 LCPUFAs and BMIZ<br>(No) Association between total n-3 LCPUFAs and WAZ<br>(No) Association between total n-3 LCPUFAs and LAZ<br><br>18 mo timepoint (mature milk sample)<br>(No) Association between total n-3 LCPUFAs and BMIZ<br>(No) Association between total n-3 LCPUFAs and WAZ<br>(No) Association between total n-3 LCPUFAs and LAZ	Infant sex<br>Maternal BMI<br>Maternal weight gain during pregnancy<br>Maternal smoking<br>Maternal education<br>Infant feeding practices
Much et al.<br>Germany, 2013<br>(HIC)<br><br>Meyer et al.<br>Germany, 2019<br>(HIC)	Randomized controlled trial<br>208 infants enrolled<br>152 analyzed at 6 wks<br>120 analyzed at 4 mo	6 wks and 4 mo	6 wks, 4 mo 1 year, 2 years	6 week timepoint<br>Mean 0.66% (SD 0.25%) in control group, 1.94% (1.04%) in intervention group<br><br>4 month timepoint<br>Mean 0.59% (SD 0.21%) in control group, 1.61% (0.50%) in intervention group	Weight<br>BMI<br>Length<br>Body composition (skinfold thickness, fat mass, fat mass %, subcutaneous/preperitoneal fat, ponderal index)	6 wks timepoint (6 week sample)<br>(No) Association between total n-3 LCPUFAs and weight<br>(No) Association between total n-3 LCPUFAs and BMI<br>(No) Association between total n-3 LCPUFAs and length<br>(No) Association between total n-3 LCPUFAs and skinfold thickness<br>(No) Association between total n-3 LCPUFAs and fat mass or fat mass %<br>(+) Association between total n-3 LCPUFAs and subcutaneous/preperitoneal fat<br><br>4 mo timepoint (6 week sample)<br>(No) Association between total n-3 LCPUFAs and weight<br>(No) Association between total n-3 LCPUFAs and BMI<br>(No) Association between total n-3 LCPUFAs and length<br>(No) Association between total n-3 LCPUFAs and skinfold thickness<br>(No) Association between total n-3 LCPUFAs and fat mass or fat mass %<br>(No) Association between total n-3 LCPUFAs and subcutaneous/preperitoneal fat<br><br>4 mo timepoint (4 month sample)<br>(No) Association between total n-3 LCPUFAs and weight<br>(No) Association between total n-3 LCPUFAs and BMI<br>(-) Association between total n-3 LCPUFAs and length<br>(No) Association between total n-3 LCPUFAs and skinfold thickness<br>(No) Association between total n-3 LCPUFAs and fat mass or fat mass %<br>(No) Association between total n-3 LCPUFAs and subcutaneous/preperitoneal fat<br><br>1 year timepoint (6 week sample)<br>(No) Association between total n-3 LCPUFAs and weight<br>(No) Association between total n-3 LCPUFAs and BMI<br>(No) Association between total n-3 LCPUFAs and length<br>(+) Association between total n-3 LCPUFAs and skinfold thickness<br>(No) Association between total n-3 LCPUFAs and fat mass or fat mass %<br>(No) Association between total n-3 LCPUFAs and subcutaneous/preperitoneal fat<br><br>1 year timepoint (4 month sample)<br>(No) Association between total n-3 LCPUFAs and weight<br>(No) Association between total n-3 LCPUFAs and BMI<br>(-) Association between total n-3 LCPUFAs and length<br>(No) Association between total n-3 LCPUFAs and skinfold thickness<br>(No) Association between total n-3 LCPUFAs and fat mass or fat mass %<br>(No) Association between total n-3 LCPUFAs and subcutaneous/preperitoneal fat<br><br>2 year timepoint (6 week sample)<br>(+) Association between total n-3 LCPUFAs and weight<br>(+) Association between total n-3 LCPUFAs and BMI<br>(No) Association between total n-3 LCPUFAs and length<br>(+) Association between total n-3 LCPUFAs and skinfold thickness<br>(No) Association between total n-3 LCPUFAs and fat mass<br>(+) Association between total n-3 LCPUFAs and fat mass %<br>(No) Association between total n-3 LCPUFAs and subcutaneous/preperitoneal fat<br><br>2 year timepoint (4 month sample)<br>(No) Association between total n-3 LCPUFAs and weight<br>(No) Association between total n-3 LCPUFAs and BMI<br>(No) Association between total n-3 LCPUFAs and length<br>(No) Association between total n-3 LCPUFAs and skinfold thickness<br>(No) Association between total n-3 LCPUFAs and fat mass or fat mass %<br>(No) Association between total n-3 LCPUFAs and subcutaneous/preperitoneal fat	Infant sex<br>Infant gestational age<br>Infant ponderal index at birth<br>Maternal parity<br>Study group<br>Breastfeeding

Table S5. Characteristics and results of included studies reporting on human milk fat and fatty acids and infant anthropometrics - organized by component.

Authors, country, publication year (income setting)	Design and participants	Timing of milk sampling	Timing of infant anthropometrics	Estimated intake or HM concentration*	Anthropometric outcome measures and standards	Associations**	Major confounders considered
Scholtens et al.<br>The Netherlands, 2009<br>(HIC)	Longitudinal<br>244 enrolled<br>(subset of cohort)<br>177 analyzed	3-4 mo	1 year	Mean 0.514% (SD 0.210%)	Weight<br>BMI<br>Length	Difference between birth and 1 year<br>(No) Association between total n-3 LCPUFAs and weight gain<br>(No) Association between total n-3 LCPUFAs and BMI gain<br>(No) Association between total n-3 LCPUFAs and length gain	Infant age<br>Breastfeeding duration<br>Low and high fatty acid tertiles<br>Sample collection time
**Omega-6 polyunsaturated fatty acids**							
**18:2n-6 (linoleic acid, LA)**							
Babiszewska<br>Poland, 2020<br>(HIC)	Cross-sectional<br>60	3-6 mo	3-6 mo	Mean 9.50% (SD 2.35%)<br><br>Fourier-transform infrared spectroscopy	HC<br>Head volume<br>Cranial indices (breadth/length, height/breadth, and height/length)	(No) Association between LA and head circumference<br>(+) Association between LA and head volume<br>(No) Association between LA and cranial height/length ratio<br>(No) Association between LA and cranial breadth/length ratio<br>(No) Association between LA and cranial height/breadth ratio	Exclusive breastfeeding only<br>Infant sex<br>Infant age<br>Maternal socioeconomic status<br>Maternal cranial indices
De la Garza Puentes et al.<br>Spain, 2019<br>(HIC)	Longitudinal<br>78 (subset of cohort)	2-4 days (colostrum) and 28-32 days (mature milk)	6 and 18 mo	Mean 13.60% (SD 3.21%) in normal weight mothers, 15.20% (SD 3.86%) in overweight mothers, 13.90% (3.14%) in mothers with obesity	BMIZ<br>WAZ<br>LAZ<br>WHO reference standards	6 mo timepoint (colostrum sample)<br>(No) Association between LA and BMIZ<br>(+) Association between LA and WAZ<br>(No) Association between LA and LAZ<br><br>6 mo timepoint (mature milk sample)<br>(No) Association between LA and BMIZ<br>(No) Association between LA and WAZ<br>(No) Association between LA and LAZ<br><br>18 mo timepoint (colostrum sample)<br>(No) Association between LA and BMIZ<br>(No) Association between LA and WAZ<br>(No) Association between LA and LAZ<br><br>18 mo timepoint (mature milk sample)<br>(No) Association between LA and BMIZ<br>(No) Association between LA and WAZ<br>(No) Association between LA and LAZ	Infant sex<br>Maternal BMI<br>Maternal weight gain during pregnancy<br>Maternal smoking<br>Maternal education<br>Infant feeding practices
George et al. 2021, Australia<br>(HIC)	Cohort<br>30 enrolled, 18 analysed	Birth, 1, 2, 3, 4, 5, 6 mo postpartum	Birth, 1, 2, 3, 4, 5, 6 mo postpartum	Fatty Acids<br><br>(daily intake)	Weight, length, head circumference, WFL Z Score, HC Z score, BMI	Monthly intake and growth (adjusted for multiple comparisons)<br>(+) Association between 18:2n-6 and HCZ, HC, weight, length and BMI<br><br>Intake and growth at 6 mo (adjusted for multiple comparisons)<br>(+) Association between 18:2n-6 and HCZ, HC,	None reported
Isganaitis et al.<br>USA, 2019<br>(HIC)	Longitudinal<br>37 enrolled<br>31 at 1 month<br>26 at 6 mo	1 and 6 mo	1 and 6 mo	Data not presented	Body composition (including fat mass %, fat accrual between 1-6 mo)<br>Weight	6 mo timepoint<br>(-) Association between LA or ALA and fat mass %	Infant sex<br>Infant gestational age<br>Infant birthweight<br>Maternal parity
Jacobson et al.<br>Canada, 2008<br>(HIC)	Longitudinal<br>109 enrolled<br>74 analyzed at 6 mo<br>67 analyzed at 12 mo	6 mo and 1 year	6 mo and 1 year	Data not presented	Weight<br>Length<br>HC	6 mo timepoint<br>(No) Association between LA and weight<br>(No) Association between LA and length<br>(No) Association between LA and HC<br><br>1 year timepoint<br>(No) Association between LA and weight<br>(No) Association between LA and length<br>(No) Association between LA and HC	Unadjusted estimates provided by authors
Miliku et al.<br>Canada, 2019<br>(HIC)	Longitudinal<br>1094 (subset of cohort)	3-4 mo	3 mo and 1 year	Mean 13.62% (SD 3.01%)	Weight<br>Length	3 month timepoint<br>(No) Association between LA and weight<br>(No) Association between LA and length<br><br>1 year timepoint<br>(No) Association between LA and weight<br>(No) Association between LA and length	Unadjusted estimates provided by authors
Mychaleckyj et al.<br>Bangladesh, 2020<br>(LMIC)	Longitudinal<br>700 enrolled<br>563 analyzed	3–43 days	6 wks, 1 year, and 2 years	Mean 11.3% (SD 5.03%)	WAZ<br>LAZ<br>WHO reference standards	Difference between 6 wks and 1 year<br>(No) Association between LA and change in LAZ<br>(No) Association between LA and change in WAZ<br><br>Difference between 6 wks and 2 years<br>(No) Association between LA and change in LAZ<br>(No) Association between LA and change in WAZ	Infant serum zinc<br>Infant sex<br>Infant age at the time of sample and data collection<br>Infant gestational age<br>Human milk AA and DHA, log(%AA) and log(%DHA)
Peng et al. 2021, China<br>(UMIC)	Longitudinal<br>101	1, 2 and 3 mo	1, 2 and 3 mo	Fatty Acids<br><br>(realtive abundance and ratios)	Weight, length, BMI, head circumference	(No) Associations reported.	none
Scholtens et al.<br>The Netherlands, 2009<br>(HIC)	Longitudinal<br>244 enrolled<br>(subset of cohort)<br>177 analyzed	3-4 mo	1 year	Mean 15.07% (SD 4.09%)	Weight<br>BMI<br>Length	Difference between birth and 1 year<br>(-) Association between LA and weight gain<br>(-) Association between LA and BMI gain (high tertile only)<br>(No) Association between LA and length gain	Infant age<br>Breastfeeding duration<br>Low and high fatty acid tertiles<br>Sample collection time

Table S5. Characteristics and results of included studies reporting on human milk fat and fatty acids and infant anthropometrics - organized by component.

Authors, country, publication year (income setting)	Design and participants	Timing of milk sampling	Timing of infant anthropometrics	Estimated intake or HM concentration*	Anthropometric outcome measures and standards	Associations**	Major confounders considered
Xiang et al.<br>Sweden, 2000<br>(LMIC)	Longitudinal<br>19	1 and 3 mo	1 and 3 mo	1 month timepoint<br>Mean 10.29% (SE 0.51%)<br><br>3 month timepoint<br>Mean 10.93% (SE 0.45%)	Occipito-frontal head circumference<br>Brain weight	Difference between birth and 1 month<br>(No) Association between LA and occipito-frontal HC change<br>(No) Association between LA and brain weight change<br><br>Difference between birth and 3 mo<br>(No) Association between LA and occipito-frontal HC change<br>(No) Association between LA and brain weight change	None reported
**18:3n-6 (gamma-linolenic acid, GLA)**							
George et al. 2021,<br>Australia<br>(HIC)	Cohort<br>30 enrolled, 18 analysed	Birth, 1, 2, 3, 4, 5, 6 mo postpartum	Birth, 1, 2, 3, 4, 5, 6 mo postpartum	Fatty Acids<br><br>(daily intake)	Weight, length, head circumference, WFL Z Score, HC Z score, BMI	Monthly intake and growth (adjusted for multiple comparisons)<br>(No) Association between Y-linoleic acid and HCZ, HC, WLZ weight, length and BMI<br><br>Intake and growth at 6 mo (adjusted for multiple comparisons)<br>(No) Association between Y-linoleic acid and HCZ, HC, WLZ weight, length and BMI	None reported
Jacobson et al.<br>Canada, 2008<br>(HIC)	Longitudinal<br>109 enrolled<br>74 analyzed at 6 mo<br>67 analyzed at 12 mo	6 mo and 1 year	6 mo and 1 year	Data not presented	Weight<br>Length<br>HC	6 mo timepoint<br>(No) Association between GLA and weight<br>(No) Association between GLA and length<br>(+) Association between GLA and HC<br><br>1 year timepoint<br>(No) Association between GLA and weight<br>(No) Association between GLA and length<br>(+) Association between GLA and HC	Unadjusted estimates provided by authors
Miliku et al.<br>Canada, 2019<br>(HIC)	Longitudinal<br>1094 (subset of cohort)	3-4 mo	3 mo and 1 year	Mean 0.10% (SD 0.05%)	Weight<br>Length	3 month timepoint<br>(No) Association between GLA and weight<br>(No) Association between GLA and length<br><br>1 year timepoint<br>(No) Association between GLA and weight<br>(No) Association between GLA and length	Unadjusted estimates provided by authors
Mychaleckyj et al.<br>Bangladesh, 2020<br>(LMIC)	Longitudinal<br>700 enrolled<br>563 analyzed	3–43 days	6 wks, 1 year, and 2 years	Mean 0.16% (SD 0.11%)	WAZ<br>LAZ<br>WHO reference standards	Difference between 6 wks and 1 year<br>(+) Association between GLA and change in LAZ<br>(No) Association between GLA and change in WAZ<br><br>Difference between 6 wks and 2 years<br>(+) Association between LA and change in LAZ<br>(No) Association between LA and change in WAZ	Infant serum zinc<br>Infant sex<br>Infant age at the time of sample and data collection<br>Infant gestational age<br>Human milk AA and DHA, log(%AA) and log(%DHA)
Peng et al. 2021, China<br>(UMIC)	Longitudinal<br>101	1, 2 and 3 mo	1, 2 and 3 mo	Fatty Acids<br><br>(realtive abundance and ratios)	Weight, length, BMI, head circumference	(No) Associations reported.	none
**18:2c-9, t-11 (conjugated linoleic acid, CLA)**							
Miliku et al.<br>Canada, 2019<br>(HIC)	Longitudinal<br>1094 (subset of cohort)	3-4 mo	3 mo and 1 year	Mean 0.02% (SD 0.01%)	Weight<br>Length	3 month timepoint<br>(-) Association between CLA and weight<br>(-) Association between CLA and length<br><br>1 year timepoint<br>(No) Association between CLA and weight<br>(No) Association between CLA and length	Unadjusted estimates provided by authors
**20:2c-11, c-14 or 20:2n6 (eicosadienoic acid, EDA)**							
Jacobson et al.<br>Canada, 2008<br>(HIC)	Longitudinal<br>109 enrolled<br>74 analyzed at 6 mo<br>67 analyzed at 12 mo	6 mo and 1 year	6 mo and 1 year	Data not presented	Weight<br>Length<br>HC	6 mo timepoint<br>(No) Association between EDA and weight<br>(No) Association between EDA and length<br>(No) Association between EDA and HC<br><br>1 year timepoint<br>(No) Association between EDA and weight<br>(No) Association between EDA and length<br>(No) Association between EDA and HC	Unadjusted estimates provided by authors
Miliku et al.<br>Canada, 2019<br>(HIC)	Longitudinal<br>1094 (subset of cohort)	3-4 mo	3 mo and 1 year	Mean 0.20% (SD 0.05%)	Weight<br>Length	3 month timepoint<br>(-) Association between EDA and weight<br>(-) Association between EDA and length<br><br>1 year timepoint<br>(No) Association between EDA and weight<br>(No) Association between EDA and length	Unadjusted estimates provided by authors
Mychaleckyj et al.<br>Bangladesh, 2020<br>(LMIC)	Longitudinal<br>700 enrolled<br>563 analyzed	3–43 days	6 wks, 1 year, and 2 years	Mean 0.41% (SD 0.18%)	WAZ<br>LAZ<br>WHO reference standards	Difference between 6 wks and 1 year<br>(No) Association between EDA and change in LAZ<br>(No) Association between EDA and change in WAZ<br><br>Difference between 6 wks and 2 years<br>(No) Association between EDA and change in LAZ<br>(No) Association between EDA and change in WAZ	Infant serum zinc<br>Infant sex<br>Infant age at the time of sample and data collection<br>Infant gestational age<br>Human milk AA and DHA, log(%AA) and log(%DHA)
**20:3n-6 (dihomo-gamma-linolenic acid, DGLA)**							

Table S5. Characteristics and results of included studies reporting on human milk fat and fatty acids and infant anthropometrics - organized by component.

Authors, country, publication year (income setting)	Design and participants	Timing of milk sampling	Timing of infant anthropometrics	Estimated intake or HM concentration*	Anthropometric outcome measures and standards	Associations**	Major confounders considered
George et al. 2021, Australia (HIC)	Cohort 30 enrolled, 18 analysed	Birth, 1, 2, 3, 4, 5, 6 mo postpartum	Birth, 1, 2, 3, 4, 5, 6 mo postpartum	Fatty Acids<br><br>(daily intake)	Weight, length, head circumference, WFL Z Score, HC Z score, BMI	Monthly intake and growth (adjusted for multiple comparisons)<br>(No) Association between 20:3n-6 and HCZ, HC, WLZ weight, length and BMI<br><br>Intake and growth at 6 mo (adjusted for multiple comparisons)<br>(No) Association between 20:3n-6 and HCZ, HC, WLZ weight, length and BMI	None reported
Jacobson et al. Canada, 2008 (HIC)	Longitudinal 109 enrolled 74 analyzed at 6 mo 67 analyzed at 12 mo	6 mo and 1 year	6 mo and 1 year	Data not presented	Weight<br>Length<br>HC	6 mo timepoint<br>(No) Association between DGLA and weight<br>(No) Association between DGLA and length<br>(No) Association between DGLA and HC<br><br>1 year timepoint<br>(No) Association between DGLA and weight<br>(No) Association between DGLA and length<br>(No) Association between DGLA and HC	Unadjusted estimates provided by authors
Miliku et al. Canada, 2019 (HIC)	Longitudinal 1094 (subset of cohort)	3-4 mo	3 mo and 1 year	Mean 0.35% (SD 0.11%)	Weight<br>Length	3 month timepoint<br>(-) Association between DGLA and weight<br>(No) Association between DGLA and length<br><br>1 year timepoint<br>(No) Association between DGLA and weight<br>(No) Association between DGLA and length	Unadjusted estimates provided by authors
Mychaleckyj et al. Bangladesh, 2020 (LMIC)	Longitudinal 700 enrolled 563 analyzed	3–43 days	6 wks, 1 year, and 2 years	Mean 0.55% (SD 0.17%)	WAZ<br>LAZ<br>WHO reference standards	Difference between 6 wks and 1 year<br>(No) Association between DGLA and change in LAZ<br>(No) Association between DGLA and change in WAZ<br><br>Difference between 6 wks and 2 years<br>(No) Association between DGLA and change in LAZ<br>(No) Association between DGLA and change in WAZ	Infant serum zinc<br>Infant sex<br>Infant age at the time of sample and data collection<br>Infant gestational age<br>Human milk AA and DHA, log(%AA) and log(%DHA)
Peng et al. 2021, China (UMIC)	Longitudinal 101	1, 2 and 3 mo	1, 2 and 3 mo	Fatty Acids<br><br>(realtive abundance and ratios)	Weight, length, BMI, head circumference	(No) Associations reported.	none
**20:4n-6 (arachidonic acid, AA)**							
De la Garza Puentes et al. Spain, 2019 (HIC)	Longitudinal 78 (subset of cohort)	2-4 days (colostrum) and 28-32 days (mature milk)	6 and 18 mo	Mean 0.49% (SD 0.05%) in normal weight mothers, 0.49% (SD 0.12%) in overweight mothers, 0.47% (0.10%) in mothers with obesity	BMIZ<br>WAZ<br>LAZ<br>WHO reference standards	6 mo timepoint (colostrum sample)(-)<br>Association between AA and BMIZ (No)<br>Association between AA and WAZ (No)<br>Association between AA and LAZ<br><br>6 mo timepoint (mature milk sample)<br>(No) Association between AA and BMIZ<br>(No) Association between AA and WAZ<br>(No) Association between AA and LAZ<br><br>18 mo timepoint (colostrum sample)<br>(No) Association between AA and BMIZ<br>(No) Association between AA and WAZ<br>(No) Association between AA and LAZ<br><br>18 mo timepoint (mature milk sample)<br>(No) Association between AA and BMIZ<br>(No) Association between AA and WAZ<br>(No) Association between AA and LAZ	Infant sex<br>Maternal BMI<br>Maternal weight gain during pregnancy<br>Maternal smoking<br>Maternal education<br>Infant feeding practices
George et al. 2021, Australia (HIC)	Cohort 30 enrolled, 18 analysed	Birth, 1, 2, 3, 4, 5, 6 mo postpartum	Birth, 1, 2, 3, 4, 5, 6 mo postpartum	Fatty Acids<br><br>(daily intake)	Weight, length, head circumference, WFL Z Score, HC Z score, BMI	Monthly intake and growth (adjusted for multiple comparisons)<br>(No) Association between arachidonic acid and HCZ, HC, WLZ weight, length and BMI<br><br>Intake and growth at 6 mo (adjusted for multiple comparisons)<br>(No) Association between arachidonic acid and HCZ, HC, WLZ weight, length and BMI	None reported
Miliku et al. Canada, 2019 (HIC)	Longitudinal 1094 (subset of cohort)	3-4 mo	3 mo and 1 year	Mean 0.38% (SD 0.09%)	Weight<br>Length	3 month timepoint<br>(-) Association between AA and weight<br>(No) Association between AA and length<br><br>1 year timepoint<br>(No) Association between AA and weight<br>(No) Association between AA and length	Unadjusted estimates provided by authors

Table S5. Characteristics and results of included studies reporting on human milk fat and fatty acids and infant anthropometrics - organized by component.

Authors, country, publication year (income setting)	Design and participants	Timing of milk sampling	Timing of infant anthropometrics	Estimated intake or HM concentration*	Anthropometric outcome measures and standards	Associations**	Major confounders considered
Much et al.<br>Germany, 2013<br>(HIC)<br><br>Meyer et al.<br>Germany, 2019<br>(HIC)	Randomized controlled trial<br>208 infants enrolled<br>152 analyzed at 6 wks<br>120 analyzed at 4 mo	6 wks and 4 mo	6 wks, 4 mo 1 year, 2 years	6 week timepoint<br>Mean 0.43% (SD 0.08%) in control group, 0.43% (0.08%) in intervention group<br><br>4 month timepoint<br>Mean 0.40% (SD 0.08%) in control group, 0.40% (0.07%) in intervention group	Weight<br>BMI<br>Length<br>Body composition (skinfold thickness, fat mass, fat mass %, subcutaneous/preperitoneal fat, ponderal index)	6 wks timepoint (6 week sample)<br>(No) Association between AA and weight<br>(No) Association between AA and BMI<br>(No) Association between AA and length<br>(No) Association between AA and skinfold thickness<br>(-) Association between AA and fat mass<br>(No) Association between AA and fat mass %<br>(No) Association between AA and subcutaneous/preperitoneal fat<br><br>4 mo timepoint (6 week sample)<br>(No) Association between AA and weight<br>(No) Association between AA and BMI<br>(No) Association between AA and length<br>(No) Association between AA and skinfold thickness<br>(No) Association between AA and fat mass or fat mass %<br>(No) Association between AA and subcutaneous/preperitoneal fat<br><br>4 mo timepoint (4 month sample)<br>(No) Association between AA and weight<br>(No) Association between AA and BMI<br>(No) Association between AA and length<br>(No) Association between AA and skinfold thickness<br>(No) Association between AA and fat mass<br>(No) Association between AA and fat mass %<br>(No) Association between AA and subcutaneous/preperitoneal fat<br><br>1 year timepoint (6 week sample)<br>(No) Association between AA and weight<br>(No) Association between AA and BMI<br>(No) Association between AA and length<br>(No) Association between AA and skinfold thickness<br>(No) Association between AA and fat mass or fat mass %<br>(No) Association between AA and subcutaneous/preperitoneal fat<br><br>1 year timepoint (4 month sample)<br>(No) Association between AA and weight<br>(No) Association between AA and BMI<br>(No) Association between AA and length<br>(No) Association between AA and skinfold thickness<br>(No) Association between AA and fat mass or fat mass %<br>(No) Association between AA and subcutaneous/preperitoneal fat<br><br>2 year timepoint (6 week sample)<br>(No) Association between AA and weight<br>(No) Association between AA and BMI<br>(No) Association between AA and length<br>(No) Association between AA and skinfold thickness<br>(No) Association between AA and fat mass or fat mass %<br>(No) Association between AA and subcutaneous/preperitoneal fat<br><br>2 year timepoint (4 month sample)<br>(No) Association between AA and weight<br>(No) Association between AA and BMI<br>(No) Association between AA and length<br>(No) Association between AA and skinfold thickness<br>(No) Association between AA and fat mass or fat mass %<br>(No) Association between AA and subcutaneous/preperitoneal fat	Infant sex<br>Infant gestational age<br>Infant ponderal index at birth<br>Maternal parity<br>Study group<br>Breastfeeding
Mychaleckyj et al.<br>Bangladesh, 2020<br>(LMIC)	Longitudinal<br>700 enrolled<br>683 with human milk samples<br>563 with primary outcome anthropometry	3–43 days (mean 10.4, SD 6.3)	6, 52, and 104 wks	Mean 0.53% (SD 0.15%)	WAZ<br>LAZ<br>WHO reference standards	Difference between 6 wks and 1 year<br>(No) Association between AA and change in LAZ<br>(No) Association between AA and change in WAZ<br><br>Difference between 6 wks and 2 years<br>(No) Association between AA and change in LAZ<br>(No) Association between AA and change in WAZ	Infant serum zinc<br>Infant sex<br>Infant age at the time of sample and data collection<br>Infant gestational age<br>Human milk AA and DHA, log(%AA) and log(%DHA)
Peng et al. 2021, China (UMIC)	Longitudinal<br>101	1, 2 and 3 mo	1, 2 and 3 mo	Fatty Acids<br><br>(concentration)	Weight, length, BMI, head circumference	(No) Associations reported.	none
Scholtens et al.<br>The Netherlands, 2009<br>(HIC)	Longitudinal<br>244 enrolled (subset of cohort)<br>177 analyzed	3-4 mo	1 year	Mean 0.370% (SD 0.092%)	Weight<br>Length<br>BMI	Difference between birth and 1 year<br>(No) Association between AA and weight gain<br>(-) Association between AA and BMI gain<br>(No) Association between AA and length gain	Infant age<br>Breastfeeding duration<br>Low and high fatty acid tertiles<br>Sample collection time

Table S5. Characteristics and results of included studies reporting on human milk fat and fatty acids and infant anthropometrics - organized by component.

Authors, country, publication year (income setting)	Design and participants	Timing of milk sampling	Timing of infant anthropometrics	Estimated intake or HM concentration*	Anthropometric outcome measures and standards	Associations**	Major confounders considered
Xiang et al.<br>China, 1999<br>(LMIC)	Cross-sectional<br>41 (18 infants 1 month old, 23 infants 3 mo old)	1 or 3 mo	1 or 3 mo	Mean 0.63% (SEM 0.03%) at 1 month, 0.51% (SEM 0.02%) at 3 mo	Weight<br>Length	Difference between birth and 1 month<br>(No) Association between AA and weight gain<br>(No) Association between AA and length gain<br><br>Difference between birth and 3 mo<br>(+) Association between AA and weight gain<br>(No) Association between AA and length gain	None
**22:4n-6 (docosatetraenoic acid, DTA)**							
Jacobson et al.<br>Canada, 2008<br>(HIC)	Longitudinal<br>109 enrolled<br>74 analyzed at 6 mo<br>67 analyzed at 12 mo	6 mo and 1 year	6 mo and 1 year	Data not presented	Weight<br>Length<br>HC	6 mo timepoint<br>(No) Association between DTA and weight<br>(No) Association between DTA and length<br>(No) Association between DTA and HC<br><br>1 year timepoint<br>(No) Association between DTA and weight<br>(No) Association between DTA and length<br>(No) Association between DTA and HC	Unadjusted estimates provided by authors
Miliku et al.<br>Canada, 2019<br>(HIC)	Longitudinal<br>1094 (subset of cohort)	3-4 mo	3 mo and 1 year	Mean 0.04% (SD 0.03%)	Weight<br>Length	3 month timepoint<br>(No) Association between DTA and weight<br>(No) Association between DTA and length<br><br>1 year timepoint<br>(No) Association between DTA and weight<br>(No) Association between DTA and length	Unadjusted estimates provided by authors
Mychaleckyj et al.<br>Bangladesh, 2020<br>(LMIC)	Longitudinal<br>700 enrolled<br>683 with human milk samples<br>563 with primary outcome anthropometry	3–43 days (mean 10.4, SD 6.3)	6, 52, and 104 wks	Mean 0.18% (SD 0.10%)	WAZ<br>LAZ<br>WHO reference standards	Difference between 6 wks and 1 year<br>(No) Association between DTA and change in LAZ<br>(No) Association between DTA and change in WAZ<br><br>Difference between 6 wks and 2 years<br>(No) Association between DTA and change in LAZ<br>(No) Association between DTA and change in WAZ	Infant serum zinc<br>Infant sex<br>Infant age at the time of sample and data collection<br>Infant gestational age<br>Human milk AA and DHA, log(%AA) and log(%DHA)
**22:5n-6 (docosapentaenoic-n6 acid, DPA6)**							
Jacobson et al.<br>Canada, 2008<br>(HIC)	Longitudinal<br>109 enrolled<br>74 analyzed at 6 mo<br>67 analyzed at 12 mo	6 mo and 1 year	6 mo and 1 year	Data not presented	Weight<br>Length<br>HC	6 mo timepoint<br>(No) Association between DPA6 and weight<br>(No) Association between DPA6 and length<br>(No) Association between DPA6 and HC<br><br>1 year timepoint<br>(No) Association between DPA6 and weight<br>(No) Association between DPA6 and length<br>(No) Association between DPA6 and HC	Unadjusted estimates provided by authors
Miliku et al.<br>Canada, 2019<br>(HIC)	Longitudinal<br>1094 (subset of cohort)	3-4 mo	3 mo and 1 year	Mean 0.03% (SD 0.01%)	Weight<br>Length	3 month timepoint<br>(No) Association between DPA6 and weight<br>(No) Association between DPA6 and length<br><br>1 year timepoint<br>(No) Association between DPA6 and weight<br>(-) Association between DPA6 and length	Unadjusted estimates provided by authors
Mychaleckyj et al.<br>Bangladesh, 2020<br>(LMIC)	Longitudinal<br>700 enrolled<br>683 with human milk samples<br>563 with primary outcome anthropometry	3–43 days (mean 10.4, SD 6.3)	6, 52, and 104 wks	Mean 0.39% (SD 0.14%)	WAZ<br>LAZ<br>WHO reference standards	Difference between 6 wks and 1 year<br>(No) Association between DPA6 and change in LAZ<br>(No) Association between DPA6 and change in WAZ<br><br>Difference between 6 wks and 2 years<br>(No) Association between DPA6 and change in LAZ<br>(No) Association between DPA6 and change in WAZ	Infant serum zinc<br>Infant sex<br>Infant age at the time of sample and data collection<br>Infant gestational age<br>Human milk AA and DHA, log(%AA) and log(%DHA)
**n-6 PUFAs**							

Table S5. Characteristics and results of included studies reporting on human milk fat and fatty acids and infant anthropometrics - organized by component.

Authors, country, publication year (income setting)	Design and participants	Timing of milk sampling	Timing of infant anthropometrics	Estimated intake or HM concentration*	Anthropometric outcome measures and standards	Associations**	Major confounders considered
De la Garza Puentes et al.<br>Spain, 2019<br>(HIC)	Longitudinal<br>78 (subset of cohort)	2-4 days (colostrum) and 28-32 days (mature milk)	6 and 18 mo	Mean 15.24% (SD 3.31%) in normal weight mothers, 16.89% (SD 3.93%) in overweight mothers, 15.64% (3.19%) in mothers with obesity	BMIZ<br>WAZ<br>LAZ<br>WHO reference standards	6 mo timepoint (colostrum sample)<br>(No) Association between total n-6 PUFAs and BMIZ<br>(No) Association between total n-6 PUFAs and WAZ<br>(No) Association between total n-6 PUFAs and LAZ<br><br>6 mo timepoint (mature milk sample)<br>(No) Association between total n-6 PUFAs and BMIZ<br>(No) Association between total n-6 PUFAs and WAZ<br>(No) Association between total n-6 PUFAs and LAZ<br><br>18 mo timepoint (colostrum sample)<br>(No) Association between total n-6 PUFAs and BMIZ<br>(No) Association between total n-6 PUFAs and WAZ<br>(No) Association between total n-6 PUFAs and LAZ<br><br>18 mo timepoint (mature milk sample)<br>(No) Association between total n-6 PUFAs and BMIZ<br>(No) Association between total n-6 PUFAs and WAZ<br>(No) Association between total n-6 PUFAs and LAZ	Infant sex<br>Maternal BMI<br>Maternal weight gain during pregnancy<br>Maternal smoking<br>Maternal education<br>Infant feeding practices
Makela et al.<br>Finland, 2013<br>(HIC)	Longitudinal<br>100 enrolled<br>88 with anthropometry	3 mo	13 mo	Mean 11.4% (SD 2.1%) in overweight mothers, 12.1% (SD 3.4%) in normal weight mothers	Weight<br>BMI<br>Length	13 mo timepoint<br>(No) Association between total n-6 PUFAs and weight<br>(No) Association between total n-6 PUFAs and BMI<br>(No) Association between total n-6 PUFAs and length<br><br>Difference between birth and 13 mo<br>(No) Association between total n-6 PUFAs and weight gain<br>(No) Association between total n-6 PUFAs and BMI gain	None
Nuss et al.<br>USA, 2019<br>(HIC)	Cross-sectional<br>33	4-8 wks	4-8 wks	Mean 18.8% (SD 4.0%)	Weight<br>Length<br>HC<br>Body composition (fat mass %)	(-) Association between total n-6 PUFAs and weight<br>(No) Association between total n-6 PUFAs and length<br>(-) Association between total n-6 PUFAs and HC<br>(-) Association between total n-6 PUFAs and fat mass %	Infant age
Peng et al. 2021, China (UMIC)	Longitudinal<br>101	1, 2 and 3 mo	1, 2 and 3 mo	Fatty Acids<br><br>(realtive abundance and ratios)	Weight, length, BMI, head circumference	(No) Associations reported.	none
Scholtens et al.<br>The Netherlands, 2009<br>(HIC)	Longitudinal<br>244 enrolled<br>(subset of cohort)<br>177 analyzed	3-4 mo	1 year	Mean 16.197% (SD 4.197%)	Weight<br>BMI<br>Length	Difference between birth and 1 year<br>(-) Association between total n-6 PUFAs and weight gain<br>(-) Association between total n-6 PUFAs and BMI gain (high tertile only)<br>(No) Association between total n-6 PUFAs and length gain	Infant age<br>Breastfeeding duration<br>Low and high fatty acid tertiles<br>Sample collection time
**Total n-6 LCPUFAs**							
De la Garza Puentes et al.<br>Spain, 2019<br>(HIC)	Longitudinal<br>78 (subset of cohort)	2-4 days (colostrum) and 28-32 days (mature milk)	6 and 18 mo	Mean 1.48% (SD 0.16%) in normal weight mothers, 1.52% (SD 0.24%) in overweight mothers, 1.58% (0.34%) in mothers with obesity	BMIZ<br>WAZ<br>LAZ<br>WHO reference standards	6 mo timepoint (mature milk sample)<br>(-) Association between total n-6 LCPUFAs and BMIZ<br>(No) Association between total n-6 LCPUFAs and WAZ<br>(No) Association between total n-6 LCPUFAs and LAZ<br><br>6 mo timepoint (mature milk sample)<br>(No) Association between total n-6 LCPUFAs and BMIZ<br>(No) Association between total n-6 LCPUFAs and WAZ<br>(No) Association between total n-6 LCPUFAs and LAZ<br><br>18 mo timepoint (colostrum sample)<br>(No) Association between total n-6 LCPUFAs and BMIZ<br>(No) Association between total n-6 LCPUFAs and WAZ<br>(No) Association between total n-6 LCPUFAs and LAZ<br><br>18 mo timepoint (mature milk sample)<br>(No) Association between total n-6 LCPUFAs and BMIZ<br>(No) Association between total n-6 LCPUFAs and WAZ<br>(No) Association between total n-6 LCPUFAs and LAZ	Infant sex<br>Maternal BMI<br>Maternal weight gain during pregnancy<br>Maternal smoking<br>Maternal education<br>Infant feeding practices

Table S5. Characteristics and results of included studies reporting on human milk fat and fatty acids and infant anthropometrics - organized by component.

Authors, country, publication year (income setting)	Design and participants	Timing of milk sampling	Timing of infant anthropometrics	Estimated intake or HM concentration*	Anthropometric outcome measures and standards	Associations**	Major confounders considered
Much et al.<br>Germany, 2013<br>(HIC)<br><br>Meyer et al.<br>Germany, 2019<br>(HIC)	Randomized controlled trial<br>208 infants enrolled<br>152 analyzed at 6 wks<br>120 analyzed at 4 mo	6 wks and 4 mo	6 wks, 4 mo 1 year, 2 years	6 week timepoint<br>Mean 1.23% (SD 0.21%) in control group, 1.22% (0.20%) in intervention group<br><br>4 month timepoint<br>Mean 1.07% (SD 0.21%) in control group, 1.07% (0.18%) in intervention group	Weight<br>BMI<br>Length<br>Body composition (skinfold thickness, fat mass, fat mass %, subcutaneous/preperitoneal fat, ponderal index)	6 wks timepoint (6 week sample)<br>(No) Association between total n-6 LCPUFAs and weight<br>(No) Association between total n-6 LCPUFAs and BMI<br>(No) Association between total n-6 LCPUFAs and length<br>(-) Association between total n-6 LCPUFAs and skinfold thickness<br>(-) Association between total n-6 LCPUFAs and fat mass and fat mass %<br>(No) Association between total n-6 LCPUFAs and subcutaneous/preperitoneal fat<br><br>4 mo timepoint (6 week sample)<br>(No) Association between total n-6 LCPUFAs and weight<br>(No) Association between total n-6 LCPUFAs and BMI<br>(No) Association between total n-6 LCPUFAs and length<br>(No) Association between total n-6 LCPUFAs and skinfold thickness<br>(No) Association between total n-6 LCPUFAs and fat mass or fat mass %<br>(No) Association between total n-6 LCPUFAs and subcutaneous/preperitoneal fat<br><br>4 mo timepoint (4 month sample)<br>(No) Association between total n-6 LCPUFAs and weight<br>(No) Association between total n-6 LCPUFAs and BMI<br>(No) Association between total n-6 LCPUFAs and length<br>(No) Association between total n-6 LCPUFAs and skinfold thickness<br>(No) Association between total n-6 LCPUFAs and fat mass or fat mass %<br>(No) Association between total n-6 LCPUFAs and subcutaneous/preperitoneal fat<br><br>1 year timepoint (6 week sample)<br>(No) Association between total n-6 LCPUFAs and weight<br>(No) Association between total n-6 LCPUFAs and BMI<br>(No) Association between total n-6 LCPUFAs and length<br>(No) Association between total n-6 LCPUFAs and skinfold thickness<br>(No) Association between total n-6 LCPUFAs and fat mass or fat mass %<br>(No) Association between total n-6 LCPUFAs and subcutaneous/preperitoneal fat<br><br>1 year timepoint (4 month sample)<br>(No) Association between total n-6 LCPUFAs and weight<br>(No) Association between total n-6 LCPUFAs and BMI<br>(No) Association between total n-6 LCPUFAs and length<br>(No) Association between total n-6 LCPUFAs and skinfold thickness<br>(No) Association between total n-6 LCPUFAs and fat mass or fat mass %<br>(No) Association between total n-6 LCPUFAs and subcutaneous/preperitoneal fat<br><br>2 year timepoint (6 week sample)<br>(No) Association between total n-6 LCPUFAs and weight<br>(No) Association between total n-6 LCPUFAs and BMI<br>(No) Association between total n-6 LCPUFAs and length<br>(No) Association between total n-6 LCPUFAs and skinfold thickness<br>(No) Association between total n-6 LCPUFAs and fat mass or fat mass %<br>(No) Association between total n-6 LCPUFAs and subcutaneous/preperitoneal fat<br><br>2 year timepoint (4 month sample)<br>(No) Association between total n-6 LCPUFAs and weight<br>(No) Association between total n-6 LCPUFAs and BMI<br>(No) Association between total n-6 LCPUFAs and length<br>(No) Association between total n-6 LCPUFAs and skinfold thickness<br>(No) Association between total n-6 LCPUFAs and fat mass or fat mass %<br>(No) Association between total n-6 LCPUFAs and subcutaneous/preperitoneal fat	Infant sex<br>Infant gestational age<br>Infant ponderal index at birth<br>Maternal parity<br>Study group<br>Breastfeeding
Scholtens et al.<br>The Netherlands, 2009<br>(HIC)	Longitudinal<br>244 enrolled<br>(subset of cohort)<br>177 analyzed	3-4 mo	1 year	Mean 1.126% (SD 0.276%)	Weight<br>BMI<br>Length	Difference between birth and 1 year<br>(No) Association between total n-6 LCPUFAs and weight gain<br>(No) Association between total n-6 LCPUFAs and BMI gain<br>(No) Association between total n-6 LCPUFAs and length gain	Infant age<br>Breastfeeding duration<br>Low and high fatty acid tertiles<br>Sample collection time
**Fatty acid ratios**							
**n-6:n-3 PUFAs**							

Table S5. Characteristics and results of included studies reporting on human milk fat and fatty acids and infant anthropometrics - organized by component.

Authors, country, publication year (income setting)	Design and participants	Timing of milk sampling	Timing of infant anthropometrics	Estimated intake or HM concentration*	Anthropometric outcome measures and standards	Associations**	Major confounders considered
De la Garza Puentes et al.<br>Spain, 2019<br>(HIC)	Longitudinal<br>78 (subset of cohort)	2-4 days (colostrum) and 28-32 days (mature milk)	6 and 18 mo	Mean 15.08% (SD 3.91) in normal weight mothers, 18.28% (SD 5.33) in overweight mothers, 17.80% (4.97) in mothers with obesity	BMIZ<br>WAZ<br>LAZ<br>WHO reference standards	6 mo timepoint (colostrum sample)<br>(+) Association between n-6:n-3 PUFAs and BMIZ<br>(+) Association between n-6:n-3 PUFAs and WAZ<br>(No) Association between n-6:n-3 PUFAs and LAZ<br><br>6 mo timepoint (mature milk sample)<br>(No) Association between n-6:n-3 PUFAs and BMIZ<br>(+) Association between n-6:n-3 PUFAs and WAZ<br>(No) Association between n-6:n-3 PUFAs and LAZ<br><br>18 mo timepoint (colostrum sample)<br>(No) Association between n-6:n-3 PUFAs and BMIZ<br>(No) Association between n-6:n-3 PUFAs and WAZ<br>(No) Association between n-6:n-3 PUFAs and LAZ<br><br>18 mo timepoint (mature milk sample)<br>(No) Association between n-6:n-3 PUFAs and BMIZ<br>(No) Association between n-6:n-3 PUFAs and WAZ<br>(No) Association between n-6:n-3 PUFAs and LAZ	Infant sex<br>Maternal BMI<br>Maternal weight gain during pregnancy<br>Maternal smoking<br>Maternal education<br>Infant feeding practices
Enstad et al.<br>USA, 2020<br>(HIC)	Longitudinal<br>40	1 and 4 mo	Monthly between 1 and 7 mo	Data not presented	Weight z-scores<br>BMIZ<br>Length z-scores<br>Body composition (fat mass % and lean mass %)	4 mo timepoint<br>(No) Association between n-6:n-3 PUFAs and weight z-scores<br>(No) Association between n-6:n-3 PUFAs and BMIZ<br>(+) Association between n-6:n-3 PUFAs and length z-scores<br>(No) Association between n-6:n-3 PUFAs and fat mass %<br>(No) Association between n-6:n-3 PUFAs and lean mass %<br><br>7 mo timepoint<br>(No) Association between n-6:n-3 PUFAs and weight z-scores<br>(+) Association between n-6:n-3 PUFAs and BMIZ<br>(No) Association between n-6:n-3 PUFAs and length z-scores<br>(No) Association between n-6:n-3 PUFAs and fat mass %<br>(No) Association between n-6:n-3 PUFAs and lean mass %<br><br>Difference between 1 and 7 mo<br>(+) Association between n-6:n-3 PUFAs and weight z-score<br>(+) Association between n-6:n-3 PUFAs and change in BMIZ<br>(+) Association between n-6:n-3 PUFAs and change in length z-score<br>(No) Association between n-6:n-3 PUFAs and fat mass % change<br>(No) Association between n-6:n-3 PUFAs and lean mass % change	Infant sex<br>Infant age<br>Maternal BMI<br>Maternal ethnicity
Makela et al.<br>Finland, 2013<br>(HIC)	Longitudinal<br>100 enrolled<br>88 with anthropometry	3 mo	13 mo	Mean 5.7% (SD 1.8) in overweight mothers, 4.9% (SD 1.6) in normal weight mothers	Weight<br>BMI<br>Length	13 mo timepoint<br>(No) Association between n-6:n-3 PUFAs and weight<br>(No) Association between n-6:n-3 PUFAs and length<br>(No) Association between n-6:n-3 PUFAs and BMI<br><br>Difference between birth and 13 mo<br>(No) Association between n-6:n-3 PUFAs and weight gain<br>(No) Association between n-6:n-3 PUFAs and BMI gain	None
Nuss et al.<br>USA, 2019<br>(HIC)	Cross-sectional<br>33	4-8 wks	4-8 wks	Mean 14.9% (SD 4.57) in overweight mothers, 14.0% (SD 3.35) in normal weight mothers	Weight<br>Length<br>HC<br>Body composition (fat mass %)	(-) Association between n-6:n-3 PUFAs and weight<br>(No) Association between n-6:n-3 PUFAs and length<br>(-) Association between n-6:n-3 PUFAs and HC<br>(-) Association between n-6:n-3 PUFAs and fat mass %	Infant age
Peng et al. 2021, China (UMIC)	Longitudinal<br>101	1, 2 and 3 mo	1, 2 and 3 mo	Fatty Acids<br><br>(realtive abundance and ratios)	Weight, length, BMI, head circumference	(No) Associations reported.	none
**n-3:n-6 PUFAs**							
Scholtens et al.<br>The Netherlands, 2009<br>(HIC)	Longitudinal<br>244 enrolled<br>(subset of cohort)<br>177 analyzed	3-4 mo	1 year	Mean 0.099% (SD 0.031)	Weight<br>BMI<br>Length	Difference between birth and 1 year<br>(No) Association between n-3:n-6 PUFAs and weight gain<br>(No) Association between n-3:n-6 PUFAs and BMI gain<br>(No) Association between n-3:n-6 PUFAs and length gain	Infant age<br>Breastfeeding duration<br>Low and high fatty acid tertiles<br>Sample collection time
**n-6:n-3 LCPUFAs**							

Table S5. Characteristics and results of included studies reporting on human milk fat and fatty acids and infant anthropometrics - organized by component.

Authors, country, publication year (income setting)	Design and participants	Timing of milk sampling	Timing of infant anthropometrics	Estimated intake or HM concentration*	Anthropometric outcome measures and standards	Associations**	Major confounders considered
De la Garza Puentes et al.<br>Spain, 2019<br>(HIC)	Longitudinal<br>78 (subset of cohort)	2-4 days (colostrum) and 28-32 days (mature milk)	6 and 18 mo	Mean 3.62 (SD 1.18) in normal weight mothers, 4.28 (SD 1.24) in overweight mothers, 3.73 (0.92) in mothers with obesity	BMIZ<br>WAZ<br>LAZ<br>WHO reference standards	6 mo timepoint (colostrum sample)<br>(No) Association between n-6:n-3 LCPUFAs and BMIZ<br>(No) Association between n-6:n-3 LCPUFAs and WAZ<br>(No) Association between n-6:n-3 LCPUFAs and LAZ<br><br>6 mo timepoint (mature milk sample)<br>(No) Association between n-6:n-3 LCPUFAs and BMIZ<br>(No) Association between n-6:n-3 LCPUFAs and WAZ<br>(No) Association between n-6:n-3 LCPUFAs and LAZ<br><br>18 mo timepoint (colostrum sample)<br>(No) Association between n-6:n-3 LCPUFAs and BMIZ<br>(No) Association between n-6:n-3 LCPUFAs and WAZ<br>(No) Association between n-6:n-3 LCPUFAs and LAZ<br><br>18 mo timepoint (mature milk sample)<br>(No) Association between n-6:n-3 LCPUFAs and BMIZ<br>(No) Association between n-6:n-3 LCPUFAs and WAZ<br>(No) Association between n-6:n-3 LCPUFAs and LAZ	Infant sex<br>Maternal BMI<br>Maternal weight gain during pregnancy<br>Maternal smoking<br>Maternal education<br>Infant feeding practices
Ellsworth et al.<br>USA, 2020<br>(HIC)	Longitudinal<br>55 enrolled<br>32 analyzed	2 wks	2 wks and 2 mo	Mean 12.82 (SD 3.04) in normal weight mothers, 12.98 (SD 3.08 g/dL) in mothers with overweight or obesity	WLZ<br>BMIZ<br>WAZ<br>LAZ<br>HC<br>WHO reference standards	Difference from 2 wks to 2 mo<br>(+) Association between n-6:n-3 LCPUFAs and WLZ increase<br>(+) Association between n-6:n-3 LCPUFAs and BMIZ increase<br>(+) Association between n-6:n-3 LCPUFAs and WAZ increase<br>(No) Association between n-6:n-3 LCPUFAs and LAZ change<br>(No) Association between n-6:n-3 LCPUFAs and HC	Infant sex
Much et al.<br>Germany, 2013<br>(HIC)<br><br>Meyer et al.<br>Germany, 2019<br>(HIC)	Randomized controlled trial<br>208 infants enrolled<br>152 analyzed at 6 wks<br>120 analyzed at 4 mo	6 wks and 4 mo	6 wks, 4 mo 1 year, 2 years	6 week timepoint<br>Mean 2.02 (SD 0.59) in control group, 0.71 (0.25) in intervention group<br><br>4 month timepoint<br>Mean 1.94 (SD 0.51) in control group, 0.73 (0.25) in intervention group	Weight<br>BMI<br>Length<br>Body composition (skinfold thickness, fat mass, fat mass %, subcutaneous/preperitoneal fat, ponderal index)	6 wks timepoint (6 week sample)<br>(-) Association between n-6:n-3 LCPUFAs and BMI<br>(No) Association between n-6:n-3 LCPUFAs and skinfold thickness<br>(-) Association between n-6:n-3 LCPUFAs and ponderal index<br><br>4 mo timepoint (6 week sample)<br>(No) Association between n-6:n-3 LCPUFAs and BMI<br>(No) Association between n-6:n-3 LCPUFAs and skinfold thickness<br>(No) Association between n-6:n-3 LCPUFAs and ponderal index<br><br>4 mo timepoint (4 month sample)<br>(No) Association between n-6:n-3 LCPUFAs and BMI<br>(No) Association between n-6:n-3 LCPUFAs and skinfold thickness<br>(No) Association between n-6:n-3 LCPUFAs and ponderal index<br><br>1 year timepoint (6 week sample)<br>(No) Association between n-6:n-3 LCPUFAs and BMI<br>(No) Association between n-6:n-3 LCPUFAs and skinfold thickness<br>(No) Association between n-6:n-3 LCPUFAs and ponderal index<br><br>1 year timepoint (4 month sample)<br>(No) Association between n-6:n-3 LCPUFAs and BMI<br>(No) Association between n-6:n-3 LCPUFAs and skinfold thickness<br>(No) Association between n-6:n-3 LCPUFAs and ponderal index<br><br>2 year timepoint (6 week sample)<br>(-) Association between n-6:n-3 LCPUFAs and weight<br>(-) Association between n-6:n-3 LCPUFAs and BMI<br>(No) Association between n-6:n-3 LCPUFAs and fat mass %<br><br>2 year timepoint (4 month sample)<br>(No) Association between n-6:n-3 LCPUFAs and weight<br>(No) Association between n-6:n-3 LCPUFAs and BMI<br>(No) Association between n-6:n-3 LCPUFAs and fat mass %	Infant sex<br>Infant gestational age<br>Infant ponderal index at birth<br>Maternal parity<br>Study group<br>Breastfeeding
**n-3:n-6 LCPUFAs**							
Scholtens et al.<br>The Netherlands, 2009<br>(HIC)	Longitudinal<br>244 enrolled (subset of cohort)<br>177 analyzed	3-4 mo	1 year	Mean 0.471 (SD 0.197)	Weight<br>BMI<br>Length	Difference between birth and 1 year<br>(No) Association between n-3:n-6 LCPUFAs and weight gain<br>(No) Association between n-3:n-6 LCPUFAs and BMI gain<br>(No) Association between n-3:n-6 LCPUFAs and length gain	Infant age<br>Breastfeeding duration<br>Low and high fatty acid tertiles<br>Sample collection time
**AA:DHA**							

Table S5. Characteristics and results of included studies reporting on human milk fat and fatty acids and infant anthropometrics - organized by component.

Authors, country, publication year (income setting)	Design and participants	Timing of milk sampling	Timing of infant anthropometrics	Estimated intake or HM concentration*	Anthropometric outcome measures and standards	Associations**	Major confounders considered
Much et al.<br>Germany, 2013<br>(HIC)<br><br>Meyer et al.<br>Germany, 2019<br>(HIC)	Randomized controlled trial<br>208 infants enrolled<br>152 analyzed at 6 wks<br>120 analyzed at 4 mo	6 wks and 4 mo	6 wks, 4 mo 1 year, 2 years	6 week timepoint<br>Mean 1.77 (SD 0.60) in control group, 0.37 (SD 0.15) in intervention group<br><br>4 month timepoint<br>Mean 1.94 (SD 0.70) in control group, 0.41 (SD 0.22) in intervention group	Weight<br>BMI<br>Length<br>Body composition (skinfold thickness, fat mass, fat mass %, subcutaneous/preperitoneal fat, ponderal index)	6 wks timepoint (6 week sample)<br>(-) Association between AA:DHA and BMI<br>(No) Association between AA:DHA and skinfold thickness<br>(No) Association between AA:DHA and ponderal index<br><br>4 mo timepoint (6 week sample)<br>(No) Association between AA:DHA and BMI<br>(No) Association between AA:DHA and skinfold thickness<br>(No) Association between AA:DHA and ponderal index<br><br>4 mo timepoint (4 month sample)<br>(No) Association between AA:DHA and BMI<br>(No) Association between AA:DHA and skinfold thickness<br>(No) Association between AA:DHA and ponderal index<br><br>1 year timepoint (6 week sample)<br>(No) Association between AA:DHA and BMI<br>(No) Association between AA:DHA and skinfold thickness<br>(No) Association between AA:DHA and ponderal index<br><br>1 year timepoint (4 month sample)<br>(No) Association between AA:DHA and BMI<br>(No) Association between AA:DHA and skinfold thickness<br>(No) Association between AA:DHA and ponderal index	Infant sex<br>Infant gestational age<br>Infant ponderal index at birth<br>Maternal parity<br>Study group<br>Breastfeeding
Xiang et al.<br>Sweden, 2000<br>(LMIC)	Longitudinal<br>19	1 and 3 mo	1 and 3 mo	1 month timepoint<br>Mean 1.60 (SE 0.10)<br><br>3 month timepoint<br>Mean 1.56 (SE 0.09)	Occipito-frontal HC<br>Brain weight	Difference between birth and 1 month<br>(+) Association between AA:DHA and occipito-frontal HC gain<br>(+) Association between AA:DHA and brain weight gain<br><br>Difference between birth and 3 mo<br>(+) Association between AA:DHA and occipito-frontal HC gain<br>(+) Association between AA:DHA and brain weight gain	None reported

*Values reported as mean ± SD or median (IQR). **"No (assumed) associations = unreported associations assumed to be no association.

Abbreviations: BF, breastfeeding; HIC, high income countries; mo, months; HM, human milk; LMIC, low and middle income countries; NCHS, National Center for Health Statistics; RCT, randomized controlled trial; SCM, subclinical mastitis; WHO, World Health Organization; wks, weeks

Anthropometrics: BMI, body mass indez; HAZ, height for age z-score; HC, head circumference; HCAZ, head circumference z-score; LAZ, length for age Z-score; LFA, length for age; WAZ, weight for age z-score; WFA, weight for age; WLZ, weight-for-length z-score

Components: ARA, arachidonic acid; DHA, docosahexaenoic acid; EPA, eicosapentaenoic acid; LCPUFA, long chain polyunsaturated fatty acids; PUFA, polyunsaturated fatty acids

References

Abdelhamid ER, Kamhawy AH, Elkhatib AA, Megawer AS, El Shafie AI, El Gendy YG, et al. Breast Milk Macronutrients in Relation to Infants' Anthropometric Measures. Vol. 8, *Open Access Macedonian Journal of Medical Sciences.* 2020. p. 845–50.

Aksit S, Ozkayin N, Caglayan S. Effect of sucking characteristics on breast milk creamatocrit. *Paediatr Perinat Epidemiol.* 2002;16(4):355–60.

Babiszewska M. Effects of energy and essential fatty acids content in breast milk on infant's head dimensions. *Am J Hum Biol.* 2020 Nov;32(6):e23418.

Baldeon ME, Zertuche F, Flores N, Fornasini M. Free Amino Acid Content in Human Milk is Associated with Infant Gender and Weight Gain during the First Four Months of Lactation. *Nutrients.* 2019;11(9):17.

Brown KH, Akhtar NA, Robertson AD, Ahmed MG. Lactational capacity of marginally nourished mothers: relationships between maternal nutritional status and quantity and proximate composition of milk. *Pediatrics.* 1986;78(5):909–19.

Cheema AS, Stinson LF, Rea A, Lai CT, Payne MS, Murray K, et al. Human Milk Lactose, Insulin, and Glucose Relative to Infant Body Composition during Exclusive Breastfeeding. *Nutrients.* 2021 Oct 22;13(11).

Cisse AS, Dossou N, Ndiaye M, Gueye AL, Diop el HI, Diaham B, et al. Stable isotope aided evaluation of community nutrition program: effect of food supplementation schemes on maternal and infant nutritional status. *Food Nutr Bull.* 2002;23(3 Suppl):169–73.

de Fluiter KS, Kerkhof GF, van Beijsterveldt IALP, Breij LM, van de Heijning BJM, Abrahamse-Berkeveld M, et al. Longitudinal human milk macronutrients, body composition and infant appetite during early life. *Clin Nutr.* 2021 May;40(5):3401–8.

de la Garza Puentes A, Marti Alemany A, Chisaguano AM, Montes Goyanes R, Castellote AI, Torres-Espinola FJ, et al. The Effect of Maternal Obesity on Breast Milk Fatty Acids and Its Association with Infant Growth and Cognition-The PREOBE Follow-Up. *Nutrients.* 2019;11(9):09.

De Luca A, Frasquet-Darrieux M, Gaud MA, Christin P, Boquien CY, Millet C, et al. Higher Leptin but Not Human Milk Macronutrient Concentration Distinguishes Normal-Weight from Obese Mothers at 1-Month Postpartum. *PLoS One.* 2016 Dec 22;11(12):e0168568.

Dewey KG, Heinig MJ, Nommsen LA, Peerson JM, Lönnerdal B. Breast-fed infants are leaner than formula-fed infants at 1 y of age: the DARLING study. *Am J Clin Nutr.* 1993 Feb;57(2):140–5.

Ding Y, Yang Y, Xu F, Ye M, Hu P, Jiang W, et al. Association between dietary fatty acid patterns based on principal component analysis and fatty acid compositions of serum and breast milk in lactating mothers in Nanjing, China. *Food Funct.* 2021 Sep 20;12(18):8704–14.

Dorea JG. Is zinc a first limiting nutrient in human milk? *Nutr Res.* 1993;13(6):659–66.

Ellsworth L, Perng W, Harman E, Das A, Pennathur S, Gregg B. Impact of maternal overweight and obesity on milk composition and infant growth. *Matern Child Nutr.* 2020 Jul;16(3):e12979.

Enstad S, Cheema S, Thomas R, Fichorova RN, Martin CR, O'Tierney-Ginn P, et al. The impact of maternal obesity and breast milk inflammation on developmental programming of infant growth. *Eur J Clin Nutr.* 2020;

Fields DA, Demerath EW. Relationship of insulin, glucose, leptin, IL-6 and TNF-alpha in human breast milk with infant growth and body composition. *Pediatr Obes.* 2012;7(4):304–12.

George AD, Gay MCL, Selvalatchmanan J, Torta F, Bendt AK, Wenk MR, et al. Healthy Breastfeeding Infants Consume Different Quantities of Milk Fat Globule Membrane Lipids. *Nutrients.* 2021 Aug 25;13(9).

George AD, Gay MCL, Wlodek ME, Murray K, Geddes DT. The Fatty Acid Species and Quantity Consumed by the Breastfed Infant Are Important for Growth and Development. Vol. 13, *Nutrients.* 2021. p. 4183.

Goran MI, Martin AA, Alderete TL, Fujiwara H, Fields DA. Fructose in Breast Milk Is Positively Associated with Infant Body Composition at 6 Months of Age. *Nutrients.* 2017;9(2):16.

Gridneva Z, Rea A, Lai CT, Tie WJ, Kugananthan S, Warden AH, et al. Human Milk Macronutrients and Bioactive Molecules and Development of Regional Fat Depots in Western Australian Infants during the First 12 Months of Lactation. *Life.* 2022 Mar 28;12(4).

Gridneva Z, Rea A, Tie WJ, Lai CT, Kugananthan S, Ward LC, et al. Carbohydrates in Human Milk and Body Composition of Term Infants during the First 12 Months of Lactation. *Nutrients.* 2019;11(7):28.

Gridneva Z, Tie WJ, Rea A, Lai CT, Ward LC, Murray K, et al. Human Milk Casein and Whey Protein and Infant Body Composition over the First 12 Months of Lactation. *Nutrients.* 2018;10(9):19.

Isganaitis E, Venditti S, Matthews TJ, Lerin C, Demerath EW, Fields DA. Maternal obesity and the human milk metabolome: associations with infant body composition and postnatal weight gain. *Am J Clin Nutr.* 2019;110(1):111–20.

Jacobson JL, Jacobson SW, Muckle G, Kaplan-Estrin M, Ayotte P, Dewailly E. Beneficial effects of a polyunsaturated fatty acid on infant development: evidence from the inuit of arctic Quebec. *J Pediatr.* 2008 Mar;152(3):356–64.

Janas LM, Picciano MF, Hatch TF. Indices of protein metabolism in term infants fed either human milk or formulas with reduced protein concentration and various whey/casein ratios. *J Pediatr.* 1987;110(6):838–48.

Kon IY, Shilina NM, Gmoshinskaya MV, Ivanushkina TA. The study of breast milk IGF-1, leptin, ghrelin and adiponectin levels as possible reasons of high weight gain in breast-fed infants. *Ann Nutr Metab.* 2014 Nov 14;65(4):317–23.

Larnkjaer A, Bruun S, Pedersen D, Zachariassen G, Barkholt V, Agostoni C, et al. Free Amino Acids in Human Milk and Associations With Maternal Anthropometry and Infant Growth. *J Pediatr Gastroenterol Nutr.* 2016;63(3):374–8.

Larson-Meyer DE, Schueler J, Kyle E, Austin KJ, Hart AM, Alexander BM. Appetite-Regulating Hormones in Human Milk: A Plausible Biological Factor for Obesity Risk Reduction? *J Hum Lact.* 2021 Aug;37(3):603–14.

Larsson MW, Lind MV, Larnkjaer A, Due AP, Blom IC, Wells J, et al. Excessive Weight Gain Followed by Catch-Down in Exclusively Breastfed Infants: An Exploratory Study. *Nutrients.* 2018;10(9):12.

Makela J, Linderborg K, Niinikoski H, Yang B, Lagstrom H. Breast milk fatty acid composition differs between overweight and normal weight women: the STEPS Study. *Eur J Nutr.* 2013;52(2):727–35.

Martini, Irwanto, Irawan R, Widjaja NA. Breastmilk macronutrient levels and infant growth during the first three months: A cohort study. *Siriraj Medical Journal.* 2020;72(1):10–7.

Meyer DM, Brei C, Stecher L, Much D, Brunner S, Hauner H. Associations between long-chain PUFAs in maternal blood, cord blood, and breast milk and offspring body composition up to 5 years: follow-up from the INFAT study. *Eur J Clin Nutr.* 2019;73(3):458–64.

Miliku K, Duan QL, Moraes TJ, Becker AB, Mandhane PJ, Turvey SE, et al. Human milk fatty acid composition is associated with dietary, genetic, sociodemographic, and environmental factors in the CHILD Cohort Study. *Am J Clin Nutr.* 2019;110(6):1370–83.

Miller EM. What is significant about a single nursing session? An exploratory study. *Am J Hum Biol.* 2017;29(5):10.

Minato T, Nomura K, Asakura H, Aihara A, Hiraike H, Hino Y, et al. Maternal undernutrition and breast milk macronutrient content are not associated with weight in breastfed infants at 1 and 3 months after delivery. *International Journal of Environmental Research & Public Health*. 2019;16(18):09.

Mitoulas LR, Kent JC, Cox DB, Owens RA, Sherriff JL, Hartmann PE. Variation in fat, lactose and protein in human milk over 24 h and throughout the first year of lactation. *Br J Nutr.* 2002;88(1):29–37.

Much D, Brunner S, Vollhardt C, Schmid D, Sedlmeier EM, Brüderl M, et al. Breast milk fatty acid profile in relation to infant growth and body composition: results from the INFAT study. *Pediatr Res.* 2013 Aug;74(2):230–7.

Mychaleckyj JC, Zhang D, Nayak U, Ross Colgate E, Carmolli M, Dickson D, et al. Association of breast milk gamma-linolenic acid with infant anthropometric outcomes in urban, low-income Bangladeshi families: a prospective, birth cohort study. *Eur J Clin Nutr.* 2019;09:09.

Nikniaz L Jr, Mahdavi R, Arefhoesseini SR, Sowti Khiabani M. Association between fat content of breast milk and maternal nutritional status and infants' weight in Tabriz, Iran. *Malays J Nutr.* 2009;15(1):37–44.

Nuss H, Altazan A, Zabaleta J, Sothern M, Redman L. Maternal pre-pregnancy weight status modifies the influence of PUFAs and inflammatory biomarkers in breastmilk on infant growth. *PLoS One.* 2019 May 29;14(5):e0217085.

Palmer AC, Chileshe J, Hall AG, Barffour MA, Molobeka N, West KP Jr, et al. Short-term daily consumption of provitamin A carotenoid-biofortified maize has limited impact on breast milk retinol concentrations in Zambian women enrolled in a randomized controlled feeding trial. *J Nutr.* 2016;146(9):1783–92.

Peng X, Li J, Yan S, Chen J, Lane J, Malard P, et al. Xiang Study: an association of breastmilk composition with maternal body mass index and infant growth during the first 3 month of life. *Nutr Res Pract.* 2021 Jun;15(3):367–81.

Prentice P, Ong KK, Schoemaker MH, van Tol EA, Vervoort J, Hughes IA, et al. Breast milk nutrient content and infancy growth. *Acta Paediatr.* 2016;105(6):641–7.

Prentice PM, Schoemaker MH, Vervoort J, Hettinga K, Lambers TT, van Tol EAF, et al. Human Milk Short-Chain Fatty Acid Composition is Associated with Adiposity Outcomes in Infants. *J Nutr.* 2019;149(5):716–22.

Riederer M, Wallner M, Schweighofer N, Fuchs-Neuhold B, Rath A, Berghold A, et al. Distinct maternal amino acids and oxylipins predict infant fat mass and fat-free mass indices. *Arch Physiol Biochem.* 2020 Dec 7;1–12.

Rudolph MC, Young BE, Lemas DJ, Palmer CE, Hernandez TL, Barbour LA, et al. Early infant adipose deposition is positively associated with the n-6 to n-3 fatty acid ratio in human milk independent of maternal BMI. *Int J Obes.* 2017;41(4):510–7.

Saben JL, Sims CR, Pack L, Lan R, Børsheim E, Andres A. Infant intakes of human milk branched chain amino acids are negatively associated with infant growth and influenced by maternal body mass index. *Pediatr Obes.* 2022 May;17(5):e12876.

Scholtens S, Wijga AH, Smit HA, Brunekreef B, de Jongste JC, Gerritsen J, et al. Long-chain polyunsaturated fatty acids in breast milk and early weight gain in breast-fed infants. *Br J Nutr.* 2009;101(1):116–21.

Sims CR, Lipsmeyer ME, Turner DE, Andres A. Human milk composition differs by maternal BMI in the first 9 months postpartum. *Am J Clin Nutr.* 2020 Sep 1;112(3):548–57.

Tyson J, Burchfield J, Sentance F, Mize C, Uauy R, Eastburn J. Adaptation of feeding to a low fat yield in breast milk. *Pediatrics.* 1992;89(2):215–20.

Ulloa E, Saure C, Giudici V, Armeno M. Causes of possible excessive weight gain in exclusively breastfed infants in the first six months of life. *Minerva Pediatr.* 2020 Jul 29;

Urteaga N, San Miguel JL, Aguilar AM, Muñoz M, Slater C. Nutritional status and human milk intake of exclusively breast-fed infants at high altitude in La Paz, Bolivia. *Br J Nutr.* 2018 Jul;120(2):158–63.

van Sadelhoff JHJ, Mastorakou D, Weenen H, Stahl B, Garssen J, Hartog A. Short Communication: Differences in Levels of Free Amino Acids and Total Protein in Human Foremilk and Hindmilk. *Nutrients.* 2018;10(12):26.

van Sadelhoff JHJ, Siziba LP, Buchenauer L, Mank M, Wiertsema SP, Hogenkamp A, et al. Free and Total Amino Acids in Human Milk in Relation to Maternal and Infant Characteristics and Infant Health Outcomes: The Ulm SPATZ Health Study. *Nutrients.* 2021 Jun 10;13(6).

Xiang M, Alfven G, Blennow M, Trygg M, Zetterstrom R. Long-chain polyunsaturated fatty acids in human milk and brain growth during early infancy. *Acta Paediatr.* 2000;89(2):142–7.

Xiang M, Lei S, Li T, Zetterstrom R. Composition of long chain polyunsaturated fatty acids in human milk and growth of young infants in rural areas of northern China. *Acta Paediatr.* 1999;88(2):126–31.

Zhang J, Zhao A, Lai S, Yuan Q, Jia X, Wang P, et al. Longitudinal Changes in the Concentration of Major Human Milk Proteins in the First Six Months of Lactation and Their Effects on Infant Growth. *Nutrients*. 2021 Apr 27;13(5).

Appendix A

Medline Search

1 exp Breast feeding/ or Human Milk/ or exp Lactation/ or Colostrum/ (90647)
2 (breast-feed$ or breastfeed$ or breast-fed$ or breastfed$ or breastmilk$ or colostrum).kw,tw. (50890)
3 (milk adj2 (mother$ or maternal or express$ or human or breast$ or share$ or sharing)).kw,tw. (24630)
4 ((nursing or lactating) adj2 (mother$ or maternal or feed$ or infant$ or milk)).kw,tw. (4994)
5 or/1-4 [Breast milk search concept] (116976)

6 exp body weight/ or body composition/ or exp "body weights and measures"/ or growth/ or exp body size/ or growth disorders/ or exp anthropometry/ or Failure to Thrive/ (725377)
7 ((body or muscle) adj2 (composition$ or fat$ or mass$ or size$ or height$ or length$)).kw,tw. (315242)
8 (weight or grow$ or BMI or height or over-weight or overweight or underweight or under-weight or obesity or obese or anthropomet$ or (physical adj1 develop$)).tw,kw. (2833561)
9 or/6-8 [Growth Search Concept] (3206373)

10 Infants/ or Infant, Newborn/ or Infant Health/ or (infant$ or babies or baby$ or newborn or new-born or perinat$ or neonat$).kw,tw. (1433063)

11 exp Dietary Proteins/ or exp Dietary Carbohydrates/ or exp Lipids/ (1287487)
12 exp "amino acids, peptides, and proteins"/ or exp carbohydrates/ or nutrients/ (7763295)
13 (macronutri$ or macro-nutri$ or carbohydrate$1 or fat$ or lactose$ or galactose$ or casein$ or phosphoprotein$ or lipoprotein$ or protein$ or aminoacid$ or taurine$ or lysine$ or tyrosine$ or amino-acid$ or triacylglyceride$ or triglyceride$ or triacylglycerol$ or diglyceride$ or diacylglycerol$ or diacylglyceride$ or monoacylglyceride$ or monoglyceride$ or acylglycerol$ or lipid$ or phospholipid$ or fattyacid$ or acid$ or DHA or EPA or cholesterol$ or LCPUFA$ or PUFA$ or omega-3 or omega-6 or glycoprotein$ or glycolipid$ or glucose$ or disaccharide$).kw,tw. (5737284)
14 or/11-13 [Macronutrient search concept] (10146645)
15 5 and 9 and 10 and 14 (7079)

16 exp Micronutrients/ or Calcium, Dietary/ or Phosphorus, Dietary/ or magnesium/ or exp Vitamin A/ or carotenoids/ or choline/ or Iron, Dietary/ or exp Elements/ or exp Folic Acid/ or Dietary Potassium/ (2001860)
17 (micro-nutri$ or micronutri$ or provitamin$ or pro-vitamin$ or previtamin$ or zinc or calcium$ or phosphorus$ or magnesium$ or iodine$ or selenium$ or carotenoid$ or thiamine$ or riboflavin$ or folate$ or choline$ or iron$ or niacin or nicotinamide$ or mineral$ or retinol$ or potassium).kw,tw. (1228280)
18 (vitamin adj1 (a$1 or b$2 or c$1 or d$1 or e$1)).tw,kw. (161743)
19 or/16-18 [Micronutrient Search] (2733776)
20 5 and 9 and 10 and 19 (2885)
21 exp immunoglobulins/ or exp oligosaccharides/ or exp Hormones/ (2270362)
22 (bio-active$ or bioactive$ or immune-globulin$ or immunoglobulin$ or antibod$ or anti-bod$ or monoligosacchari$ or mono-oligosacchari$ or monosacchari$ or oligosacchari$ or HMO or

fucosyllactose$ or sialyllactose$ or ig$2 or cytokine$ or interleukin$ or interferon-g or lactoferrin$ or lactotransferrin$ or osteopontin$ or bone-sialoprotein or hormone$ or leptin$ or insulin$ or adiponectin$ or ghrelin$ or "tumo?r necrosis factor-a" or TNF-a$).kw,tw. (2386179)

23 (transforming-growth-factor$ or tumo?r-growth-factor$ or TGF$ or epidermal-growth-factor$ or urogastrone$).kw,tw. (166992)

24 or/21-23 [Bioactive Search] (3637852)

25 5 and 9 and 10 and 24 (3101)

26 15 or 20 or 25 (8686)

27 26 not (exp Animals/ not humans.sh.) (7205)

28 limit 27 to english language (6683)

29 limit 28 to yr="1980 -Current" (6365)
